# A Conserved Glycan in the C2 Domain of HIV-1 Envelope Acts as a Molecular Switch to Control X4 Utilization by Clonal Variants with Identical V3 Loops

**DOI:** 10.1371/journal.pone.0128116

**Published:** 2015-06-17

**Authors:** Francesca Lombardi, Kyle J. Nakamura, Thomas Chen, Edwin R. Sobrera, Nicole H. Tobin, Grace M. Aldrovandi

**Affiliations:** Department of Pediatrics, Children’s Hospital Los Angeles, Los Angeles, CA, United States of America; University of Pittsburgh Center for Vaccine Research, UNITED STATES

## Abstract

Nearly all persons newly infected with HIV-1 harbor exclusively CCR5-using virus. CXCR4-using variants eventually arise in up to 50% of patients infected with subtypes B or D. This transition to efficient CXCR4 utilization is often co-incident with progression to AIDS. The basis for HIV-1’s initial dependence on CCR5, the selective force(s) that drive CXCR4-utilization, and the evolutionary pathways by which it occurs are incompletely understood. Greater knowledge of these processes will inform interventions at all stages, from vaccination to cure. The determinants of co-receptor use map primarily, though not exclusively, to the V3 loop of gp120. In this study, we describe five clonal variants with identical V3 loops but divergent CXCR4 use. Mutagenesis revealed two residues controlling this phenotypic switch: a rare polymorphism in C1 and a highly conserved N-glycan in C2. To our knowledge, this is the first description of co-receptor usage regulated by the N-glycan at position 262.

## Introduction

The entry of HIV-1 into susceptible host cells is mediated by the viral envelope glycoprotein (Env). The functional envelope spike is comprised of three non-covalently associated gp41 transmembrane sub-units and three gp120 surface units. Each gp120 molecule is divided into five constant (C1-C5) and five variable (V1-V5) domains, and contains a binding site for the primary cell-surface receptor CD4. Following CD4-engagement, the envelope undergoes a conformational change that exposes or creates a binding site for its co-receptor, typically CCR5 (R5) or CXCR4 (X4). Binding to the co-receptor triggers conformational changes in gp41 that ultimately result in fusion of the virus and host-cell membranes [reviewed in [1].

Virtually all HIV-1 infections are established by exclusively R5-using virus, regardless of the presence of R5/X4 or obligate X4 virus in the index case [2, 3]. Furthermore, approximately 1–2% of persons of Northern European descent are homozygous for a 32 base pair deletion in the CCR5 gene (CCR5Δ32) which alters CCR5 expression on the surface of their cells and renders them highly resistant to HIV. Persons heterozygous for CCR5Δ32 have lower cell-surface expression of CCR5, are partially resistant to infection, and tend to progress slower if infected [4, 5].

This rigid constraint on an otherwise fluid and rapidly evolving virus has led to the development and testing of many interventions targeting CCR5 for prevention, treatment, and even cure [6–10]. Unfortunately, these efforts are at risk of failure should the virus successfully transition to efficient X4 utilization [11–13]. A better mechanistic understanding of the R5-to-X4 transition will allow scientists and clinicians to better predict, and potentially counter, such an escape strategy.

The determinants of co-receptor usage map primarily to the V3 loop; which makes extensive molecular contacts with the co-receptor [14–16]. It has been well-established that an overall shift towards positive charge, but especially positively charged substitutions at positions 11, 24, and 25 in V3 are predictive of X4 utilization [17, 18], as is the presence of an isoleucine at position 326 in the V3 stem [19, 20]. Several algorithms have been developed to predict co-receptor usage based on V3 sequence, with accuracies estimated at 70–80% [21]. However, substitutions in other regions including the bridging sheet, C4, V1/V2, and gp41 have also been shown to influence co-receptor usage [22–27].

For this study, we screened a cohort of treatment-experienced, predominantly subtype-B infected subjects failing their current ARV regimens, using resistance to the CCR5-antagonist maraviroc (MVC) as an initial surrogate marker for efficient X4 utilization. We reasoned that this population would be more likely to harbor the X4 using or transitional variants of interest. Convenience sampling of ten subjects identified three with MVC-resistant virus. From one of these three, we isolated a series of closely related molecular envelope clones with identical V3 sequences but highly variable co-receptor usage. Further *in vitro* characterization revealed that X4 utilization was regulated by polymorphisms in C1 and C2. The C2 polymorphism disrupted a conserved potential N-linked glycosylation site (PNG) important for envelope function but not previously linked to co-receptor selectivity.

## Materials and Methods

### Study Population

All subjects were treatment experienced and screened for, but unable to enroll in, IMPAACT protocol P1020a [28]. Written informed consent was obtained by study candidate, parent or legal guardian prior to screening and recorded per protocol. The study was approved by the Institutional Review Boards at each investigator site (see listing in acknowledgements and manuscript PMID 25232777) and registered with ClinicalTrials.gov, Identifier NCT00006604. No subjects had prior exposure to entry inhibitors (including Maraviroc).

### Cells and Reagents

293T/17 retroviral packaging cells were obtained from the American Type Culture Collection (ATCC, cat# CRL-11268). TZM-bl cells, a HeLa clone expressing high levels of CD4, CCR5, and CXCR4 as well as ß-galactosidase and firefly luciferase reporter genes under the control of the HIV promoter [29–33], were obtained from the NIH AIDS Reagent Program (ARRRP), Division of AIDS, NIAID, NIH from Dr. John C. Kappes, Dr. Xiaoyun Wu, and Tranzyme Inc. (cat# 8129). Parental GHOST cells, as well as GHOST-R5, GHOST-X4, and GHOST-R3/X4/R5 subclones were obtained from the ARRRP (cat#s 3679, 3944, 3685, and 3943) from Dr. Vineet N. Kewal Ramani and Dr. Dan R. Littman [34]. All cell lines were maintained in Dulbecco’s Modified Eagle’s Medium (DMEM; Fisher Scientific, Waltham, MA) supplemented with 10% fetal bovine serum (FBS; Gemini Bio-products, Sacramento, CA), 100 U/mL penicillin-streptomycin (Gibco, Invitrogen, Carlsbad, CA), and 2 mM L-glutamine (Gibco). GHOST cells were supplemented with 500 μg/mL G418 (Invitrogen) and 100 μg/mL hygromycin (Invitrogen), and 1 μg/mL puromycin (GHOST-R5, GHOST-X4, and GHOST-R3/X4/R5; Sigma-Aldrich, St. Louis, MO).

MVC (Selzentry) was generously donated by Pfizer. TAK779 [35], AMD3100 [36–38], and T20/Fuzeon (courtesy of Roche) were obtained from the ARRRP (cat#s 4983, 8128, and 9845). AD-101 (SCH-350581) was generously donated by Schering-Plough. Soluble lectins were purchased from commercial suppliers: Vector Laboratories Inc. [Burlingame, CA; *Hippeastrum* hybrid (Amaryllis) agglutinin (HHA) and *Galanthus nivalis* agglutinin (GNA), cat #s L-1240 and L-1380] and EY Laboratories [San Mateo, CA; *Urtica dioica* agglutinin (UDA), cat# L-8005-1].

### Cloning and Mutagenesis

RNA was extracted from plasma using QIAamp Viral RNA Mini Kit (Qiagen, Valencia, CA). Following reverse transcription using the SuperScript III system (Invitrogen) and the primer envB3out (5′-TTGCTACTTGTGATTGCTCCATGT-3′), cDNAs were serially diluted until <30% of reactions were positive as previously described [39]. Full-length gp160s were amplified by nested PCR using high-fidelity polymerase and the primers envB5out (5′-TAGAGCCCTGGAAGCATCCAGGAAG-3′)/envB3out for the first round, and envB5in (5′-TTAGGCATCTCTATGGCAGGAAGAAG-3′)/envB3in (5′-GTCTGAGATACTGCTCCCACCC-3′) for the second. Cycle timings were: first round [94°C × 2 min, 35 cycles of (94°C × 15 sec, 55°C × 30 sec, 68°C × 4 min), and 68°C × 20 min] and second round [94°C x 2 min, 45 cycles of (94°C × 15 sec, 55°C × 30 sec, 68°C × 4 min), and 68°C × 20 min]. Amplicons were ligated into the pcDNA 3.1 Topo Expression vector (Invitrogen) and transformed into TOP10 E. Coli (Invitrogen) using standard molecular biology techniques.

Envelope clones were sequenced bi-directionally, and the contigs assembled and edited using Sequencher (Gene Codes, Ann Arbor, MI). Alignments were constructed using MacVector (MacVector Inc, Cary, NC) and the LANL website tools (http://www.hiv.lanl.gov/content/sequence/HIV/HIVTools.html). All amino acid residues are numbered based on the HXB2 reference sequence. Point mutations were introduced by site-directed mutagenesis (QuikChange multi-site directed mutagenesis kit; Stratagene, La Jolla, CA) according to the manufacturer’s instructions and sequenced to confirm integrity. For the remainder of the manuscript, un-mutated envelopes derived from subject plasma will be referred to as ‘parental’.

Protease inhibitor (PI), nucleoside reverse transcriptase inhibitor (NRTI), and non-nucleoside reverse transcriptase inhibitor (NNRTI) resistance data were obtained from the IMPAACT P1020 screening records and were determined using the ViroSeq HIV-1 Genotyping System (ABI/Life Technologies, Foster City, CA) and the Stanford HIV drug resistance database (http://hivdb.stanford.edu/).

The V3 loop sequence of each envelope was analyzed using the web-based tools Geno2pheno (http://co-receptor.bioinf.mpi-inf.mpg.de/index.phpare), position-specific scoring matrix (PSSM, http://indra.mullins.microbiol.washington.edu/webpssm), and the 11KR/25KR and 11/24/25 net charge rules to predict co-receptor usage [17, 18, 40–42]. These methods classify HIV-1 strains as either R5 or X4. The latter designation includes variants that use X4 exclusively as well as those capable of using both R5 and X4 for entry.

### Virus Pseudotyping

The env-deficient HIV-1 genome plasmids SG3ΔEnv (cat# 11051) and pNL4-3.Luc.R^-^.E^-^ (carrying a luciferase reporter gene; cat# 3418) were obtained through the AIDS Research and Reference Reagent Program, Division of AIDS, NIAID, NIH from Dr. John C. Kappes and Dr. Xiaoyun Wu [31, 43], and Dr. Nathanial Landau [44, 45] respectively. The backbone plasmid bearing the green fluorescence protein (GFP) reporter gene, NLENG1-ES-IRES, was kindly provided by Dr. David Levy [46, 47].

### Inhibition and Entry Assays

Pseudoviruses were produced by co-transfection of 293T/17 cells with the patient-derived Env-expressing plasmids, and either SG3Δenv, pNL4-3.Luc.R^-^E^-^ or NLENG1-ES-IRES and titered by X-gal staining of TZM-bl cells as previously described [48].

All cell entry assays (no primary cell data were included) were performed using pseudotype virus in a 96-well plate format as previously described [48]. Briefly, using titers obtained by X-gal staining, ~2000 units of pseudovirus in 16 μg/mL DEAE Dextran were added to each well containing target cells. Luminescence was quantified after a 48-hour incubation using a Promega Luciferase Kit (Promega, Madison, WI) and a FLUOstar luminometer (BMG Labtech, Cary, NC) according to manufacturer instructions.

Phenotypic determination of co-receptor utilization was performed by infecting TZM-bl cells in the presence or absence of AMD3100 (X4 antagonist) or TAK-779 (R5 antagonist), and by infection of GHOST cells (expressing CD4 and one or both of the co-receptors) with pNL4-3.Luc.R^-^E^-^derived pseudoviruses.

Drug sensitivity was determined by pre-treatment of TZM-bl cells for 1 hour at 37°C with increasing concentrations of the CCR5 antagonists MVC, TAK-779, AD101, the X4 inhibitor AMD3100, the fusion inhibitor T20, or the lectins HHA, GNA, or UDA prior to addition of pseudovirus.

### Data Analysis

Drug sensitivity was quantified by calculating a mean 50% inhibitory concentration (IC_50_) for each envelope/drug combination with the Prism 4 software (GraphPad, San Diego, CA) using data from at least two experimental replicates. Co-receptor usage, based on TZM or GHOST cell methods was expressed as a percentage normalized to entry in the absence of inhibitors in cells expressing both co-receptors (GHOST-R3/X4/R5 for that platform).

## Results

### Clinical Characteristics of Study Subjects

The clinical characteristics of our cohort are listed in Table 1. All subjects were experiencing virologic failure (median plasma HIV RNA of 106,808 copies/ml) on their current ARV regimen, with at least moderate resistance to two or more drug classes as determined by genotypic analysis using the Stanford HIV drug resistance database. Of the ten subjects included in our initial round of screening, nine were infected with subtype B and one (subject 1002) was infected with subtype C HIV-1.

**Table 1:** Clinical characteristics of study subjects.

Patient ID	Subtype	HIV Plasma RNA (copies/mL)	CD4 Count (cells/μL) [CD4 Percent]^1^	Protease Inhibitor Susecptibility^2^	Nucleoside RT Inhibitor Susceptibility^2^	Non-nucleoside RT Inhibitor Susceptibility^2^	Predicted Tropism (V3 Loop)	Maraviroc Susceptibility^3^
Subj. 1001	B	119,407	NA	Resistant	Resistant	Resistant	R5	Susceptible
Subj. 1002	C	51,400	2422 [39%]	Resistant	Intermediate	Resistant	R5	Susceptible
Subj. 1003	B	140,308	NA	Resistant	Resistant	Resistant	R5	Susceptible
Subj. 1004	B	29,959	NA	Resistant	Intermediate	Resistant	R5	Susceptible
Subj. 1005	B	1,197,844	NA	Intermediate	Resistant	Intermediate	R5/X4	Susceptible/**Resistant**
Subj. 1006	B	5,567	751 [35%]	Intermediate	Intermediate	Susceptible	R5	Susceptible
Subj. 1007	B	7,474	NA	Intermediate	Intermediate	Susceptible	R5	Susceptible
Subj. 1008	B	684,733	NA	Resistant	Intermediate	Resistant	R5	Susceptible
Subj. 1009	B	110,415	NA	Resistant	Intermediate	Resistant	X4	**Resistant**
Subj. 1010	B	103,200	666 [15%]	Resistant	Resistant	Resistant	X4	Susceptible/**Resistant**

^1^ Some subjects were identified as ineligible for P1020 before a CD4 count was performed

^2^ Anti-retroviral drug susceptibility was determined genotypically using the Stanford HIV Drug Resistance Database

^3^ Maraviroc susceptibility cut-offs reflect the ability to achieve 50% neutralization at the highest drug concentration tested (200nM)

### Identification of a Subject with both MVC-Resistant and MVC-Sensitive Virus

Full-length molecular envelope clones were generated from the plasma of all ten subjects by RT-PCR and fully sequenced. V3 loops were analyzed using the web-based algorithms Geno2Pheno, PSSM, and 11/24/25. Pseudotype viruses were generated from all envelopes and tested for resistance to MVC, which served as a surrogate marker for X4 utilization at the screening stage. Data are presented in Table 2.

**Table 2:** V3 loop sequences, genotypic characteristics, and R5/X4-usage screening data for all clones discussed in the study.

Subject ID	Clone ID	V3 loop sequence	Position 11/25	V3 loop Pos. Charge	V3 loop Net Charge	MRV IC_50_ (nM)	Coreceptor Usage Efficiency	
							R5%	X4%
1001	1001	CTRPNNNTRKGIQMGPGKAFYATGEIIGDIRQAHC	G/E	6	4	7.5	84.4	1.2
	1003	-----------------------------------	G/E	6	4	6.1	93.6	1.2
	1004	-----------------------------------	G/E	6	4	6.7	91.2	0.8
	1005	-----------------------------------	G/E	6	4	7.7	85.7	1.1
	1006	-----------------------------------	G/E	6	4	5.6	87.1	1.0
	1008	-----------------------------------	G/E	6	4	6.2	88.5	0.6
	1010	-----------------------------------	G/E	6	4	6.6	91.1	1.9
	1012	-----------------------------------	G/E	6	4	6.1	83.9	1.9
1002	902	CTRPNNNTRKSIRIGPGQTFYATGGIIGNIRQAHC	S/G	6	5	2.8	94.3	0.1
	905	-----------------------------------	S/G	6	5	3.1	98.8	0.1
	907	-----------------------------------	S/G	6	5	3.3	90.9	0.2
	912	-----------------------------------	S/G	6	5	3.2	86.4	0.1
	9A104	-----------------------------------	S/G	6	5	1.0	96.7	0.1
	9A105	-----------------------------------	S/G	6	5	4.1	90.0	0.5
	9A106	-----------------------------------	S/G	6	5	3.5	92.2	0.3
	9A107	-----------------------------------	S/G	6	5	3.7	91.9	0.1
	9A108	-----------------------------------	S/G	6	5	1.3	89.6	0.1
	9A204	-----------------------------------	S/G	6	5	3.0	90.0	0.4
	9A408	-----------------------------------	S/G	6	5	3.9	87.6	0.1
	9A208	-----------------------------------	S/G	6	5	3.0	95.4	0.2
	9A405	-----------------------------------	S/G	6	5	3.9	91.5	0.1
1003	740	CTRPNNNTRKGIQMGPGKAFYATGEIIGDIRQAHC	G/E	6	4	6.3	89.6	1.2
	1931	-----------------------------------	G/E	6	4	8.8	83.9	1.3
	1932	-----------------------------------	G/E	6	4	4.8	87.3	1.1
	1934	-----------------------------------	G/E	6	4	6.2	87.2	1.0
1004	4801	CTRPNNNTRKSINIGPGRAFYTTGEVIGNIRQAHC	S/E	7	6	2.2	89.5	0.6
	4802	-----------------------------------	S/E	7	6	2.0	90.1	0.5
	4803	-----------------------------------	S/E	7	6	2.1	90.3	0.5
	4809	-----------------------------------	S/E	7	6	2.3	90.5	0.4
1005	4007	CIRPNNNTRQRLSIGPGRSFYATRQIVGNIRQAHC	R/Q	7	7	>200	60.6	83.1
	4022	---------KSIG-----A----GD-I-D------	S/D	6	4	3.2	99.8	0.1
1006	102	CTRPNNNTRKSIHIGQGRALYATGQIIGDIRQAHC	S/Q	7	6	2.6	79.8	0.1
1007	1406	CERPNNNTRKSIHLGPGGAFYATGNIIGDIRKAHC	S/N	7	5	1.8	99.9	0.3
	1047	-----------------------------------	S/N	7	5	2.4	98.2	0.2
	1052	-----------------------------------	S/N	7	5	2.4	96.3	0.2
	562	------------------------R----------	S/R	8	6	1.7	89.9	0.1
	567	------------------------R----------	S/R	8	6	2.1	95.1	0.5
	1044	------------------------R----------	S/R	8	6	2.2	95.0	0.1
	1211	----------------R------------------	S/N	8	6	1.7	98.1	0.9
	564	----------------R------------------	S/N	8	6	2.3	93.7	0.2
1008	1591	CTRPSNNTRRGIHMGPGK_FYTTGQIIGDIRQAHC	G/Q	7	6	6.6	89.9	1.1
1009	665	CTRPTNYTSKMIRIQRGPGRAYVRTRPSGDIRQAYC	M/T	7	6	>200	0.5	100.0
	1692	-----------------------------------	M/T	7	6	>200	0.5	94.1
	2120	---------------------F---K----------	M/T	7	6	>200	0.6	100.0
	2122	---------------------F---K----------	M/T	7	6	>200	0.5	85.9
	2003	---------------------F---K----------	M/T	7	6	>200	0.7	100.0
	2009	---------------------F---K----------	M/T	7	6	>200	0.3	100.0
	2342	---------------------F---K----------	M/T	7	6	>200	0.5	98.7
1010	550	CIRPNNNTRKGITLGPGRVFYTTGQIVGDIRKAHC	C/Q	7	6	2.2	87.5	9.5
	542	-----------------------------------	C/Q	7	6	1.8	85.5	9.9
	543	-----------------------------------	C/Q	7	6	>200	100.0	56.9
	544	-----------------------------------	C/Q	7	6	>200	88.8	59.9
	547	-----------------------------------	C/Q	7	6	>200	98.7	50.0

Viral envelopes isolated from seven of the ten patients screened were predicted *in silico* to be exclusively R5-using and were phenotypically sensitive to MVC, thus no further studies were performed on them. Seven clones isolated from subject 1009 were predicted to use X4 by both Geno2Pheno and PSSM and were highly resistant to MVC. Two clones were isolated from subject 1005 with highly divergent V3 loop sequences, one of which was predicted to use X4 by Geno2Pheno and PSSM and was MVC-resistant, while the other was predicted to use X4 by Geno2Pheno but R5 by PSSM, and was sensitive to MVC. Finally, five clones were isolated from subject 1010, all with identical V3 loop sequences that were predicted to use X4 by Geno2Pheno but R5 by PSSM. Two of those clones were sensitive to MVC (IC_50_ ~2nM) while the other three were highly resistant (IC_50_ >200nM). We chose to focus on the isolates from this last subject for further examination. These data are summarized in Table 2.

### Identification of Non-V3 Loop Co-receptor Use Determinants in Subject 1010

**Fig 1:**
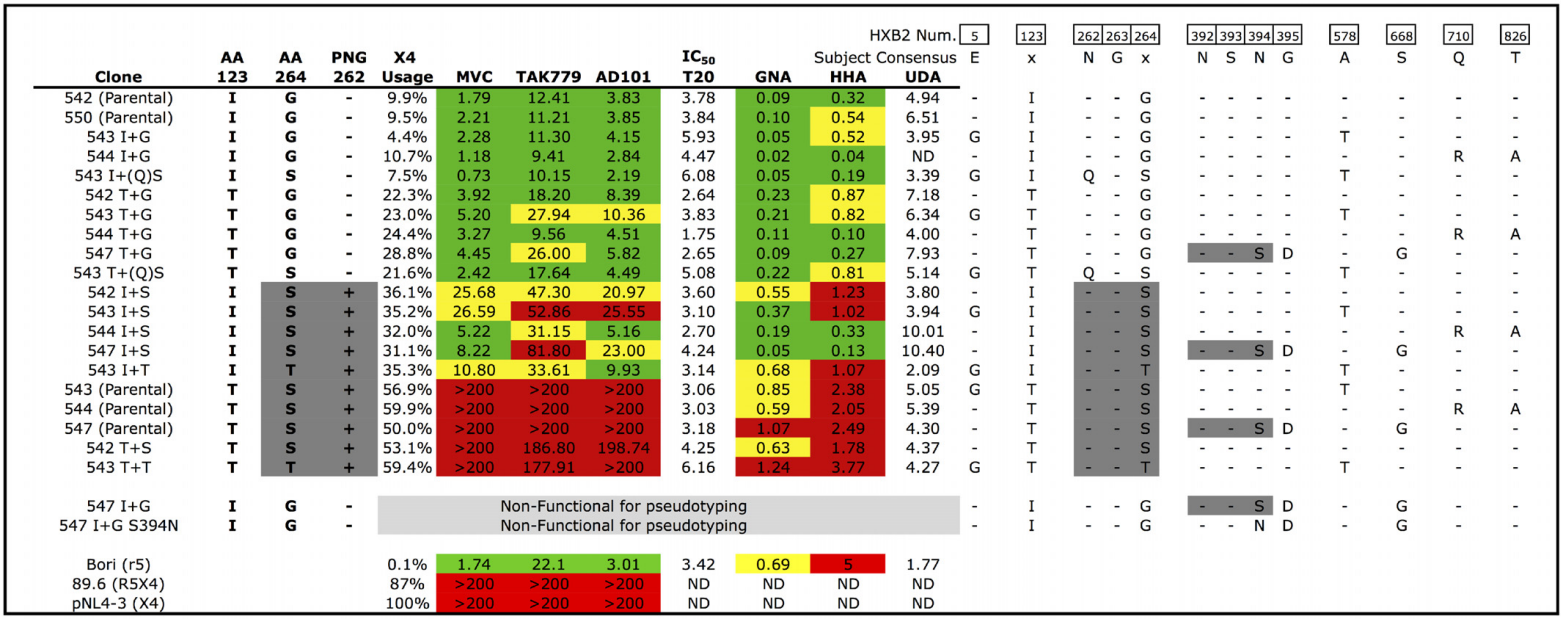
Partial alignment and phenotypic data for all parental and mutants envelopes from subject 1010. Mutant sequences are identified by the parental clone (i.e. 542) and the residues present at positions 123 and 264 (i.e. 543 I+G indicates a mutant derived from parental clone 543 with an I at position 123 and a G at position 264). The presence or absence of a potential N-linked glycosylation site (PNG) at position 262 (as determined by the N-glycosite web tool from LANL) is denoted by a + or—in the column labeled PNG 262, and the relevant sequons [Nx(S/T)] are shaded dark gray in the abbreviated alignment on the right. Efficiency of entry into CXCR4-expressing (X4) GHOST cells [relative to CCR5/CXCR4-expressing (R5/X4) GHOST cells] is indicated for each clone, as are fifty-percent inhibitory concentrations (IC_50_) in nM for the R5 inhibitors Maraviroc (MVC), TAK779, and AD101; the peptide fusion inhibitor T-20; and in μg/ml for the plant-derived lectins GNA, HHA, and UDA. Columns have been color-coded to highlight the concentration of a given compound that is required to achieve the IC50. Expressing cut-offs for green, yellow, and red coding: for MVC and AD101:0–9.9, 10–24.9, >25nM; for TAK-779: 0–24.9, 25.0–49.9, >50nM; and for GNA and HHA: 0–0.49, 0.5–0.99, and >1.0 μg/ml. T20 and UDA were not color coded, as their IC50s did not correspond to the % X4 usage of the clone. Phenotypic data for reference viruses Bori, 89.6, and pNL4-3 are shown at the bottom of the figure.

The five *env* clones from subject 1010, which had identical V3 sequences, differed at nine positions throughout the remainder of gp160 (five residues in gp120 and four in gp41; Fig 1). Only two positions correlated with MVC sensitivity. The first was the highly conserved bridging sheet residue 123, at which the MVC-sensitive isolates had an unusual isoleucine substitution for the conserved threonine (T123I). Polymorphisms at this site were present in only 6 of 1501 complete subtype B sequences retrieved from the 2012 LANL database (two T123I and four T123A). The second position was the highly conserved serine 264 in the C2 domain, which was mutated to a glycine in the MVC-sensitive isolates (S264G). Polymorphisms at this site were similarly rare, present in only 7 of 1501 subtype B LANL sequences (one S264G, one S264N, three S264T, and two nonsense mutations). For simplicity, envelopes will be referred to by the amino acids at positions 123 and 264 (i.e. I+G or T+S) for the remainder of the manuscript (as shown in Fig 1). It is important to note here that serine 264 is part of a PNG sequon, [NX(S/T)] including the conserved asparagine at 262, which is disrupted by the S264G polymorphism. This glycan has been localized to the outer domain of gp120, where it forms a glycan cluster with N295, N332, and N448 [most recently in the structure described in [49]].

To verify that these isolates were indeed using X4 for entry and not simply able to productively utilize MVC-bound CCR5 as described in [50], they were tested on cells pre-treated with two additional CCR5 inhibitors, TAK779 and AD101, to which they were also resistant. Envelopes were also tested for entry into GHOST cells expressing CD4 and either CCR5 or CXCR4 alone, with results concordant with those obtained from the TZM-based inhibitor assays (summarized in Fig 1 **)**. These results confirmed that we identified clonal envelope variants with highly divergent X4 usage regulated by polymorphisms outside the V3 loop.

### Site-Directed Mutagenesis Reveals a Novel Regulator of X4 Usage

We created a panel of mutant envelopes in which the residues at 123 and 264 were exchanged individually and in combination between the MVC-resistant/X4-using (T+S) and MVC-sensitive/R5-using (I+G) variants. This panel was screened using MVC, TAK779, AD101, AMD3100, and assayed for entry into R5- and X4-expressing GHOST cells. Results are presented in Fig 1.

The exchange of both residues (123 and 264) was sufficient to confer MVC-resistance and X4-utilization on the sensitive/R5 envelopes, and vice versa. The intermediate genotype T+G (which lacked a PNG at 262) was relatively sensitive to R5 inhibitors, but demonstrated elevated efficiency of X4-usage compared to the fully MVC-sensitive envelopes (I+G). In contrast, the intermediate genotype I+S (which possessed a PNG at 262) was relatively resistant to R5 inhibitors and had an X4-utilization efficiency much closer to the fully MVC-resistant (T+S) clones. These data indicate that exchange of residues at positions 123 and 264 is sufficient to confer full X4-tropism, and that position 264 is the dominant molecular switch.

Only one mutant, 547 I+G, was non-functional for pseudotyping in 293 cells. I+G mutants made using the other parental X4 envelopes (543 I+G and 544 I+G) were fully functional, as was the mutant 547 T+G. The most apparent difference between the parental 547 and 543/544 envelopes is the presence of a conserved PNG at position 392 in clone 547 that is absent in 543/544. Interestingly, this PNG was also absent in the parental R5 variants (542/550), both of which have the I+G genotype. To determine whether a PNG at position 392 was functionally incompatible with the I+G genotype we constructed a second round of mutants, disrupting the PNG sequon with an S394N substitution in the non-functional mutant 547 I+G and adding a PNG site to clone 542 (a parental I+G) at position 392 via the reciprocal N394S substitution. Surprisingly mutant 547 I+G S394N remained non-functional for pseudotyping, while mutant 542 N394S retained function and was similarly sensitive to R5 inhibition as the parental 542 variant (data not shown). The functional and non-functional variants differed at only two additional residues, a glycine-to-aspartic acid at position 395, and a serine-to-glycine at position 668 in the gp41 membrane-proximal external region.

Changes in T20 susceptibility, in the absence of gp41 mutations that directly affect binding, have been associated with altered fusion kinetics [51]. T20 binds to the fusion-intermediate conformation of gp41 and prevents final collapse of the gp41 trimer into the 6-helix bundle required for membrane fusion and entry [52]. To determine whether any of our parental or mutant envelopes had gross defects in their entry kinetics, we tested the full panel for susceptibility to T20. We did not observe any significant differences in T20 susceptibility between R5 and X4 variants, nor associated with any of our in vitro mutants (Fig 1).

### Glycosylation of Asparagine 262, Rather Than the Specific Identity of the Residue at Position 264, Controls Co-Receptor Usage and Effects Neutralization by Mannose-, but Not Complex Glycan-, Dependent Soluble Lectins

To confirm that the S264G substitution was indeed acting by elimination of the glycan attached to asparagine 262, we constructed a set of mutants designated I+(Q)S and T+(Q)S, in which the PNG sequon was disrupted by an N262Q substitution that left the serine at position 264 intact. The I+(Q)S and T+(Q)S mutants behaved identically to the I+G and T+G variants (all of which lack a PNG at 262). We constructed other mutants, designated I+T and T+T, in which the serine at position 264 was changed to a threonine, preserving the PNG sequon. The I+T and T+T mutants behaved like the I+S and T+S variants (all of which have a PNG at 262). These data confirm that it is the presence of a PNG at position 262, and not the specific identity of the residue at position 264, that regulates co-receptor usage in these clonal variants.

**Fig 2:**
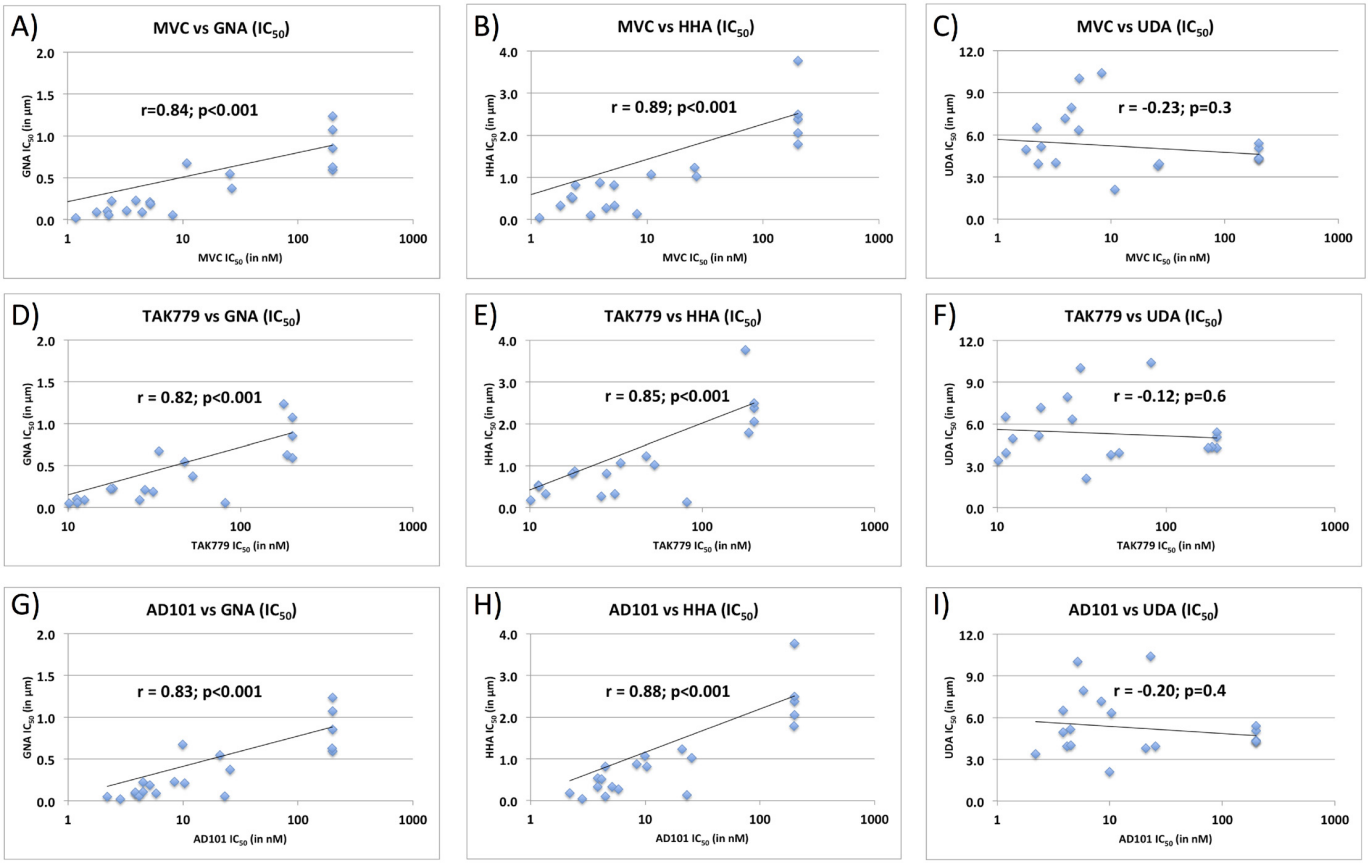
Correlation of R5 inhibitor susceptibility and high mannose specific (GNA/HHA) or complex glycan specific (UDA) lectin susceptibility. For each parental (n = 5) and mutant (n = 15) clone from patient 1010, the 50% inhibitory concentration (IC_50_, in nM) of maraviroc (A-C), TAK779 (D-F), or AD101 (G-I) is plotted on the x-axis and the IC_50_s (in μm) for the high mannose specific lectin GNA (A,D,G), the high mannose specific lectin HHA (B,E,H), and the complex glycan specific lectin UDA (C,F,I) are plotted on the y-axis. Pearson correlation coefficients (r) and associated p-values are also displayed.

We also tested the parental and mutant envelopes with the soluble plant-derived lectins GNA, HHA, and UDA. GNA and HHA both bind high-mannose glycans, while UDA binds complex-type glycans [53]. All three lectins neutralized all tested envelopes (Fig 1). Activity of the two mannose-dependent lectins, GNA and HHA, correlated well with the activity of the R5 antagonists (R^2^ 0.68–0.79): the most lectin-resistant variants had the T+S genotype, the moderately resistant variants the I+S genotype, and the most sensitive had either the I+G or T+G genotypes. Resistance to the complex-glycan-dependent lectin UDA did not vary significantly among any of our isolates, nor correlated with the activity of any of the R5 antagonists (R^2^ 0.01–0.05). It is important to note that previously published biochemical analyses indicate that the glycan at position 262 is predominantly high mannose when produced in 293 cells [54, 55]. Correlation plots are presented in Fig 2.

## Discussion

In this study, we analyzed a series of clonal HIV-1 envelope variants with identical V3 loops but varied co-receptor usage. We found that substitutions at conserved residues in the bridging sheet (position 123) and the C2 domain (PNG at position 262) were responsible for regulating X4 usage in these isolates. To our knowledge, neither of these positions has been previously identified as a regulator of co-receptor utilization.

It is noteworthy that in this case the atypical (I+G) variants are the more phenotypically ‘normal’ (MVC-sensitive and R5-using), while the genotypically normal (T+S) variants are phenotypically unusual (MVC-resistant with efficient X4-utilization). This limits our ability to draw broad conclusions about X4 evolution in general, but does point to a previously unappreciated role of the N-glycan at 262 in governing the conformation or exposure of the co-receptor binding site. Previous studies have identified this glycan as important for viral infectivity [56, 57], and it will be of interest to determine what compensatory mutations have emerged in these naturally occurring variants.

The lack of effect resulting from exchange of the PNG at 392 between functional and non-functional I+G variants was surprising to us. The conserved N-glycan at position 392 is present in only one of the three X4-using clones and its absence does not affect any measure of co-receptor usage that we tested. Additional studies will be required to determine what functional relationship exists between PNG 392 and positions 395/668, and whether those interactions are strain-specific or generalizable.

One limitation of our study is the lack of longitudinal sampling. While it is very likely this subject was originally infected with an R5 virus, we cannot be certain without testing earlier samples. If this were indeed the case, it would be of interest to know whether the transmitted isolate possessed a PNG at 262 and, if not, how the acquisition of that PNG is temporally related to the emergence of a strong X4 phenotype. Moreover, if the transmitted isolate was indeed exclusively R5-using and evolved to utilize X4 while still possessing a PNG at 262, then dissecting the selective forces that favored some variants loosing their ability to use X4, as well as the conserved PNG at 262, may shed more light on the underlying biology of co-receptor selection.

**Fig 3:**
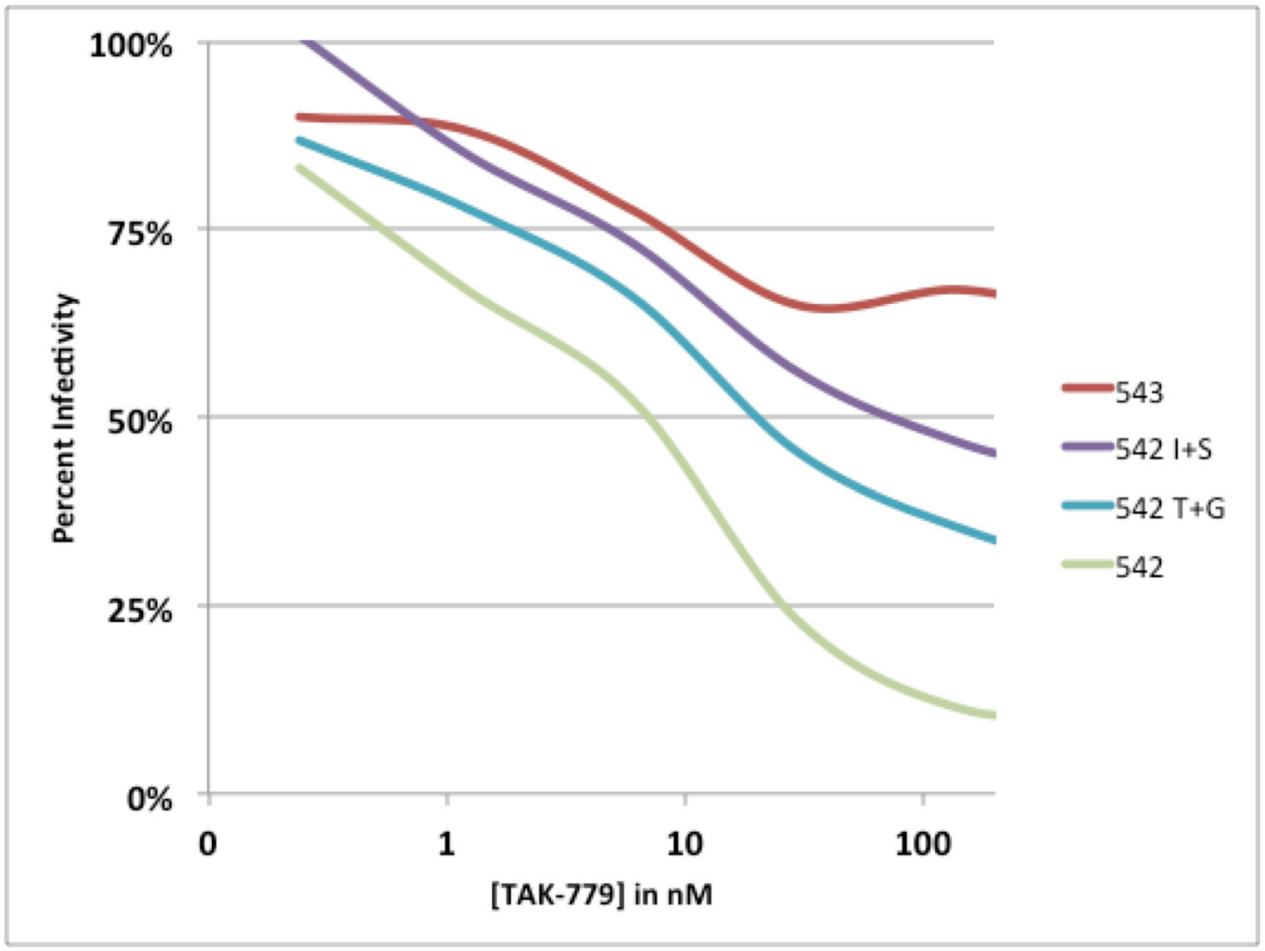
Exemplar neutralization curve showing plateau effect for the CCR5 antagonist TAK779. Clones representing the four core genotypes described in the study [543 parental (T+S), 542 parental (I+G), 542 I+S mutant, and 542 T+G mutant] were tested for neutralization using the R5 antagonist TAK779 (concentration of TAK779 is shown on the X-axis and percent infectivity on the Y-axis).

It is also of interest that the X4 clones from this subject (both parental and mutant) were relatively sensitive to all three CCR5 antagonists (MVC, TAK779, and AD101), not achieving an IC50 >200nM until the strain had >50% predicted X4 usage (see Fig 1). Examination of TZM neutralization curves (exemplar shown in Fig 3) indicated that both R5 inhibitor-sensitive and R5 inhibitor-resistant variants had similar slopes but reached plateaus at the approximate maximum-percent-inhibition predicted by the GHOST cell assay (hence the inability to determine an IC_50_ value for envelopes which used X4 very efficiently). Given that MVC, AD101, and TAK779 bind CCR5 differently and may act through different mechanisms [35, 50, 51, 58–62], these data suggest that the envelopes which utilized X4 efficiently maintained a conventional interaction with CCR5, rather than shifting their binding to a different part of the receptor or increasing affinity.

Considering that all variants isolated from this subject had identical V3 loops, genotypically predicted to use X4, we speculate that they represent a lineage of V3 sequences that are in fact highly adapted to using both co-receptors, but for which the polymorphism at 123 and the loss of the PNG at position 262 selectively compromise X4 binding. It is also possible, since we used assays with productive infection as their endpoint, that the described polymorphisms do not limit X4-binding *per se*, but instead compromise down-stream conformational changes which ultimately lead to more abortive entry events upon X4 binding, though we consider this alternative hypothesis unlikely.

In summary, we have isolated and described a series of closely related, naturally occurring clonal envelope variants with identical V3 loops that can utilize both CCR5 and CXCR4 efficiently. These variants have two molecular switches, a threonine at position 123 and an N-glycan at position 262, that act cooperatively to permit or constrain X4 utilization without affecting CCR5 usage. This study is, to the best of our knowledge, the first published evidence that these two sites have a direct and significant influence on co-receptor utilization by HIV-1 envelope.

## Accession Numbers

Nucleotide sequences associated with this manuscript have been submitted to GenBank with accession numbers: KP693359–KP693388.

## Supporting Information

S1 Fig Inhibition curves from single assays showing the concentration of the specified inhibitor on the x-axis and the percent infectivity on the y-axis. The legend lists the tested clone. Mutant sequences are identified by the parental clone (i.e. 542) and the residues present at positions 123 and 264 (i.e. 543 I+G indicates a mutant derived from parental clone 543 with an I at position 123 and a G at position 264). MRV = maraviroc, TAK-779, AD101 = SCH-350581, T20 = Fuzeon, nM = nanomolar.
(PDF)

S2 Fig Inhibition curves from single assays showing the concentration of the specified lectin on the x-axis and the percent infectivity on the y-axis. The legend lists the tested clone. Mutant sequences are identified by the parental clone (i.e. 542) and the residues present at positions 123 and 264 (i.e. 543 I+G indicates a mutant derived from parental clone 543 with an I at position 123 and a G at position 264). GNA = *Galanthus nivalis* agglutinin, HHA = *Hippeastrum* hybrid (Amaryllis) agglutinin, UDA = *Urtica dioica* agglutinin, ug/ml = micrograms per milliliter.
(PDF)

## References

[1] Wilen CB, Tilton JC, Doms RW. HIV: cell binding and entry. Cold Spring Harb Perspect Med. 2012;2(8). Epub 2012/08/22. 10.1101/cshperspect.a006866 . 22908191 PMC3405824

[2] Keele BF, Giorgi EE, Salazar-Gonzalez JF, Decker JM, Pham KT, Salazar MG, et al Identification and characterization of transmitted and early founder virus envelopes in primary HIV-1 infection. Proceedings of the National Academy of Sciences of the United States of America. 2008;105(21):7552–7. Epub 2008/05/21. 10.1073/pnas.0802203105 18490657 PMC2387184

[3] Grivel JC, Shattock RJ, Margolis LB. Selective transmission of R5 HIV-1 variants: where is the gatekeeper? J Transl Med. 2011;9 Suppl 1:S6 Epub 2011/02/10. 10.1186/1479-5876-9-S1-S6 21284905 PMC3105506

[4] Huang Y, Paxton WA, Wolinsky SM, Neumann AU, Zhang L, He T, et al The role of a mutant CCR5 allele in HIV-1 transmission and disease progression. Nat Med. 1996;2(11):1240–3. Epub 1996/11/01. . 8898752 10.1038/nm1196-1240

[5] Liu R, Paxton WA, Choe S, Ceradini D, Martin SR, Horuk R, et al Homozygous defect in HIV-1 coreceptor accounts for resistance of some multiply-exposed individuals to HIV-1 infection. Cell. 1996;86(3):367–77. Epub 1996/08/09. . 8756719 10.1016/s0092-8674(00)80110-5

[6] Allers K, Hutter G, Hofmann J, Loddenkemper C, Rieger K, Thiel E, et al Evidence for the cure of HIV infection by CCR5Delta32/Delta32 stem cell transplantation. Blood. 2011;117(10):2791–9. Epub 2010/12/15. 10.1182/blood-2010-09-309591 . 21148083

[7] Holt N, Wang J, Kim K, Friedman G, Wang X, Taupin V, et al Human hematopoietic stem/progenitor cells modified by zinc-finger nucleases targeted to CCR5 control HIV-1 in vivo. Nat Biotechnol. 2010;28(8):839–47. Epub 2010/07/06. 10.1038/nbt.1663 20601939 PMC3080757

[8] Hutter G, Nowak D, Mossner M, Ganepola S, Mussig A, Allers K, et al Long-term control of HIV by CCR5 Delta32/Delta32 stem-cell transplantation. N Engl J Med. 2009;360(7):692–8. Epub 2009/02/14. 10.1056/NEJMoa0802905 . 19213682

[9] Neff CP, Ndolo T, Tandon A, Habu Y, Akkina R. Oral pre-exposure prophylaxis by anti-retrovirals raltegravir and maraviroc protects against HIV-1 vaginal transmission in a humanized mouse model. PLoS One. 2010;5(12):e15257 Epub 2011/01/05. 10.1371/journal.pone.0015257 21203568 PMC3006206

[10] Hoxie JA, June CH. Novel cell and gene therapies for HIV. Cold Spring Harb Perspect Med. 2012;2(10). Epub 2012/10/03. 10.1101/cshperspect.a007179 . 23028130 PMC3475401

[11] Cooper DA, Heera J, Goodrich J, Tawadrous M, Saag M, Dejesus E, et al Maraviroc versus efavirenz, both in combination with zidovudine-lamivudine, for the treatment of antiretroviral-naive subjects with CCR5-tropic HIV-1 infection. J Infect Dis. 2010;201(6):803–13. Epub 2010/02/16. 10.1086/650697 . 20151839

[12] Su Z, Gulick RM, Krambrink A, Coakley E, Hughes MD, Han D, et al Response to vicriviroc in treatment-experienced subjects, as determined by an enhanced-sensitivity coreceptor tropism assay: reanalysis of AIDS clinical trials group A5211. J Infect Dis. 2009;200(11):1724–8. Epub 2009/10/31. 10.1086/648090 19874179 PMC2783913

[13] Westby M, Lewis M, Whitcomb J, Youle M, Pozniak AL, James IT, et al Emergence of CXCR4-using human immunodeficiency virus type 1 (HIV-1) variants in a minority of HIV-1-infected patients following treatment with the CCR5 antagonist maraviroc is from a pretreatment CXCR4-using virus reservoir. Journal of virology. 2006;80(10):4909–20. Epub 2006/04/28. 10.1128/JVI.80.10.4909-4920.2006 16641282 PMC1472081

[14] Huang CC, Lam SN, Acharya P, Tang M, Xiang SH, Hussan SS, et al Structures of the CCR5 N terminus and of a tyrosine-sulfated antibody with HIV-1 gp120 and CD4. Science. 2007;317(5846):1930–4. Epub 2007/09/29. 10.1126/science.1145373 17901336 PMC2278242

[15] Cormier EG, Dragic T. The crown and stem of the V3 loop play distinct roles in human immunodeficiency virus type 1 envelope glycoprotein interactions with the CCR5 coreceptor. Journal of virology. 2002;76(17):8953–7. Epub 2002/08/07. 12163614 10.1128/JVI.76.17.8953-8957.2002 PMC136967

[16] Schnur E, Noah E, Ayzenshtat I, Sargsyan H, Inui T, Ding FX, et al The conformation and orientation of a 27-residue CCR5 peptide in a ternary complex with HIV-1 gp120 and a CD4-mimic peptide. J Mol Biol. 2011;410(5):778–97. Epub 2011/07/19. 10.1016/j.jmb.2011.04.023 21763489 PMC3139148

[17] Jensen MA, Li FS, van 't Wout AB, Nickle DC, Shriner D, He HX, et al Improved coreceptor usage prediction and genotypic monitoring of R5-to-X4 transition by motif analysis of human immunodeficiency virus type 1 env V3 loop sequences. Journal of virology. 2003;77(24):13376–88. Epub 2003/12/03. 14645592 10.1128/JVI.77.24.13376-13388.2003 PMC296044

[18] Fouchier RA, Groenink M, Kootstra NA, Tersmette M, Huisman HG, Miedema F, et al Phenotype-associated sequence variation in the third variable domain of the human immunodeficiency virus type 1 gp120 molecule. Journal of virology. 1992;66(5):3183–7. Epub 1992/05/01. 1560543 10.1128/jvi.66.5.3183-3187.1992 PMC241084

[19] Ghaffari G, Tuttle DL, Briggs D, Burkhardt BR, Bhatt D, Andiman WA, et al Complex determinants in human immunodeficiency virus type 1 envelope gp120 mediate CXCR4-dependent infection of macrophages. Journal of virology. 2005;79(21):13250–61. Epub 2005/10/18. 10.1128/JVI.79.21.13250-13261.2005 16227248 PMC1262568

[20] Cashin K, Roche M, Sterjovski J, Ellett A, Gray LR, Cunningham AL, et al Alternative coreceptor requirements for efficient CCR5- and CXCR4-mediated HIV-1 entry into macrophages. Journal of virology. 2011;85(20):10699–709. Epub 2011/08/13. 10.1128/JVI.05510-11 21835796 PMC3187472

[21] Sanchez V, Masia M, Robledano C, Padilla S, Ramos JM, Gutierrez F. Performance of genotypic algorithms for predicting HIV-1 tropism measured against the enhanced-sensitivity Trofile coreceptor tropism assay. J Clin Microbiol. 2010;48(11):4135–9. Epub 2010/09/24. 10.1128/JCM.01204-10 20861336 PMC3020874

[22] Boyd MT, Simpson GR, Cann AJ, Johnson MA, Weiss RA. A single amino acid substitution in the V1 loop of human immunodeficiency virus type 1 gp120 alters cellular tropism. Journal of virology. 1993;67(6):3649–52. Epub 1993/06/01. 8497073 10.1128/jvi.67.6.3649-3652.1993 PMC237718

[23] Hoffman NG, Seillier-Moiseiwitsch F, Ahn J, Walker JM, Swanstrom R. Variability in the human immunodeficiency virus type 1 gp120 Env protein linked to phenotype-associated changes in the V3 loop. Journal of virology. 2002;76(8):3852–64. Epub 2002/03/22. 11907225 10.1128/JVI.76.8.3852-3864.2002 PMC136063

[24] Huang W, Toma J, Fransen S, Stawiski E, Reeves JD, Whitcomb JM, et al Coreceptor tropism can be influenced by amino acid substitutions in the gp41 transmembrane subunit of human immunodeficiency virus type 1 envelope protein. Journal of virology. 2008;82(11):5584–93. Epub 2008/03/21. 10.1128/JVI.02676-07 18353956 PMC2395220

[25] Pastore C, Nedellec R, Ramos A, Pontow S, Ratner L, Mosier DE. Human immunodeficiency virus type 1 coreceptor switching: V1/V2 gain-of-fitness mutations compensate for V3 loss-of-fitness mutations. Journal of virology. 2006;80(2):750–8. Epub 2005/12/28. 10.1128/JVI.80.2.750-758.2006 16378977 PMC1346864

[26] Thielen A, Lengauer T, Swenson LC, Dong WW, McGovern RA, Lewis M, et al Mutations in gp41 are correlated with coreceptor tropism but do not improve prediction methods substantially. Antivir Ther. 2011;16(3):319–28. Epub 2011/05/11. 10.3851/IMP1769 . 21555814

[27] Thielen A, Sichtig N, Kaiser R, Lam J, Harrigan PR, Lengauer T. Improved prediction of HIV-1 coreceptor usage with sequence information from the second hypervariable loop of gp120. J Infect Dis. 2010;202(9):1435–43. Epub 2010/09/30. 10.1086/656600 . 20874088

[28] Kiser JJ, Rutstein RM, Samson P, Graham B, Aldrovandi G, Mofenson LM, et al Atazanavir and atazanavir/ritonavir pharmacokinetics in HIV-infected infants, children, and adolescents. AIDS. 2011;25(12):1489–96. Epub 2011/05/26. 10.1097/QAD.0b013e328348fc41 21610486 PMC3177533

[29] Platt EJ, Bilska M, Kozak SL, Kabat D, Montefiori DC. Evidence that ecotropic murine leukemia virus contamination in TZM-bl cells does not affect the outcome of neutralizing antibody assays with human immunodeficiency virus type 1. Journal of virology. 2009;83(16):8289–92. Epub 2009/05/29. 10.1128/JVI.00709-09 19474095 PMC2715758

[30] Takeuchi Y, McClure MO, Pizzato M. Identification of gammaretroviruses constitutively released from cell lines used for human immunodeficiency virus research. Journal of virology. 2008;82(24):12585–8. Epub 2008/10/10. 10.1128/JVI.01726-08 18842727 PMC2593302

[31] Wei X, Decker JM, Liu H, Zhang Z, Arani RB, Kilby JM, et al Emergence of resistant human immunodeficiency virus type 1 in patients receiving fusion inhibitor (T-20) monotherapy. Antimicrob Agents Chemother. 2002;46(6):1896–905. Epub 2002/05/23. 12019106 10.1128/AAC.46.6.1896-1905.2002 PMC127242

[32] Derdeyn CA, Decker JM, Sfakianos JN, Wu X, O'Brien WA, Ratner L, et al Sensitivity of human immunodeficiency virus type 1 to the fusion inhibitor T-20 is modulated by coreceptor specificity defined by the V3 loop of gp120. Journal of virology. 2000;74(18):8358–67. Epub 2000/08/23. 10954535 10.1128/jvi.74.18.8358-8367.2000 PMC116346

[33] Platt EJ, Wehrly K, Kuhmann SE, Chesebro B, Kabat D. Effects of CCR5 and CD4 cell surface concentrations on infections by macrophagetropic isolates of human immunodeficiency virus type 1. Journal of virology. 1998;72(4):2855–64. Epub 1998/04/03. 9525605 10.1128/jvi.72.4.2855-2864.1998 PMC109730

[34] Morner A, Bjorndal A, Albert J, Kewalramani VN, Littman DR, Inoue R, et al Primary human immunodeficiency virus type 2 (HIV-2) isolates, like HIV-1 isolates, frequently use CCR5 but show promiscuity in coreceptor usage. Journal of virology. 1999;73(3):2343–9. Epub 1999/02/11. 9971817 10.1128/jvi.73.3.2343-2349.1999 PMC104479

[35] Baba M, Nishimura O, Kanzaki N, Okamoto M, Sawada H, Iizawa Y, et al A small-molecule, nonpeptide CCR5 antagonist with highly potent and selective anti-HIV-1 activity. Proceedings of the National Academy of Sciences of the United States of America. 1999;96(10):5698–703. Epub 1999/05/13. 10318947 10.1073/pnas.96.10.5698 PMC21923

[36] Hendrix CW, Flexner C, MacFarland RT, Giandomenico C, Fuchs EJ, Redpath E, et al Pharmacokinetics and safety of AMD-3100, a novel antagonist of the CXCR-4 chemokine receptor, in human volunteers. Antimicrob Agents Chemother. 2000;44(6):1667–73. Epub 2000/05/19. 10817726 10.1128/aac.44.6.1667-1673.2000 PMC89930

[37] Bridger GJ, Skerlj RT, Thornton D, Padmanabhan S, Martellucci SA, Henson GW, et al Synthesis and structure-activity relationships of phenylenebis(methylene)-linked bis-tetraazamacrocycles that inhibit HIV replication. Effects of macrocyclic ring size and substituents on the aromatic linker. J Med Chem. 1995;38(2):366–78. Epub 1995/01/20. . 7830280 10.1021/jm00002a019

[38] De Clercq E, Yamamoto N, Pauwels R, Balzarini J, Witvrouw M, De Vreese K, et al Highly potent and selective inhibition of human immunodeficiency virus by the bicyclam derivative JM3100. Antimicrob Agents Chemother. 1994;38(4):668–74. Epub 1994/04/01. 7913308 10.1128/aac.38.4.668 PMC284523

[39] Salazar-Gonzalez JF, Bailes E, Pham KT, Salazar MG, Guffey MB, Keele BF, et al Deciphering human immunodeficiency virus type 1 transmission and early envelope diversification by single-genome amplification and sequencing. Journal of virology. 2008;82(8):3952–70. Epub 2008/02/08. 10.1128/JVI.02660-07 18256145 PMC2293010

[40] Brumme ZL, Dong WW, Yip B, Wynhoven B, Hoffman NG, Swanstrom R, et al Clinical and immunological impact of HIV envelope V3 sequence variation after starting initial triple antiretroviral therapy. AIDS. 2004;18(4):F1–9. Epub 2004/04/20. . 15090786 10.1097/00002030-200403050-00001

[41] Jensen MA, Coetzer M, van 't Wout AB, Morris L, Mullins JI. A reliable phenotype predictor for human immunodeficiency virus type 1 subtype C based on envelope V3 sequences. Journal of virology. 2006;80(10):4698–704. Epub 2006/04/28. 10.1128/JVI.80.10.4698-4704.2006 16641263 PMC1472078

[42] Lengauer T, Sander O, Sierra S, Thielen A, Kaiser R. Bioinformatics prediction of HIV coreceptor usage. Nat Biotechnol. 2007;25(12):1407–10. Epub 2007/12/11. 10.1038/nbt1371 . 18066037

[43] Wei X, Decker JM, Wang S, Hui H, Kappes JC, Wu X, et al Antibody neutralization and escape by HIV-1. Nature. 2003;422(6929):307–12. Epub 2003/03/21. 10.1038/nature01470 . 12646921

[44] He J, Choe S, Walker R, Di Marzio P, Morgan DO, Landau NR. Human immunodeficiency virus type 1 viral protein R (Vpr) arrests cells in the G2 phase of the cell cycle by inhibiting p34cdc2 activity. Journal of virology. 1995;69(11):6705–11. Epub 1995/11/01. 7474080 10.1128/jvi.69.11.6705-6711.1995 PMC189580

[45] Connor RI, Chen BK, Choe S, Landau NR. Vpr is required for efficient replication of human immunodeficiency virus type-1 in mononuclear phagocytes. Virology. 1995;206(2):935–44. Epub 1995/02/01. 10.1006/viro.1995.1016 . 7531918

[46] Levy DN, Aldrovandi GM, Kutsch O, Shaw GM. Dynamics of HIV-1 recombination in its natural target cells. Proceedings of the National Academy of Sciences of the United States of America. 2004;101(12):4204–9. Epub 2004/03/11. 10.1073/pnas.0306764101 15010526 PMC384719

[47] Kutsch O, Benveniste EN, Shaw GM, Levy DN. Direct and quantitative single-cell analysis of human immunodeficiency virus type 1 reactivation from latency. Journal of virology. 2002;76(17):8776–86. Epub 2002/08/07. 12163598 10.1128/JVI.76.17.8776-8786.2002 PMC136999

[48] Nakamura KJ, Gach JS, Jones L, Semrau K, Walter J, Bibollet-Ruche F, et al 4E10-resistant HIV-1 isolated from four subjects with rare membrane-proximal external region polymorphisms. PLoS One. 2010;5(3):e9786 Epub 2010/03/31. 10.1371/journal.pone.0009786 20352106 PMC2843716

[49] Mao Y, Wang L, Gu C, Herschhorn A, Desormeaux A, Finzi A, et al Molecular architecture of the uncleaved HIV-1 envelope glycoprotein trimer. Proceedings of the National Academy of Sciences of the United States of America. 2013;110(30):12438–43. Epub 2013/06/13. 10.1073/pnas.1307382110 23757493 PMC3725050

[50] Westby M, Smith-Burchnell C, Mori J, Lewis M, Mosley M, Stockdale M, et al Reduced maximal inhibition in phenotypic susceptibility assays indicates that viral strains resistant to the CCR5 antagonist maraviroc utilize inhibitor-bound receptor for entry. Journal of virology. 2007;81(5):2359–71. Epub 2006/12/22. 10.1128/JVI.02006-06 17182681 PMC1865946

[51] Reeves JD, Gallo SA, Ahmad N, Miamidian JL, Harvey PE, Sharron M, et al Sensitivity of HIV-1 to entry inhibitors correlates with envelope/coreceptor affinity, receptor density, and fusion kinetics. Proceedings of the National Academy of Sciences of the United States of America. 2002;99(25):16249–54. Epub 2002/11/22. 10.1073/pnas.252469399 12444251 PMC138597

[52] Greenberg ML, Cammack N. Resistance to enfuvirtide, the first HIV fusion inhibitor. J Antimicrob Chemother. 2004;54(2):333–40. Epub 2004/07/03. 10.1093/jac/dkh330 . 15231762

[53] Balzarini J. Inhibition of HIV entry by carbohydrate-binding proteins. Antiviral Res. 2006;71(2–3):237–47. Epub 2006/03/30. 10.1016/j.antiviral.2006.02.004 . 16569440

[54] Go EP, Hewawasam G, Liao HX, Chen H, Ping LH, Anderson JA, et al Characterization of glycosylation profiles of HIV-1 transmitted/founder envelopes by mass spectrometry. Journal of virology. 2011;85(16):8270–84. Epub 2011/06/10. 10.1128/JVI.05053-11 21653661 PMC3147976

[55] Go EP, Liao HX, Alam SM, Hua D, Haynes BF, Desaire H. Characterization of host-cell line specific glycosylation profiles of early transmitted/founder HIV-1 gp120 envelope proteins. J Proteome Res. 2013;12(3):1223–34. Epub 2013/01/24. 10.1021/pr300870t 23339644 PMC3674872

[56] Wang W, Nie J, Prochnow C, Truong C, Jia Z, Wang S, et al A systematic study of the N-glycosylation sites of HIV-1 envelope protein on infectivity and antibody-mediated neutralization. Retrovirology. 2013;10:14 Epub 2013/02/07. 10.1186/1742-4690-10-14 23384254 PMC3648360

[57] Lee WR, Syu WJ, Du B, Matsuda M, Tan S, Wolf A, et al Nonrandom distribution of gp120 N-linked glycosylation sites important for infectivity of human immunodeficiency virus type 1. Proceedings of the National Academy of Sciences of the United States of America. 1992;89(6):2213–7. Epub 1992/03/15. 1549584 10.1073/pnas.89.6.2213 PMC48627

[58] Dragic T, Trkola A, Thompson DA, Cormier EG, Kajumo FA, Maxwell E, et al A binding pocket for a small molecule inhibitor of HIV-1 entry within the transmembrane helices of CCR5. Proceedings of the National Academy of Sciences of the United States of America. 2000;97(10):5639–44. Epub 2000/04/26. 10.1073/pnas.090576697 10779565 PMC25881

[59] Platt EJ, Durnin JP, Kabat D. Kinetic factors control efficiencies of cell entry, efficacies of entry inhibitors, and mechanisms of adaptation of human immunodeficiency virus. Journal of virology. 2005;79(7):4347–56. Epub 2005/03/16. 10.1128/JVI.79.7.4347-4356.2005 15767435 PMC1061535

[60] Tilton JC, Wilen CB, Didigu CA, Sinha R, Harrison JE, Agrawal-Gamse C, et al A maraviroc-resistant HIV-1 with narrow cross-resistance to other CCR5 antagonists depends on both N-terminal and extracellular loop domains of drug-bound CCR5. Journal of virology. 2010;84(20):10863–76. Epub 2010/08/13. 10.1128/JVI.01109-10 20702642 PMC2950574

[61] Tsamis F, Gavrilov S, Kajumo F, Seibert C, Kuhmann S, Ketas T, et al Analysis of the mechanism by which the small-molecule CCR5 antagonists SCH-351125 and SCH-350581 inhibit human immunodeficiency virus type 1 entry. Journal of virology. 2003;77(9):5201–8. Epub 2003/04/15. 12692222 10.1128/JVI.77.9.5201-5208.2003 PMC153966

[62] Garcia-Perez J, Rueda P, Alcami J, Rognan D, Arenzana-Seisdedos F, Lagane B, et al Allosteric model of maraviroc binding to CC chemokine receptor 5 (CCR5). J Biol Chem. 2011;286(38):33409–21. Epub 2011/07/22. 10.1074/jbc.M111.279596 21775441 PMC3190905

