# Supplementary figures and images for: A Conserved Glycan in the C2 Domain of HIV-1 Envelope Acts as a Molecular Switch to Control X4 Utilization by Clonal Variants with Identical V3 Loops
Source: PLoS One. 2015 Jun 17;10(6):e0128116. doi: 10.1371/journal.pone.0128116 (PMC4471078; doi:10.1371/journal.pone.0128116)

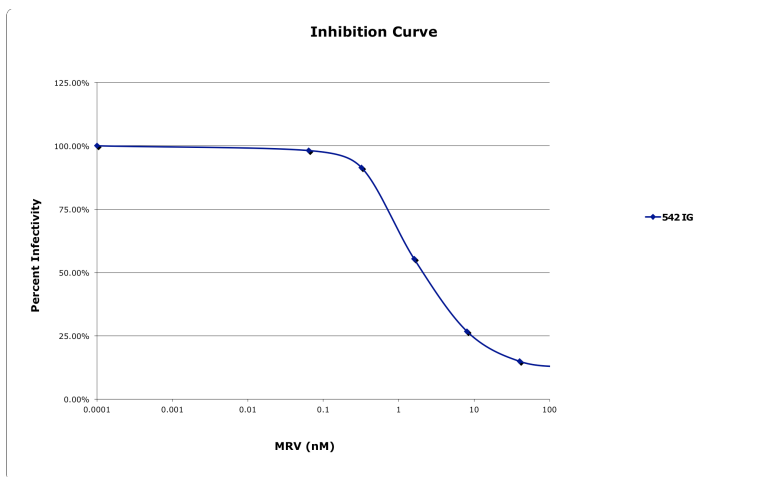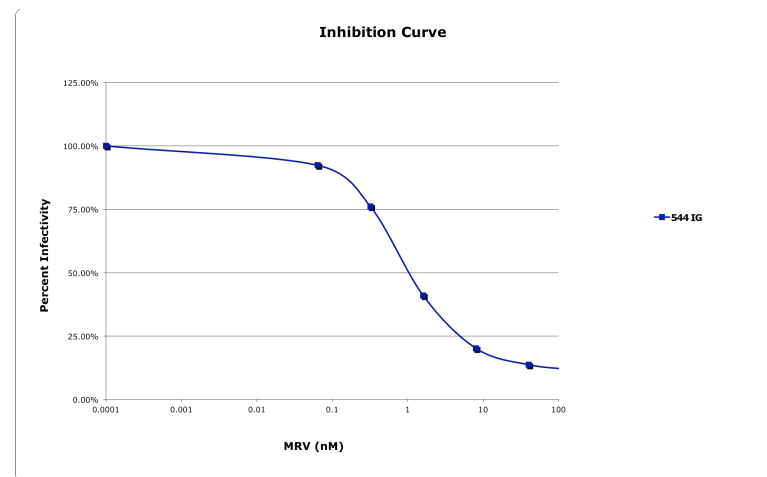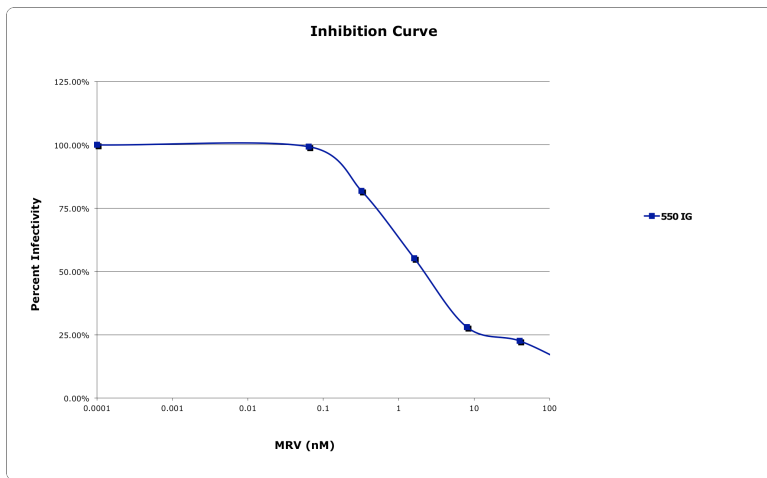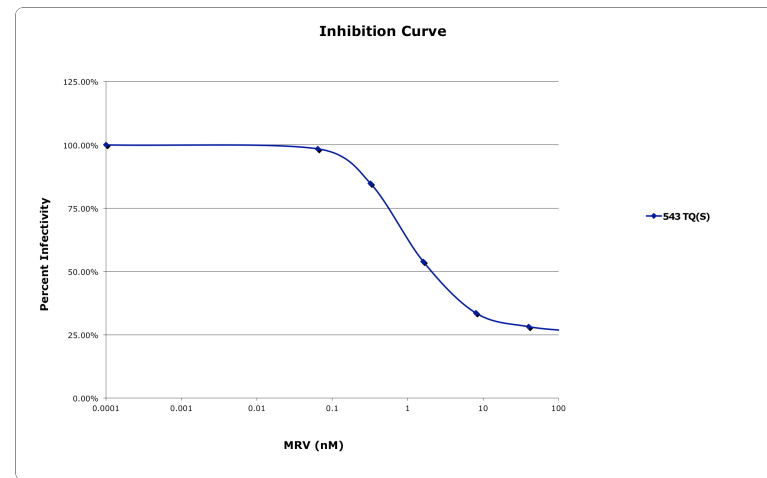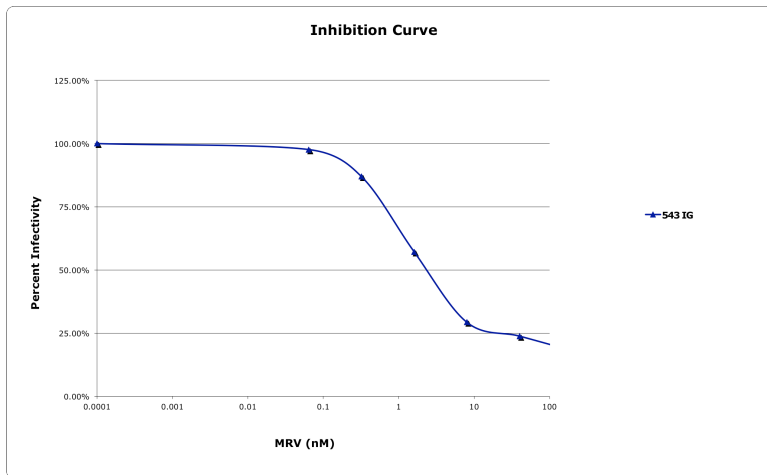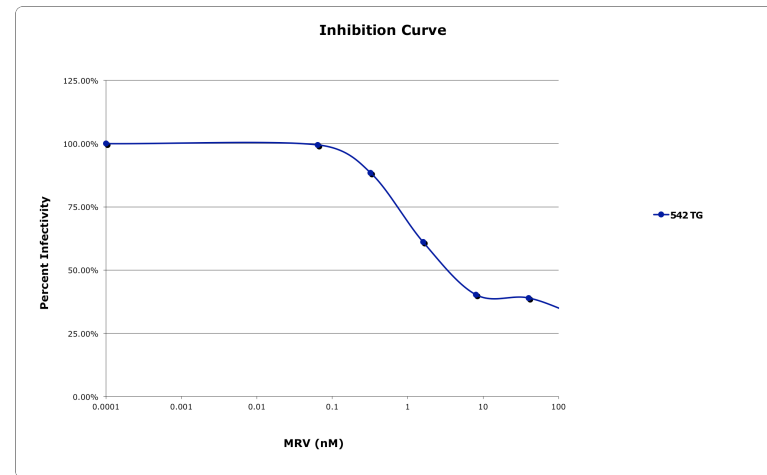

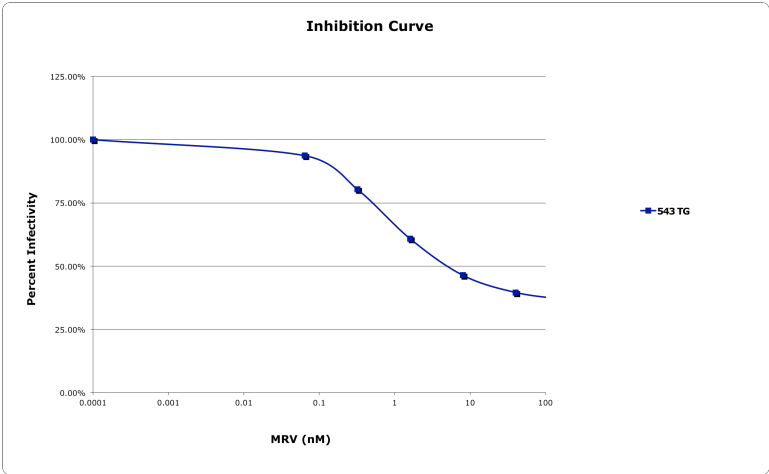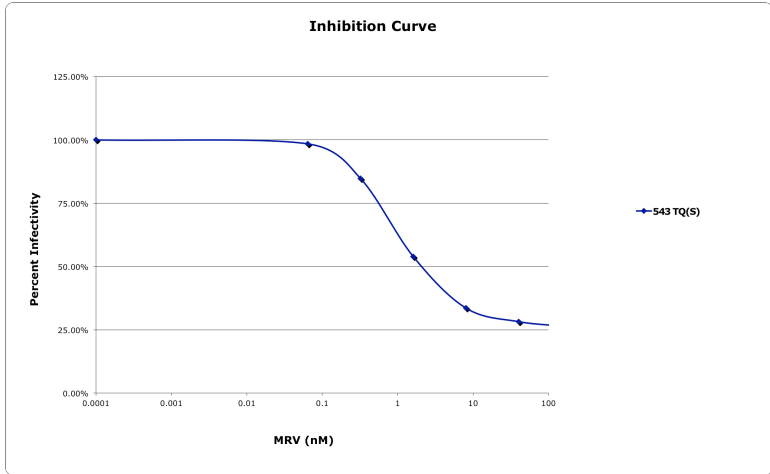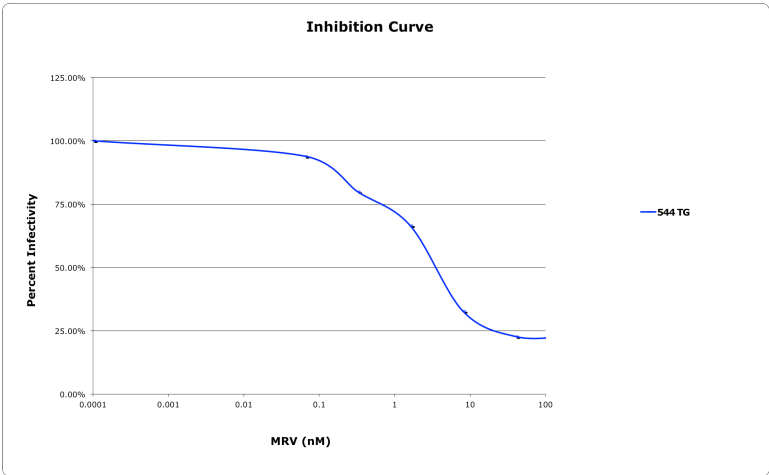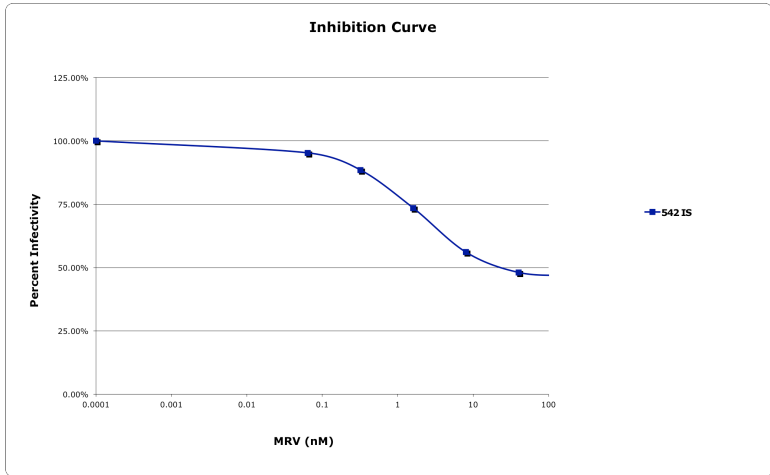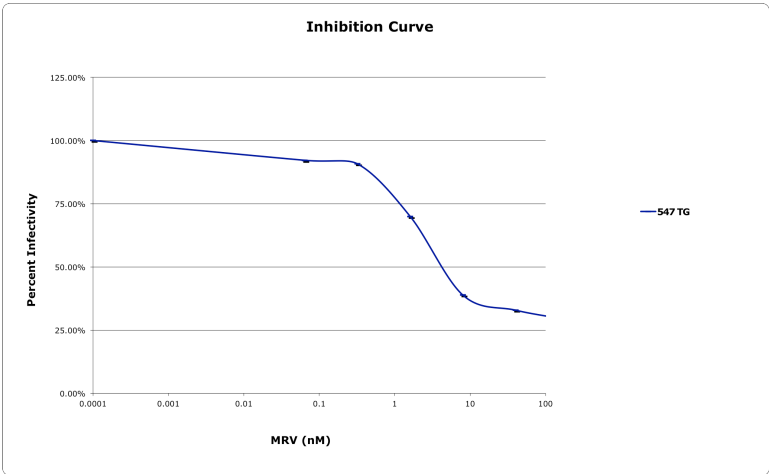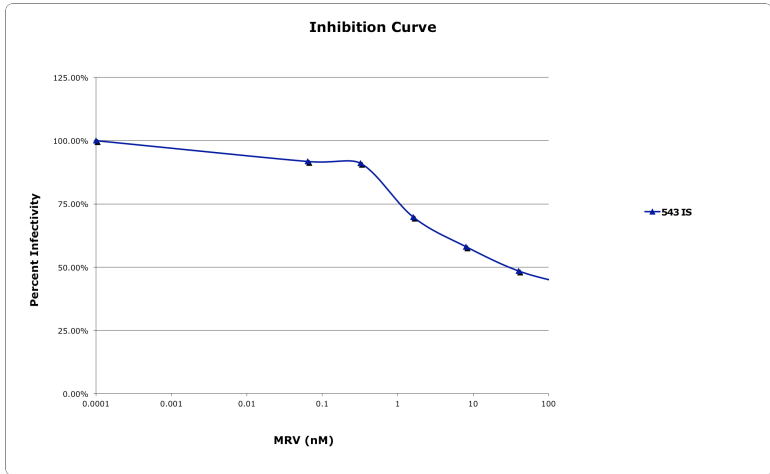

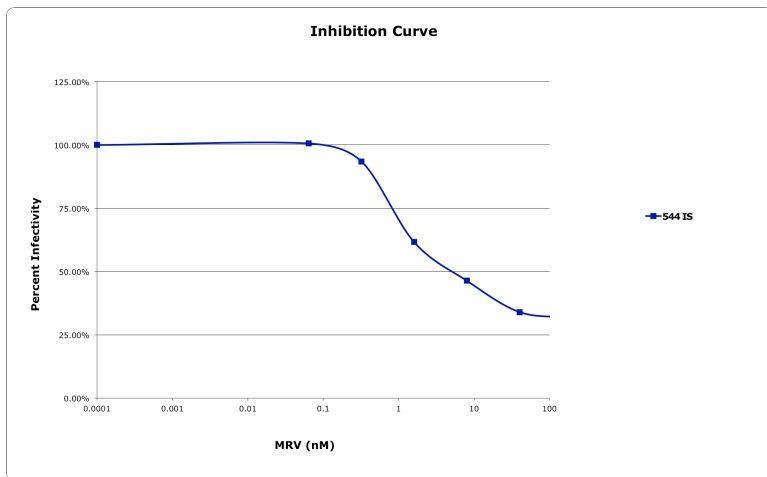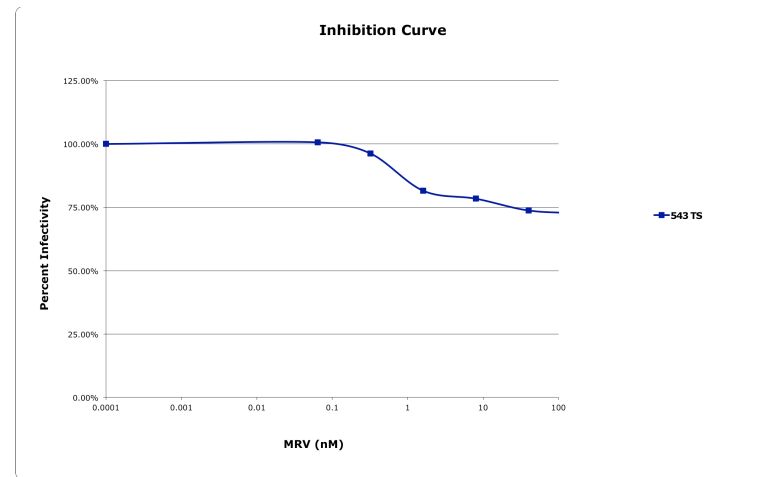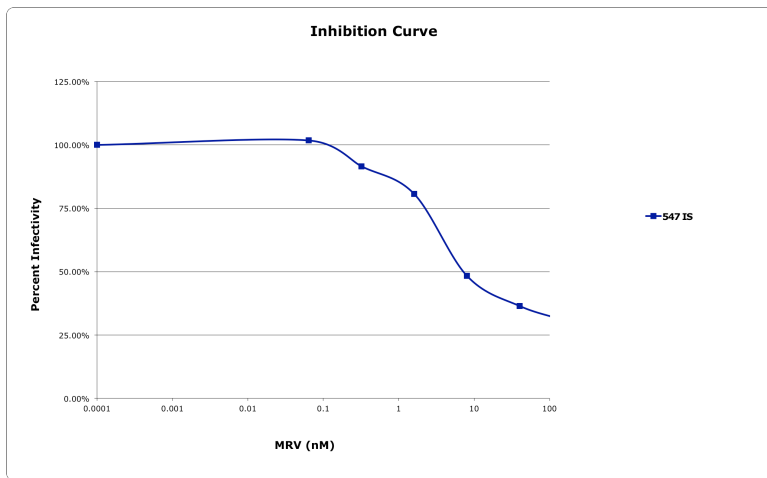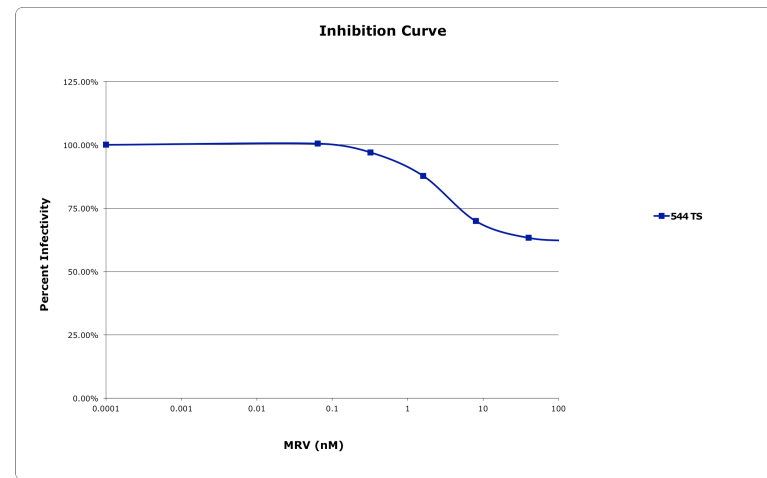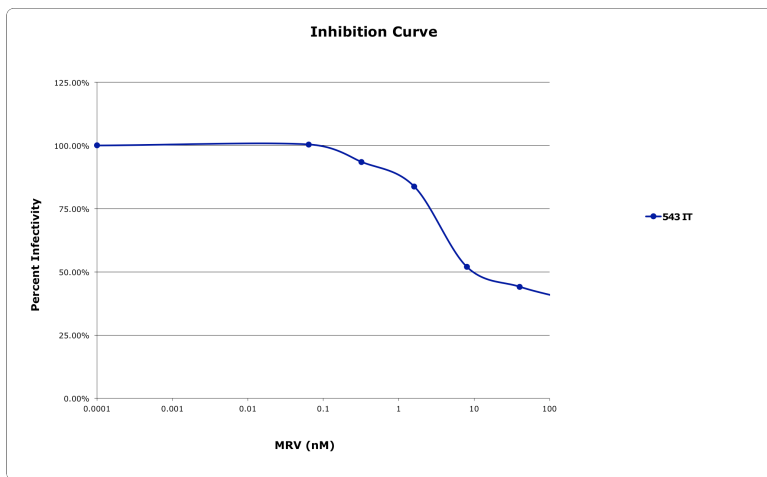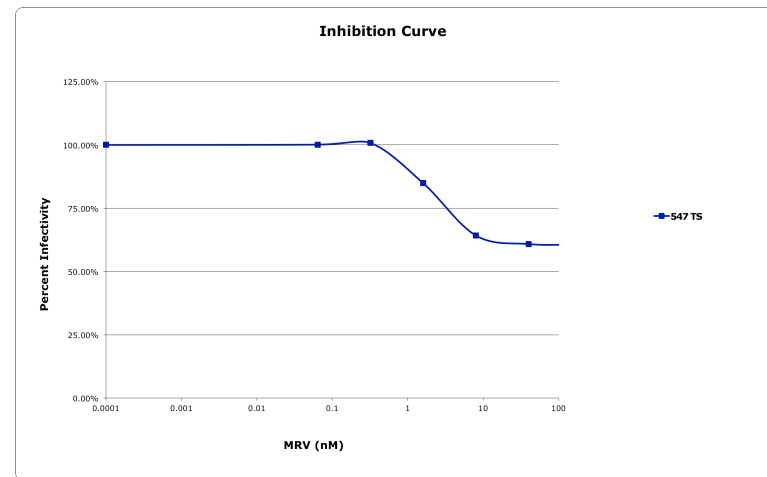

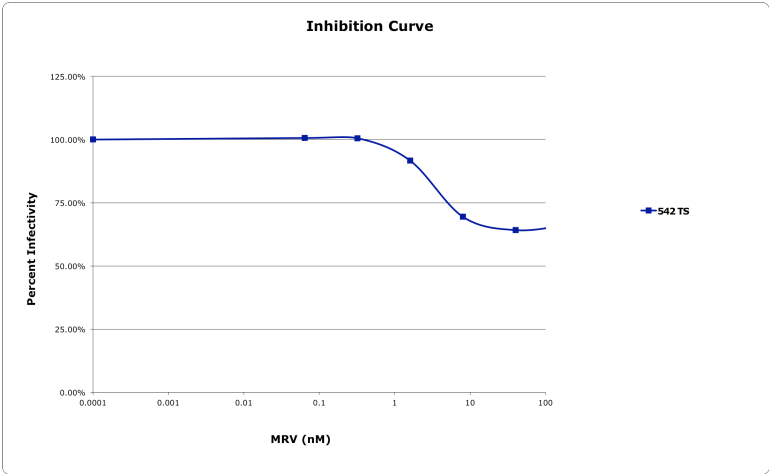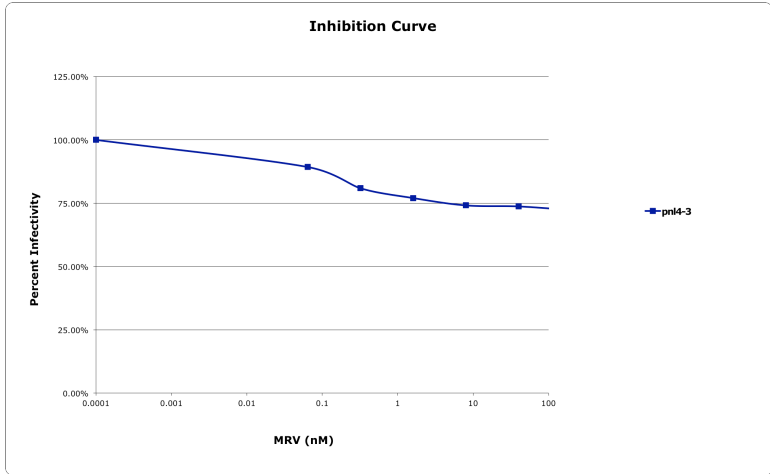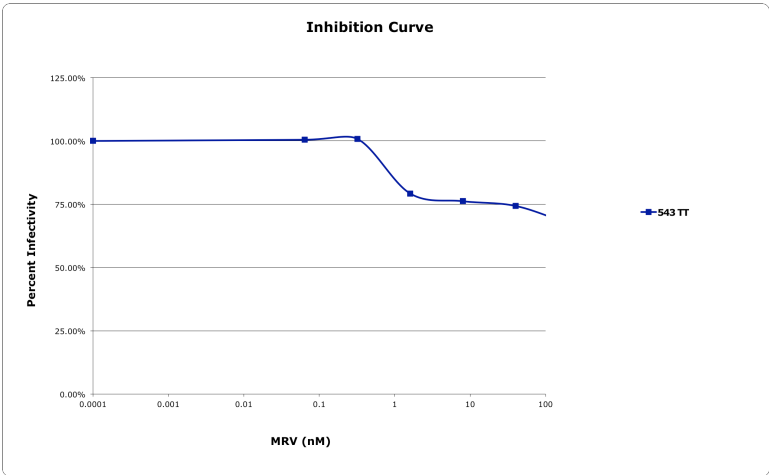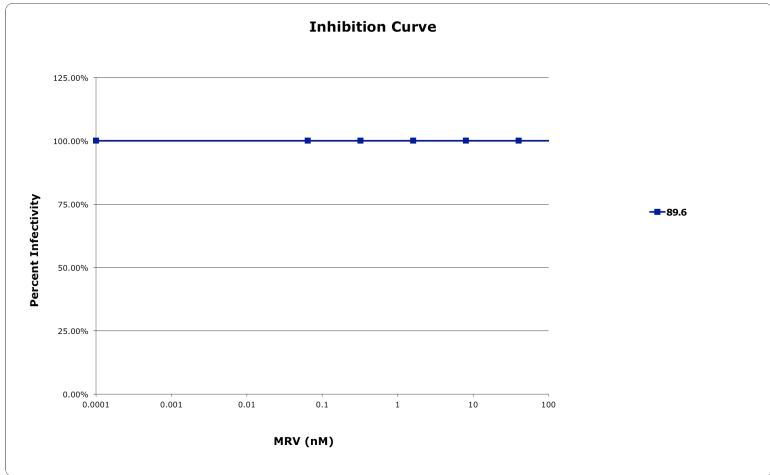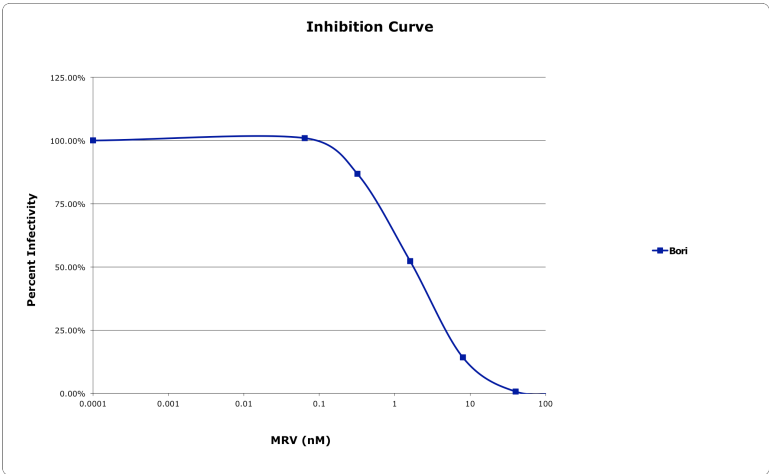

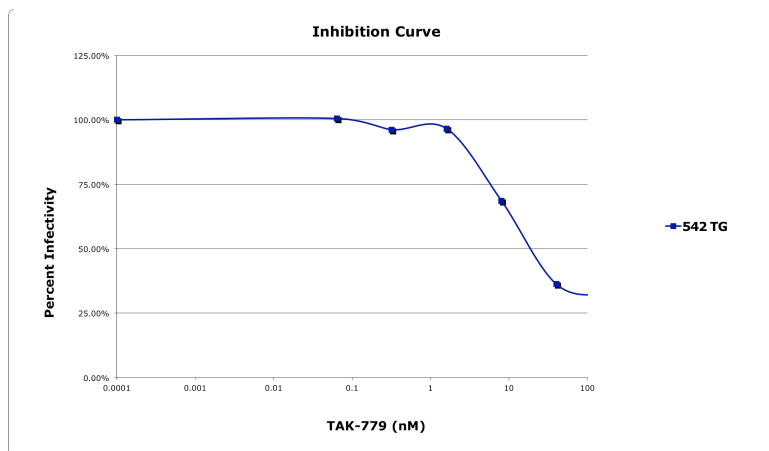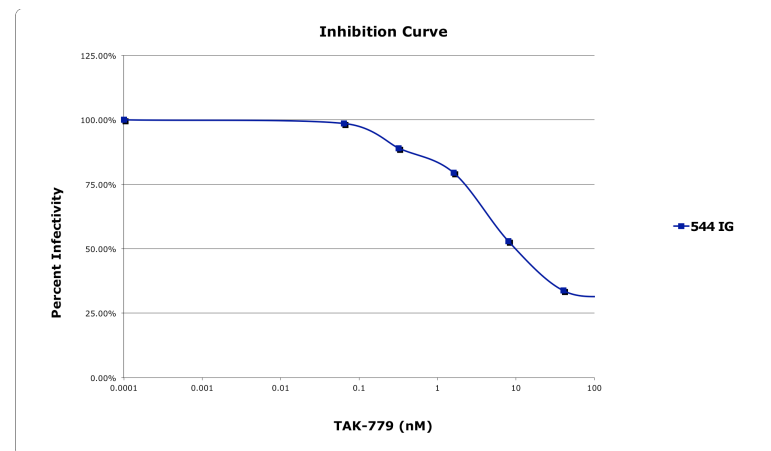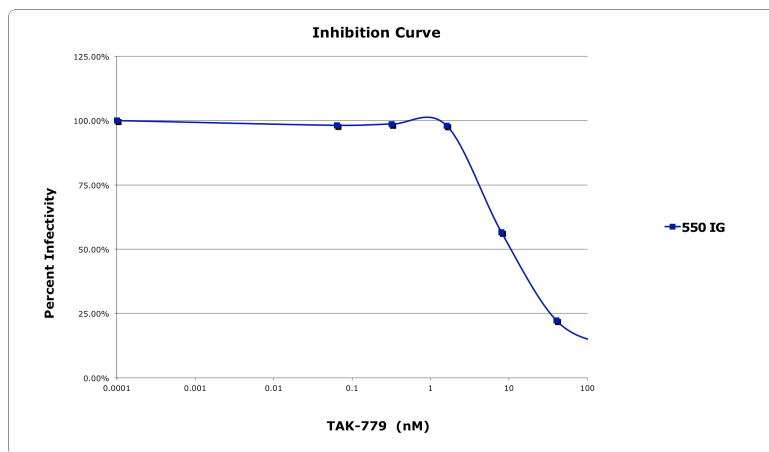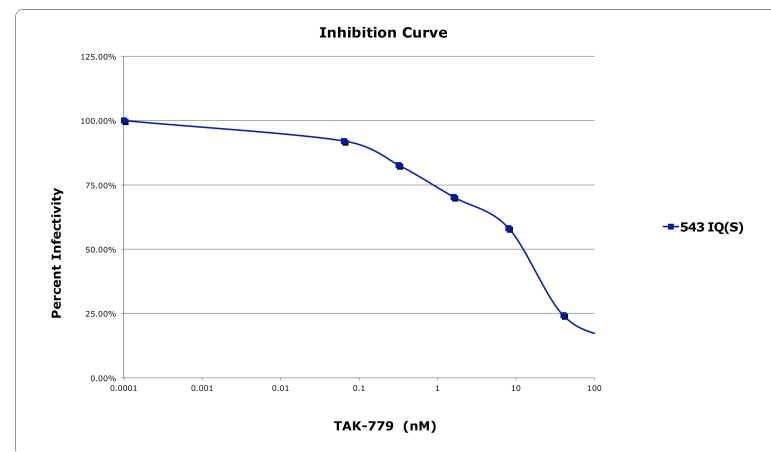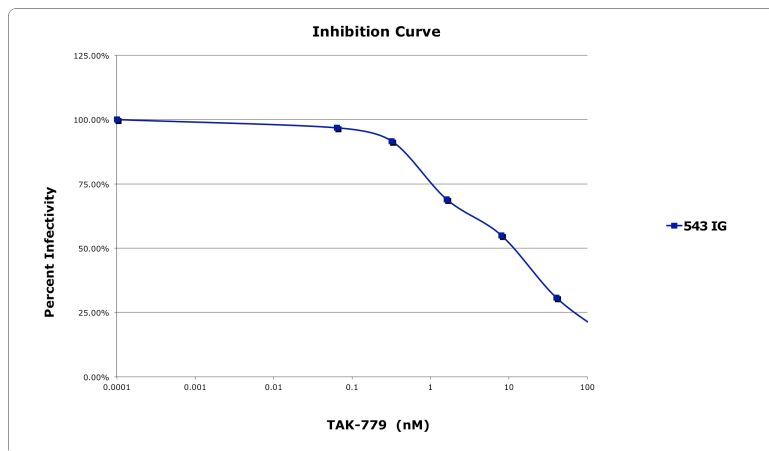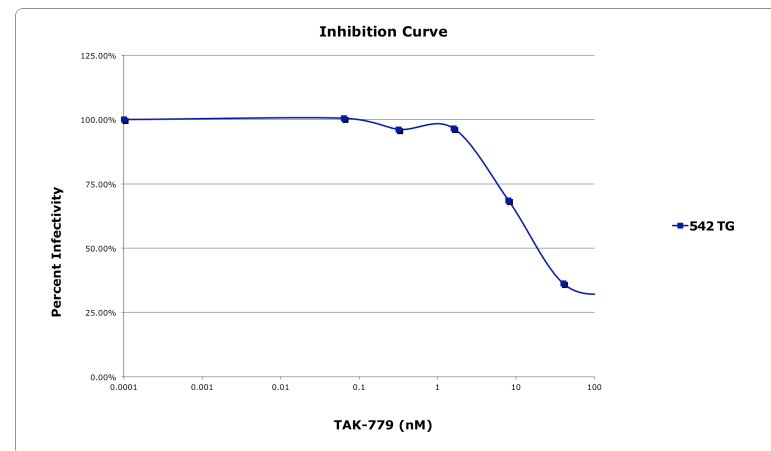

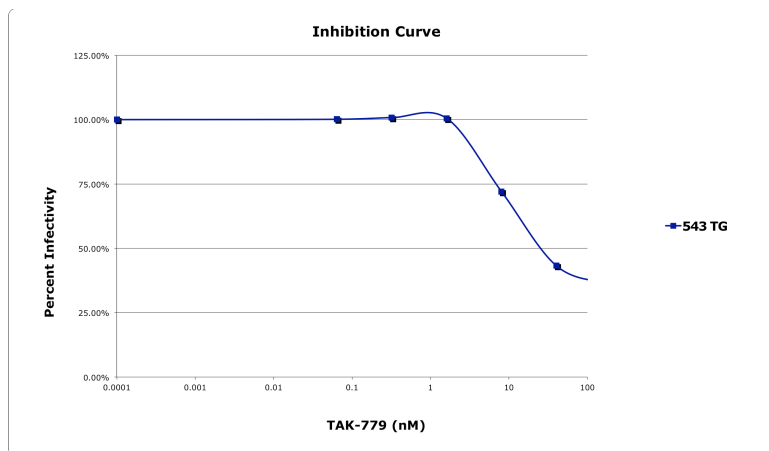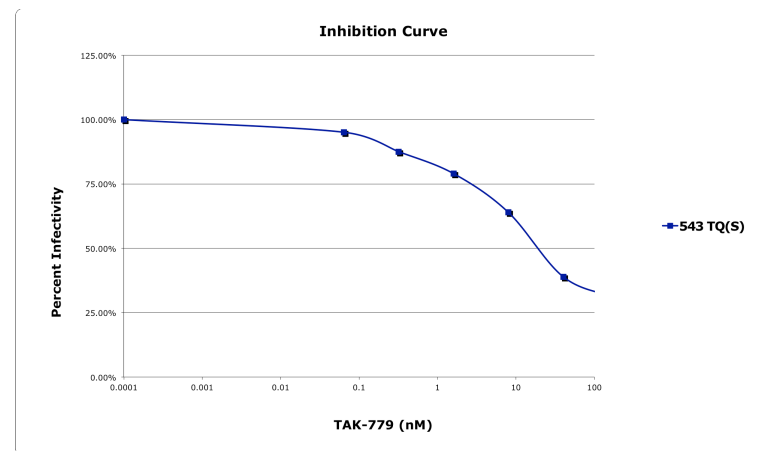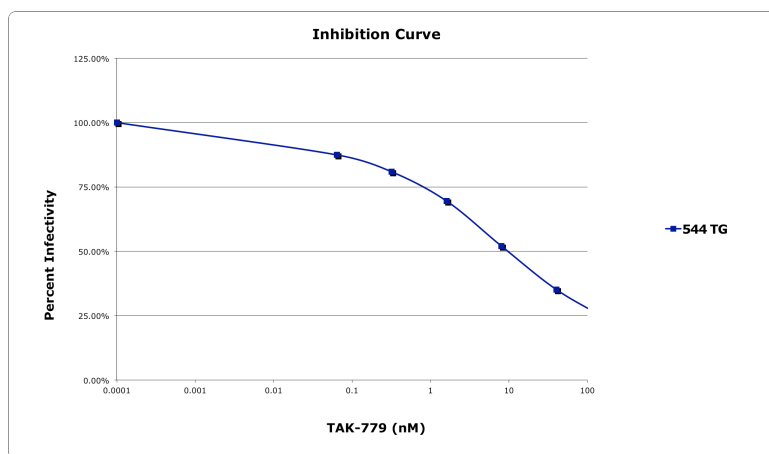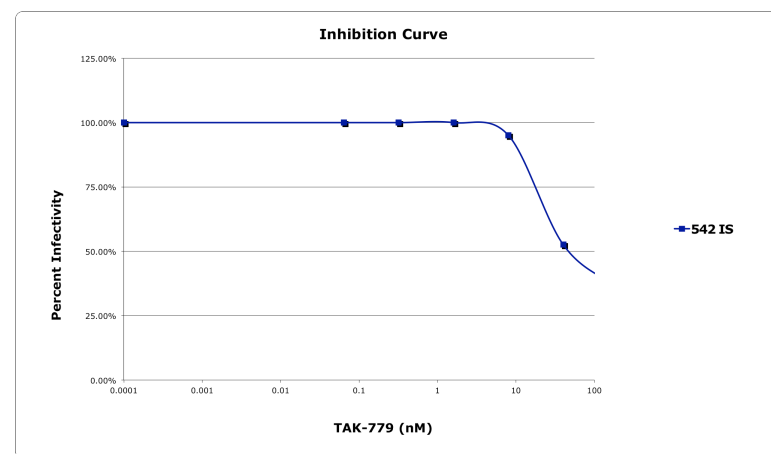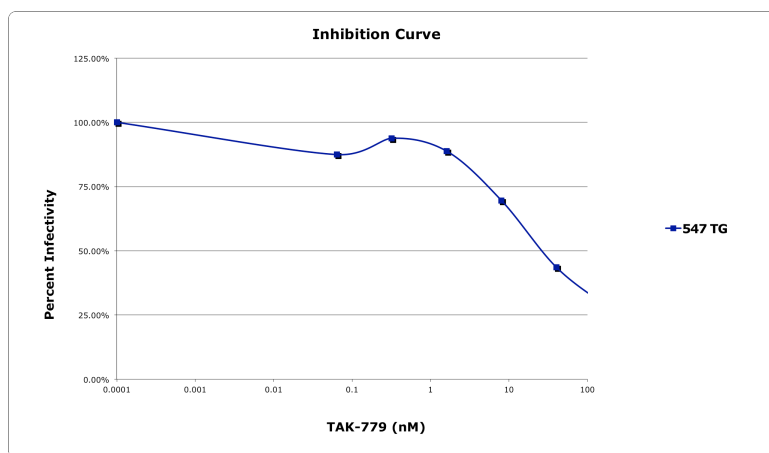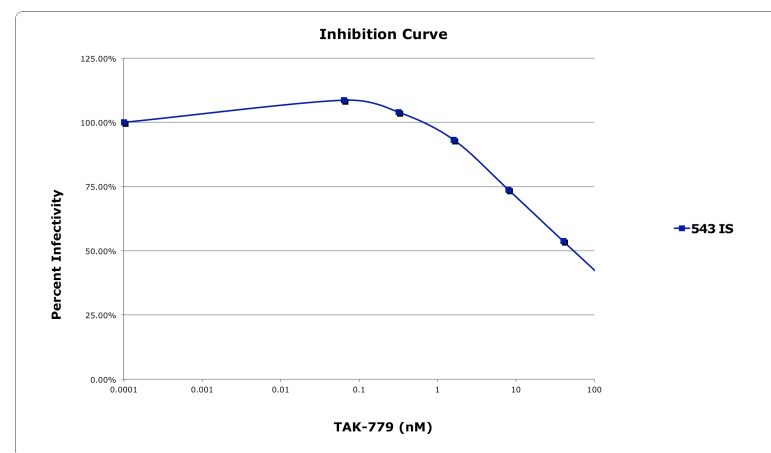

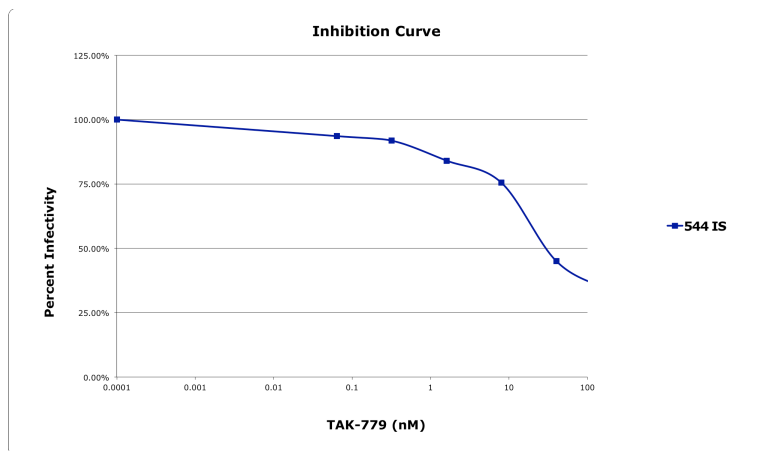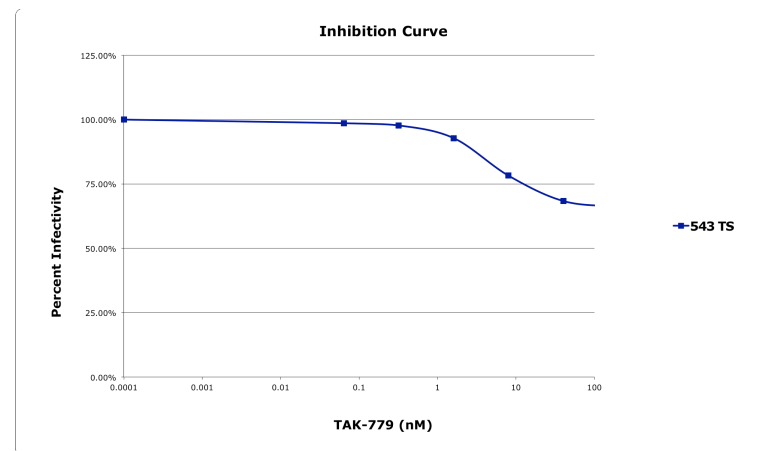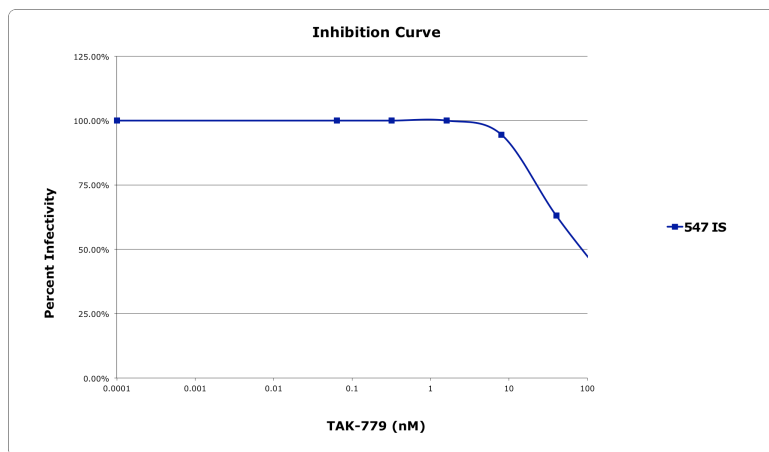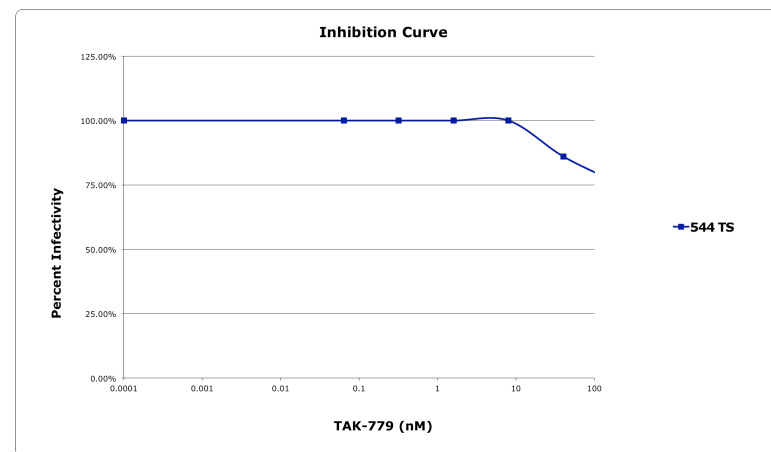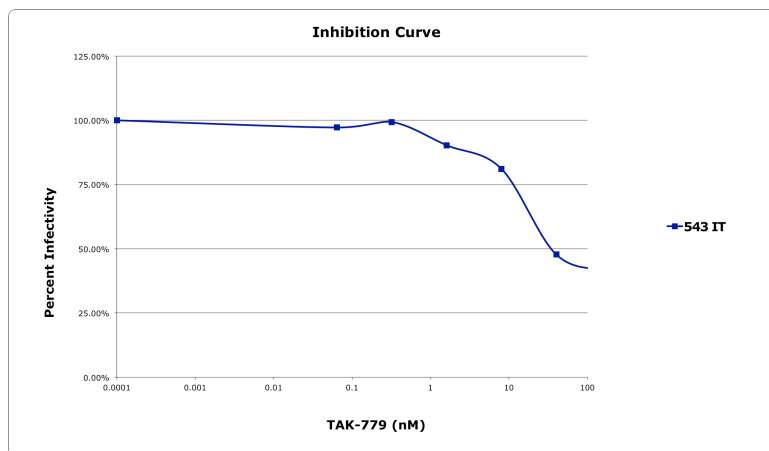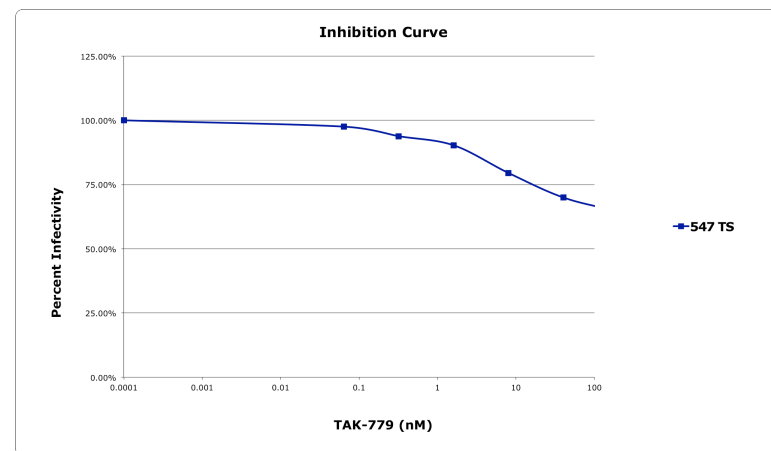

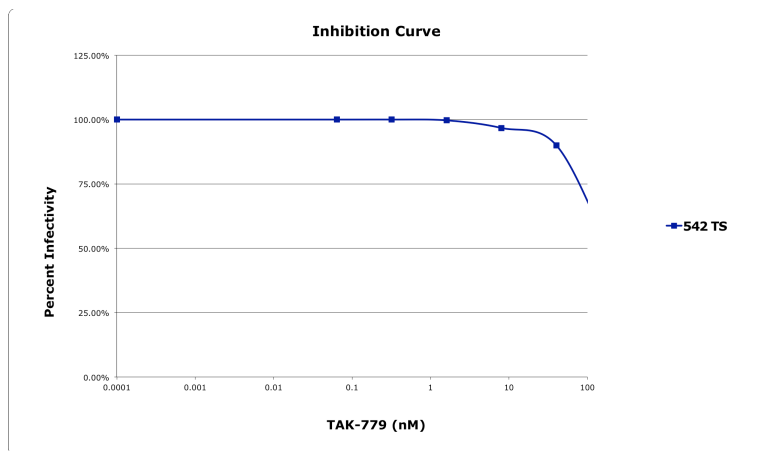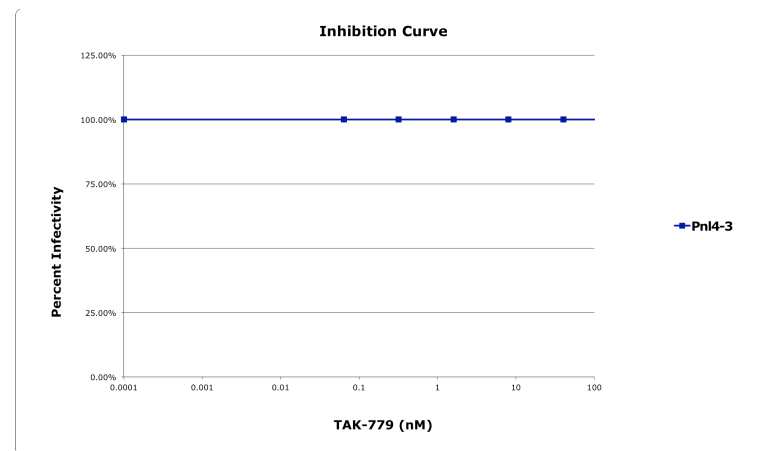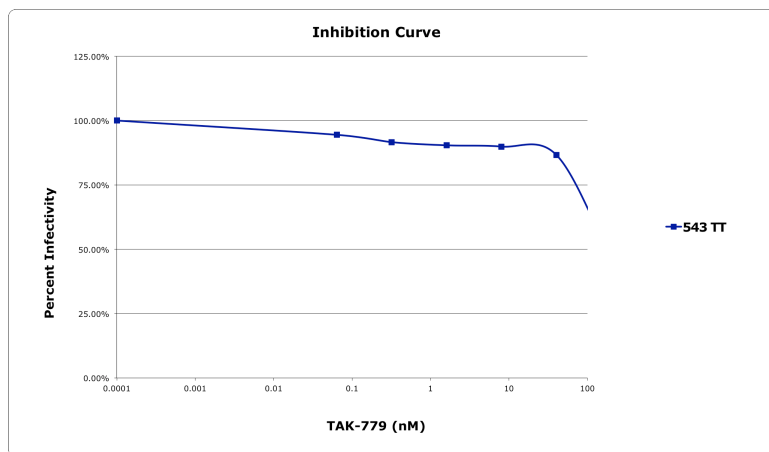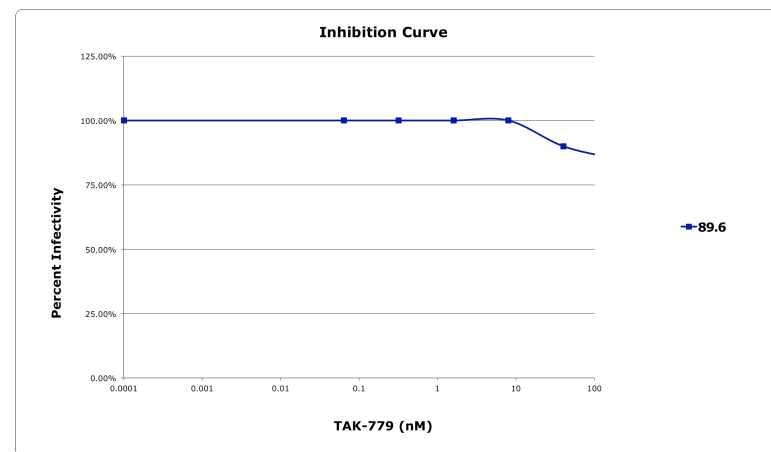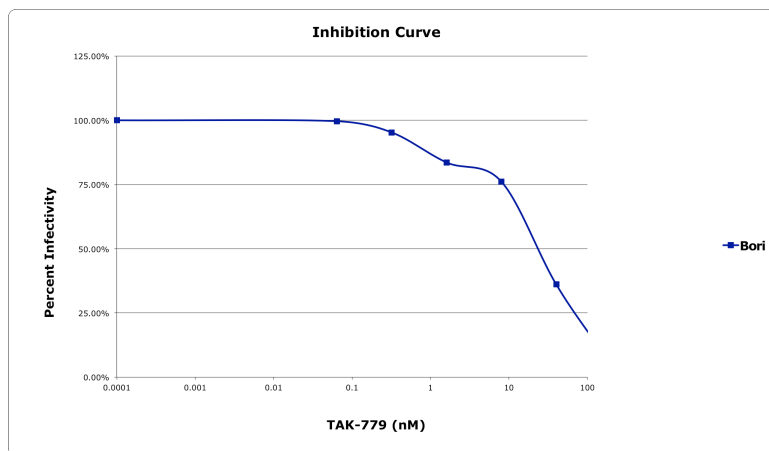

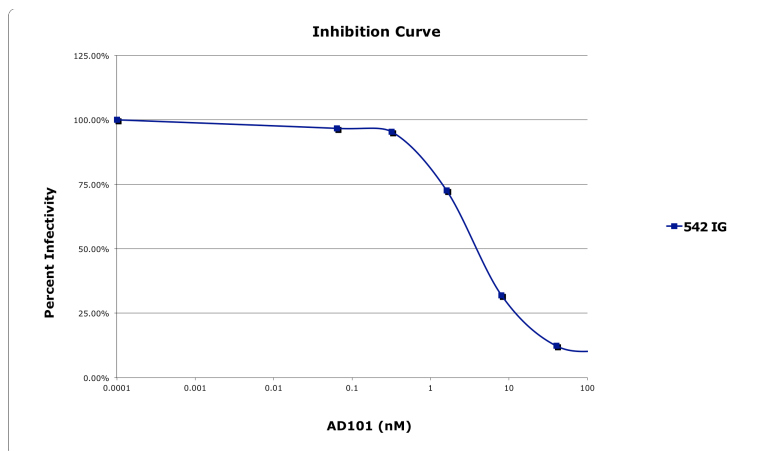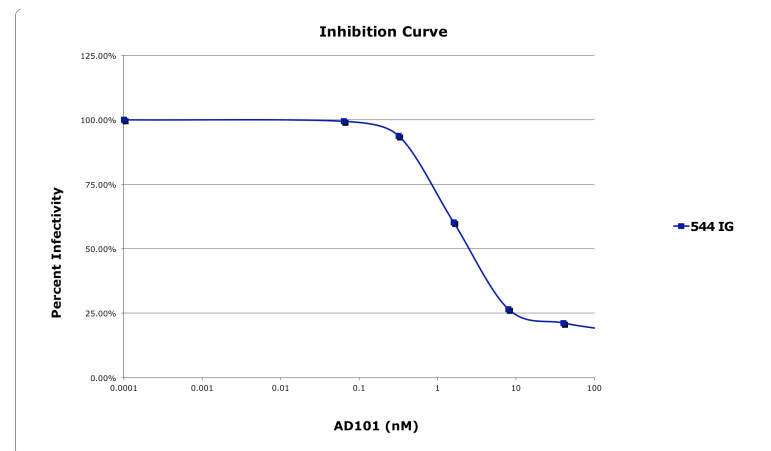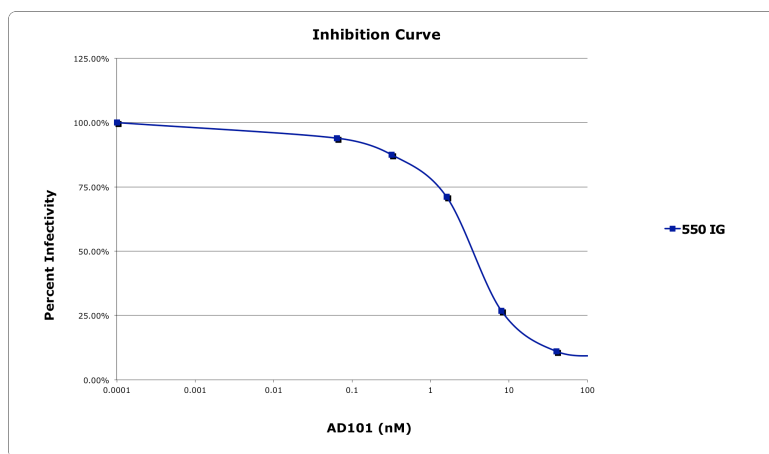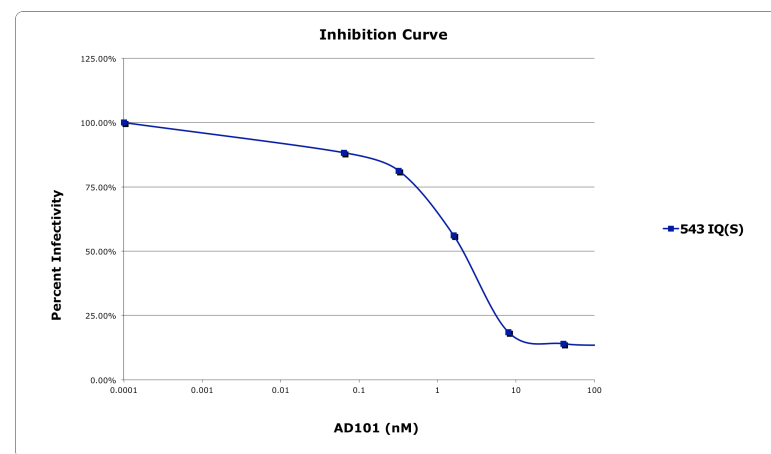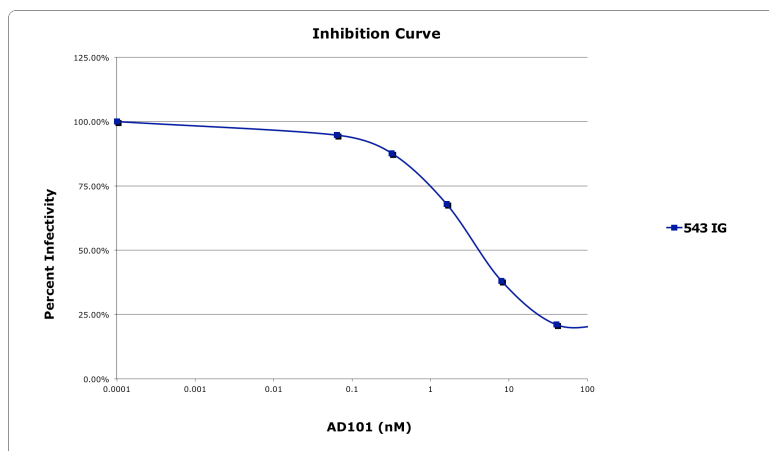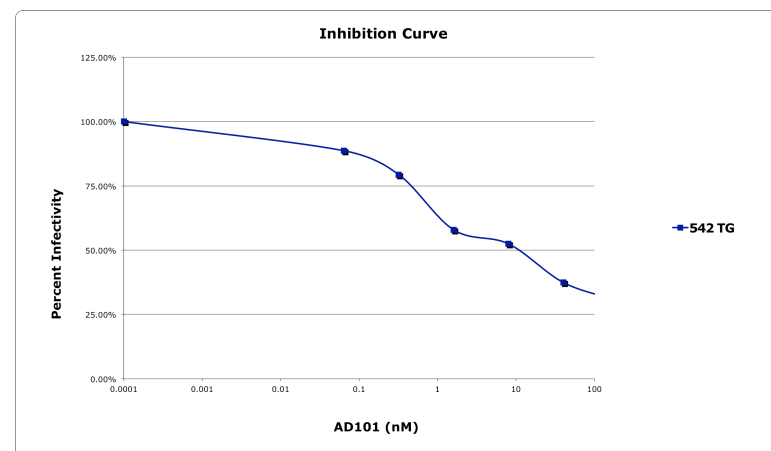

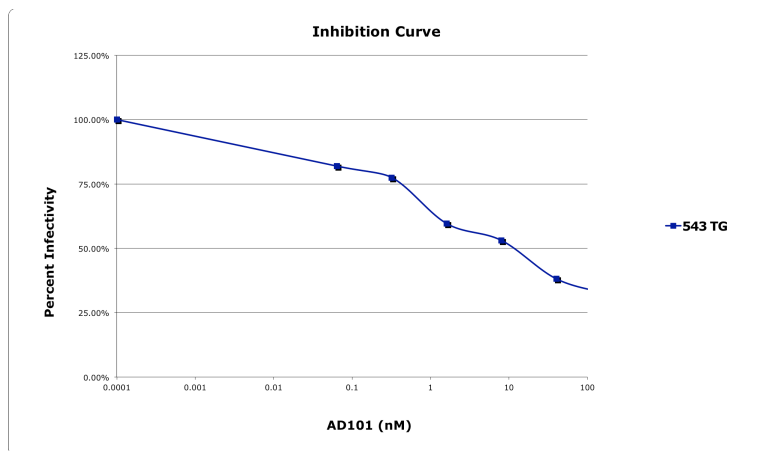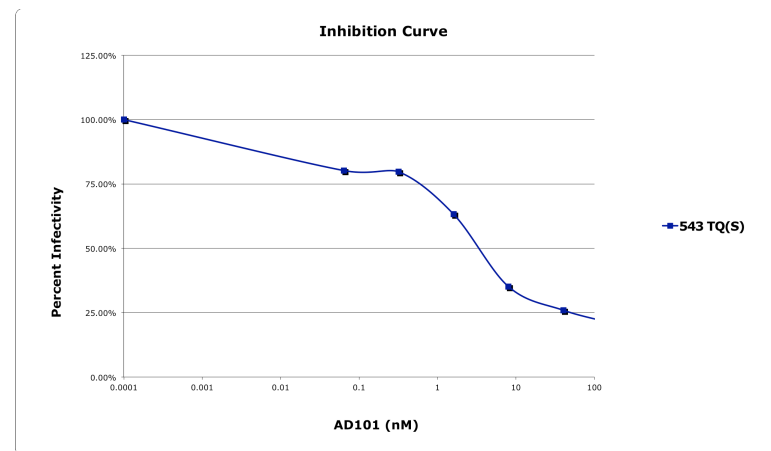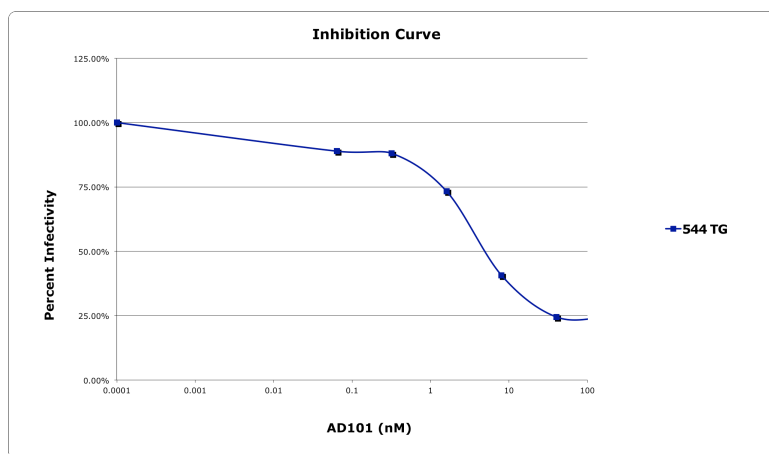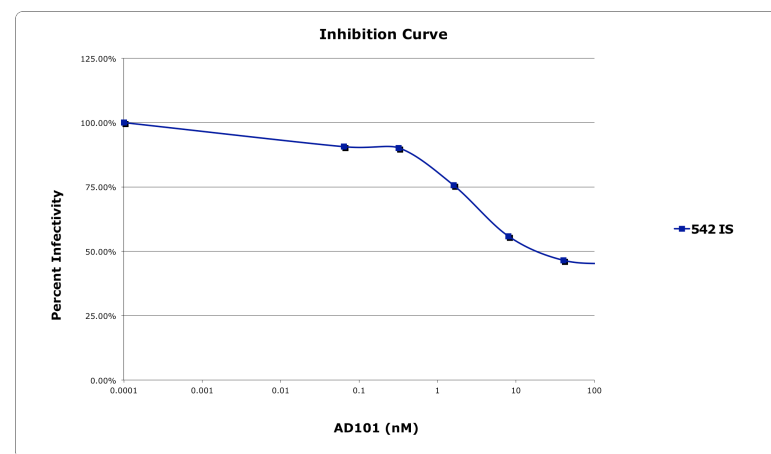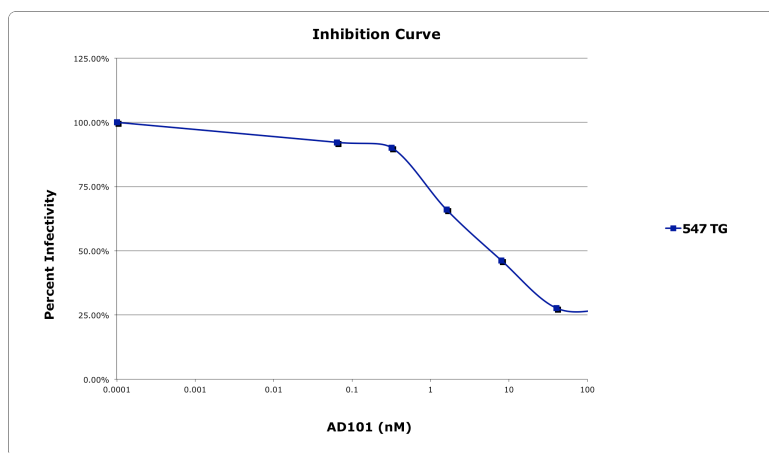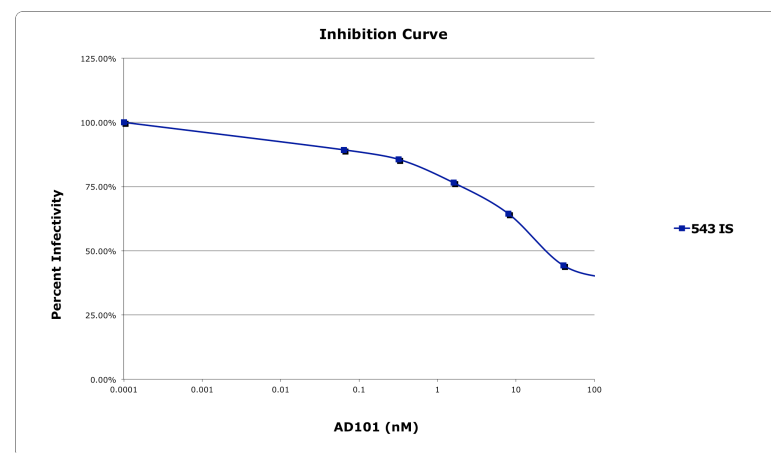

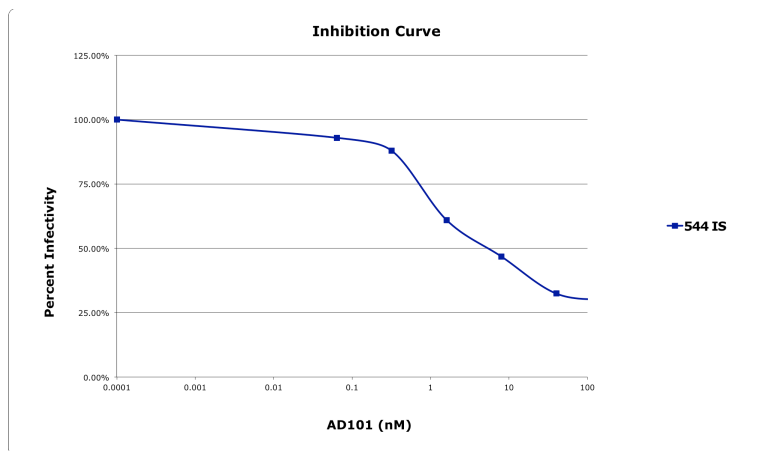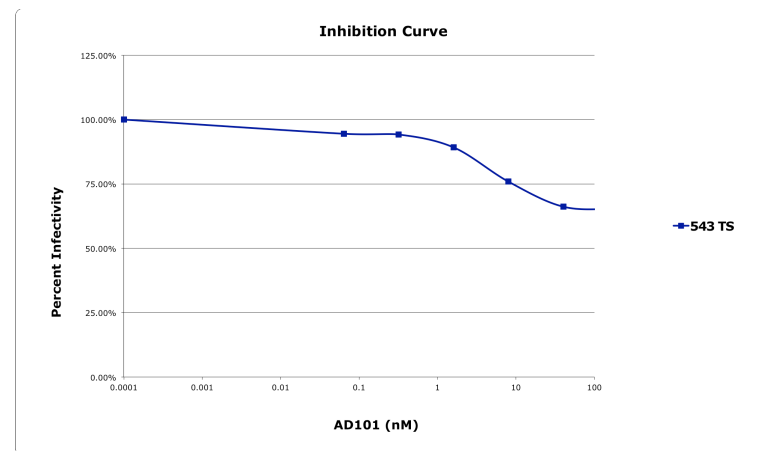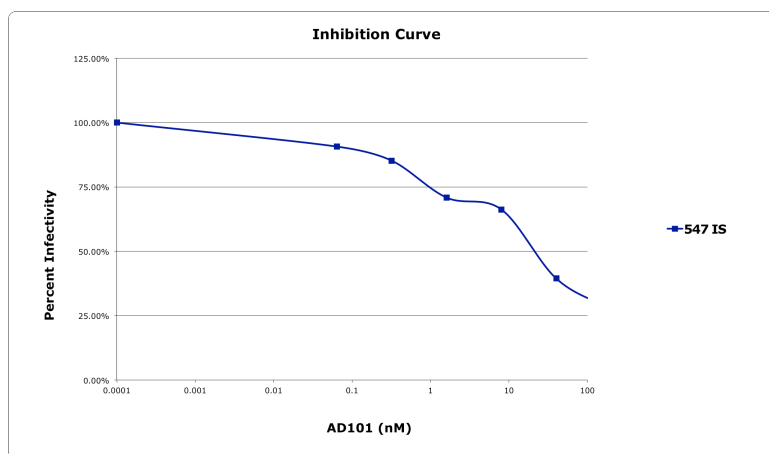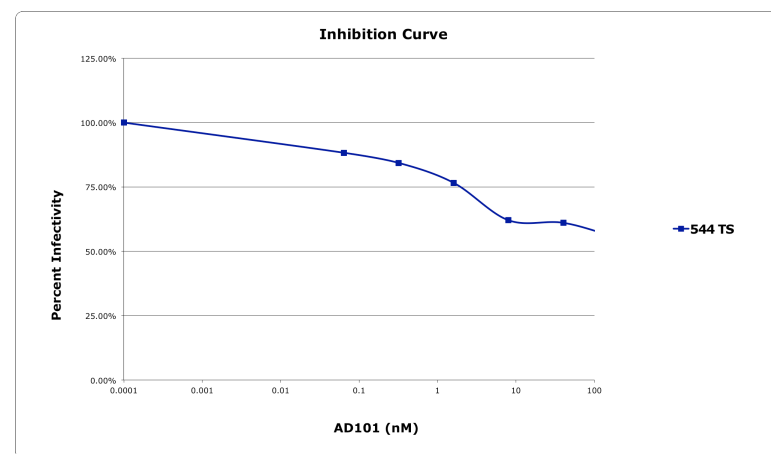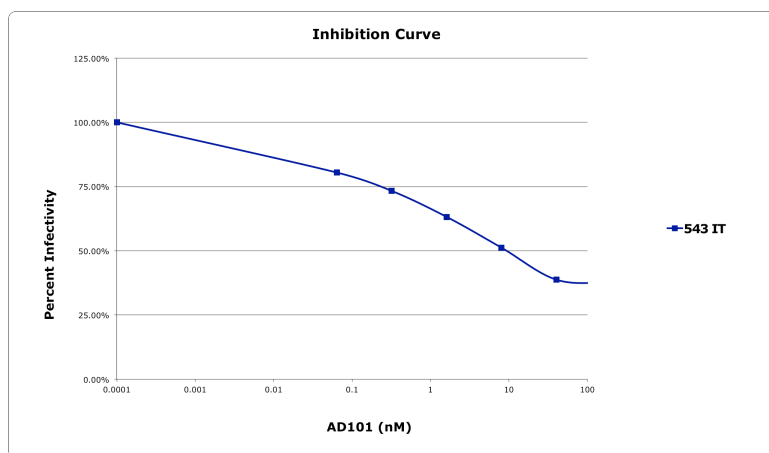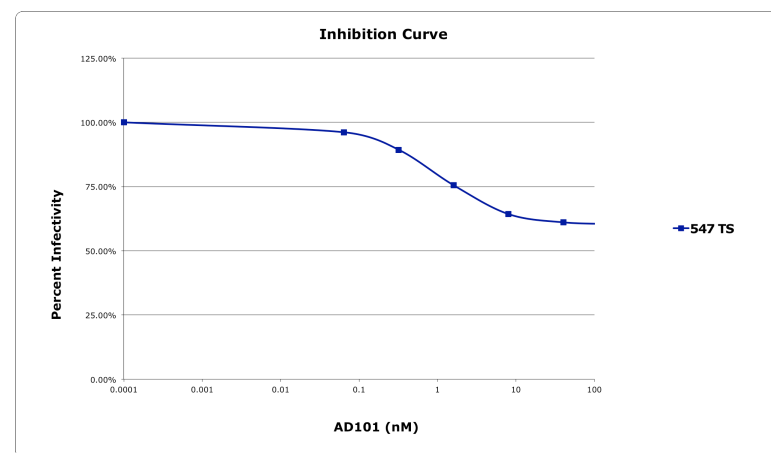

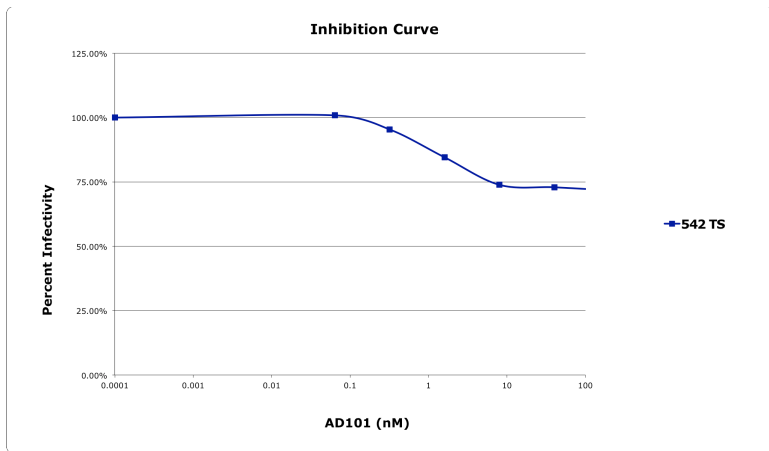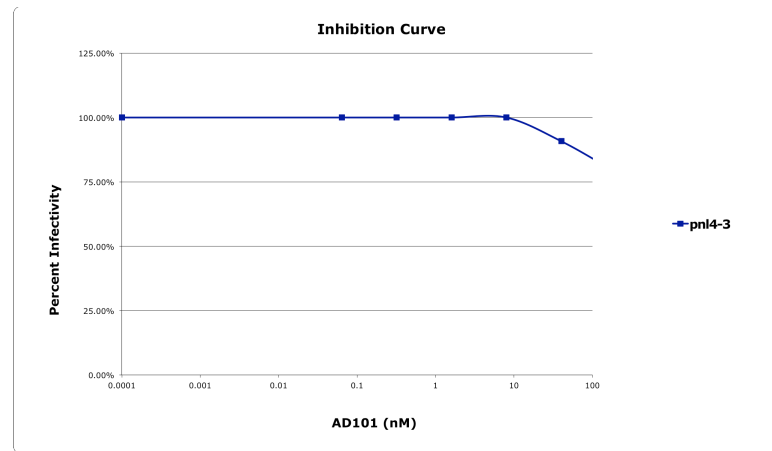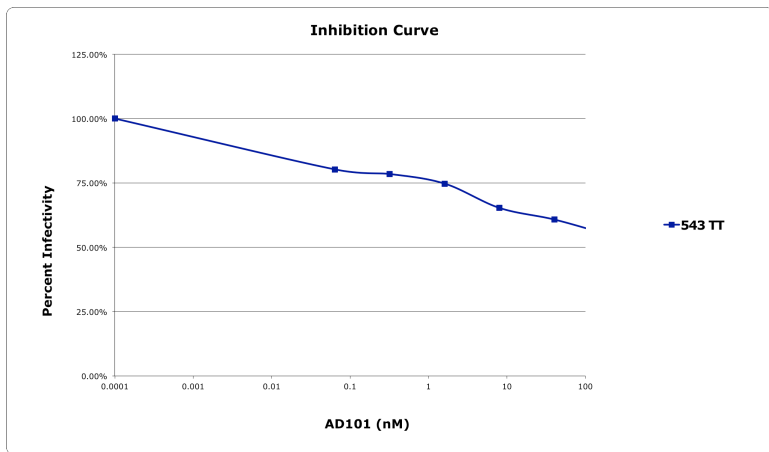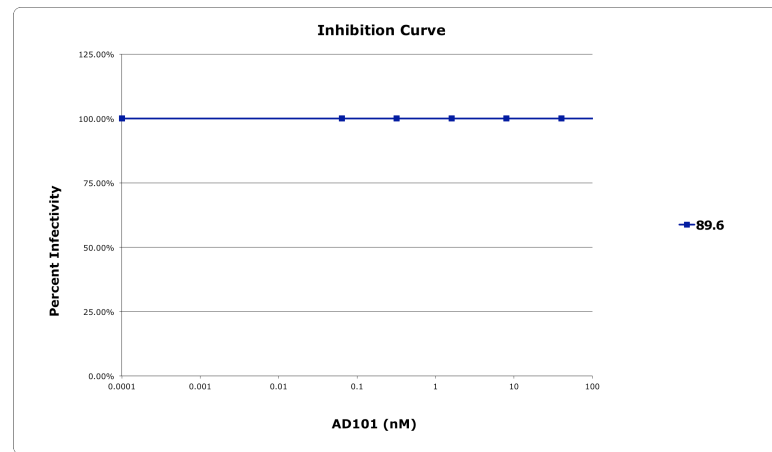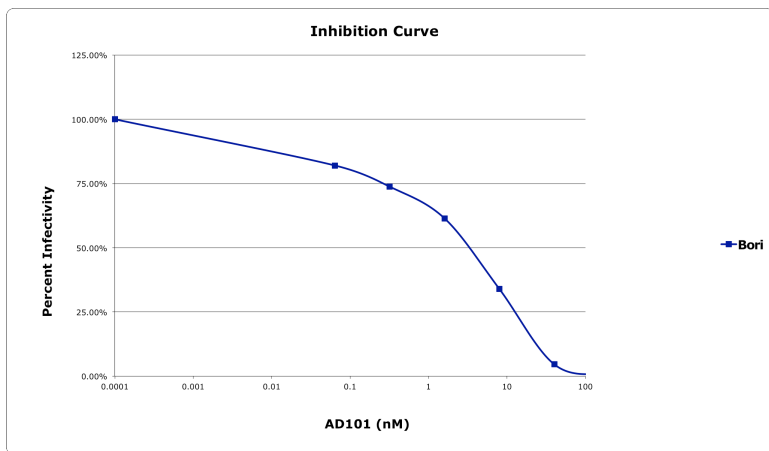

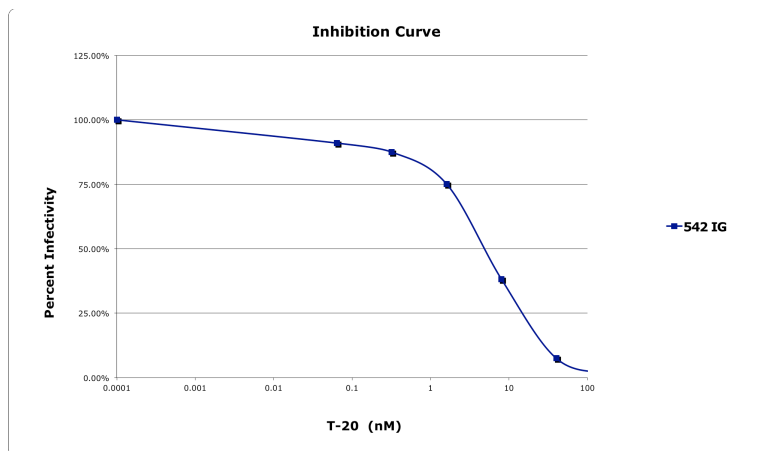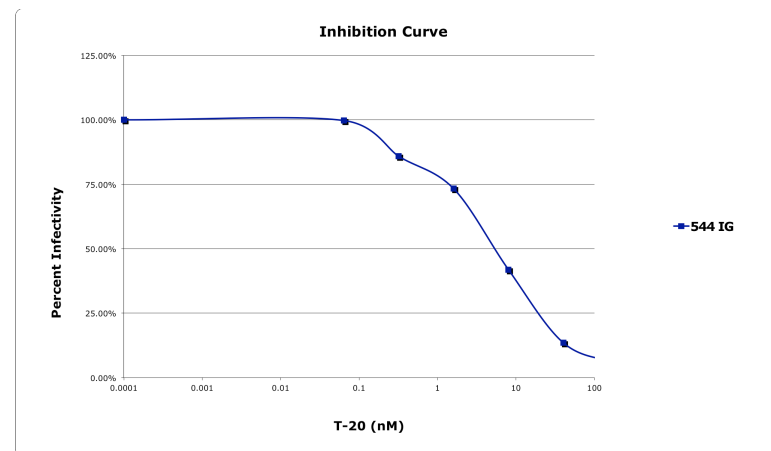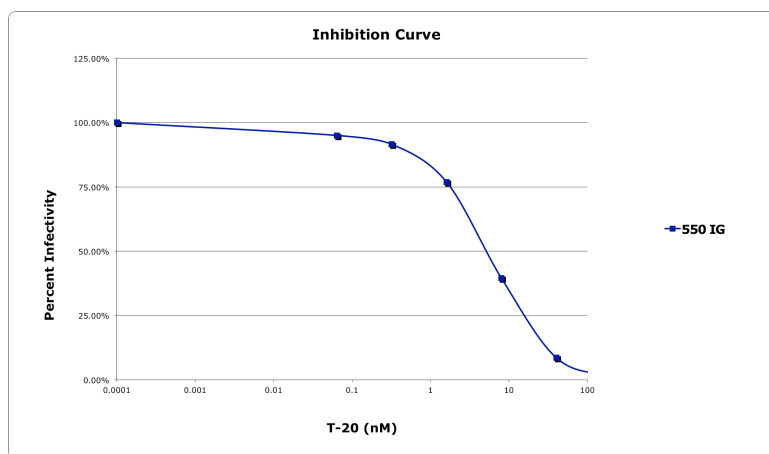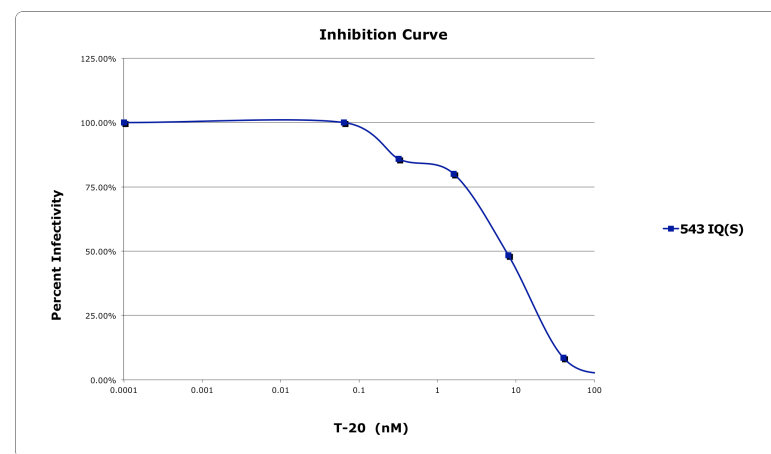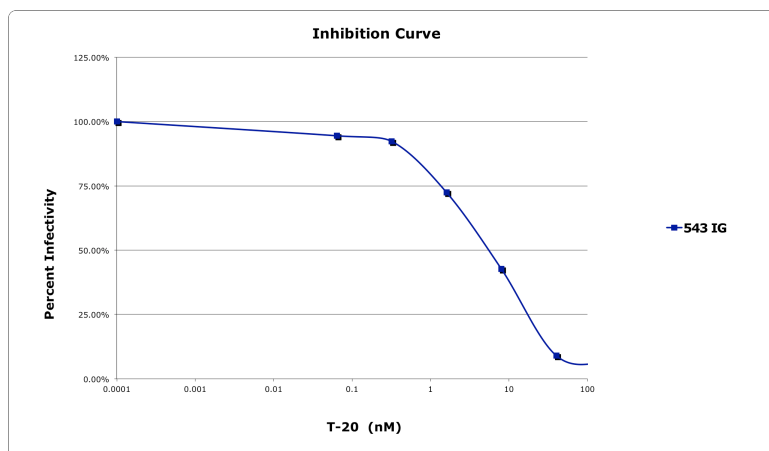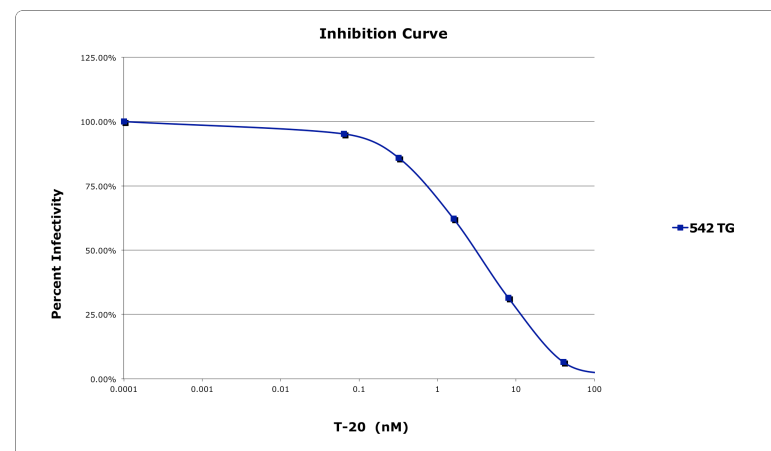

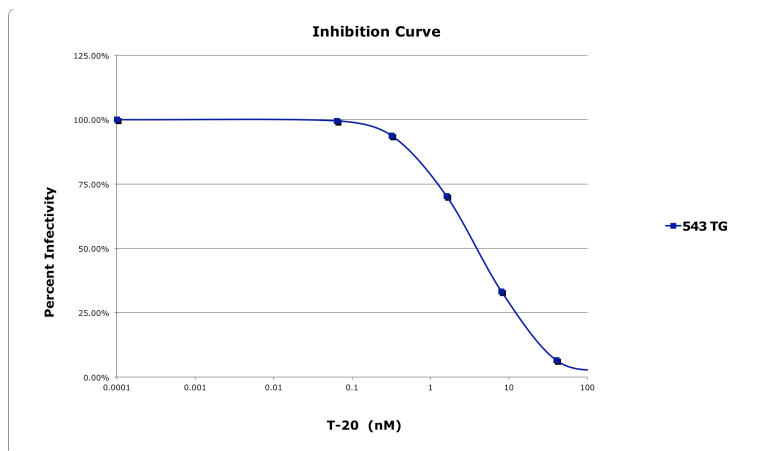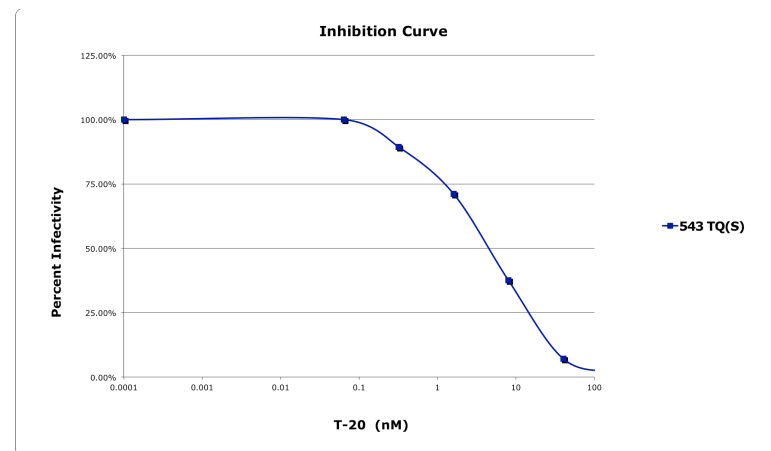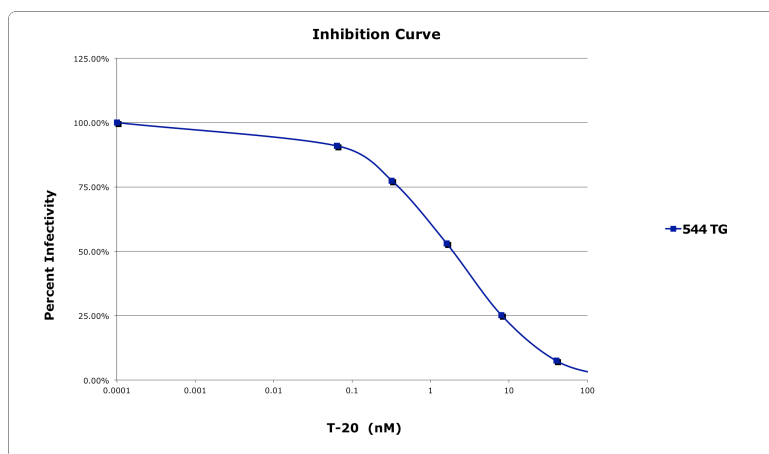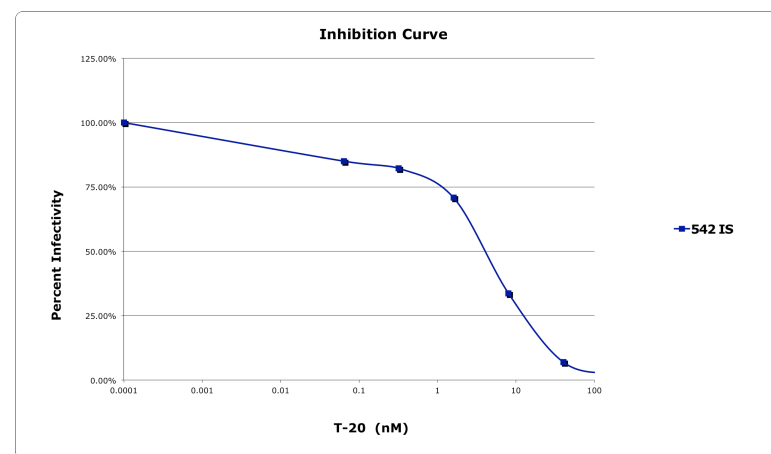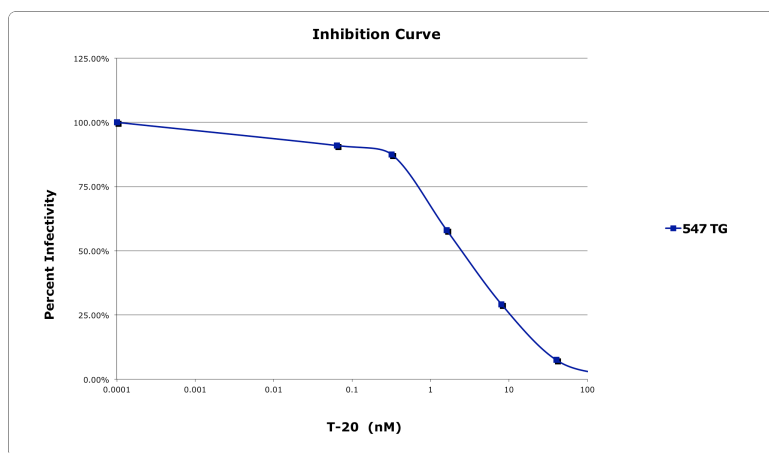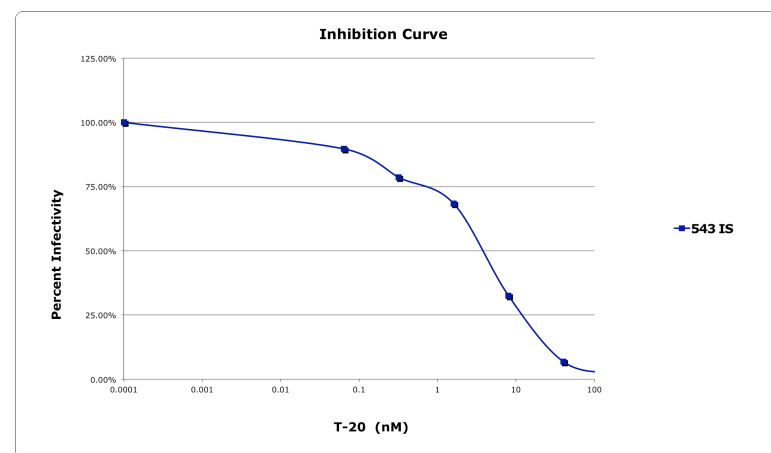

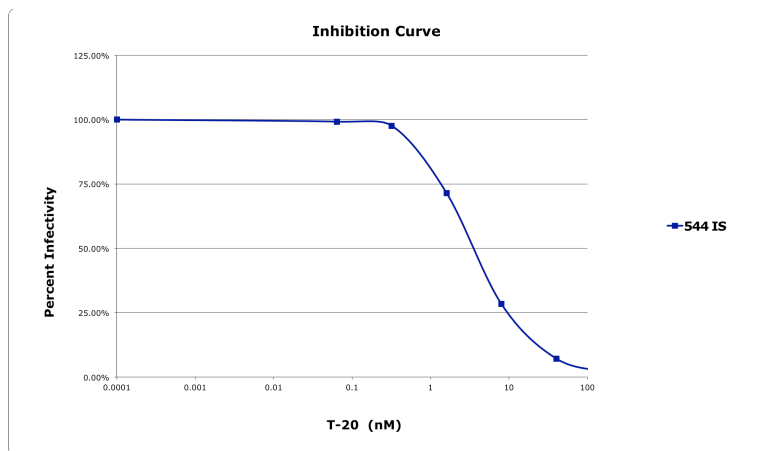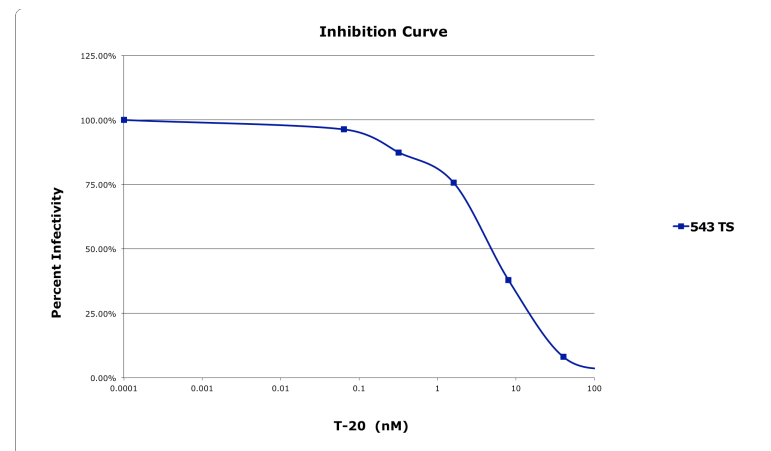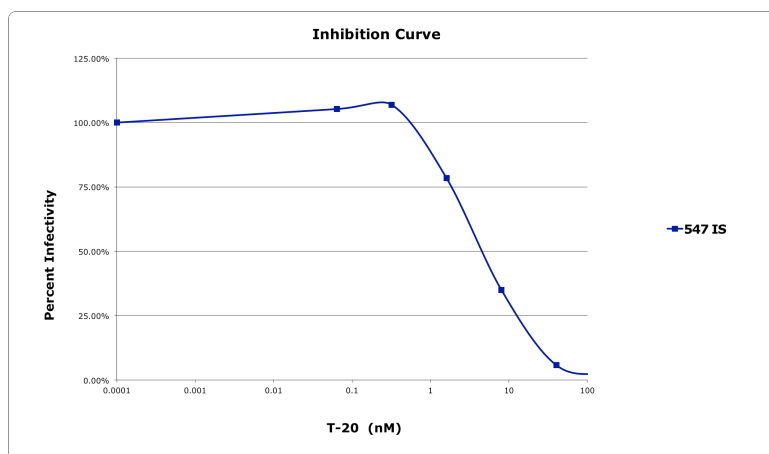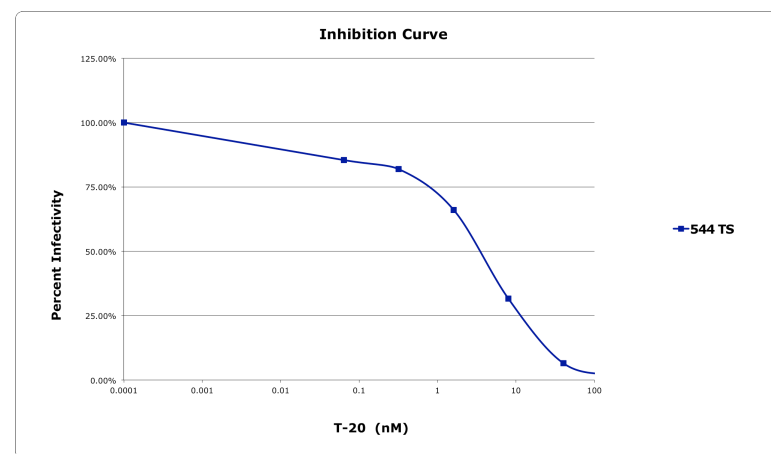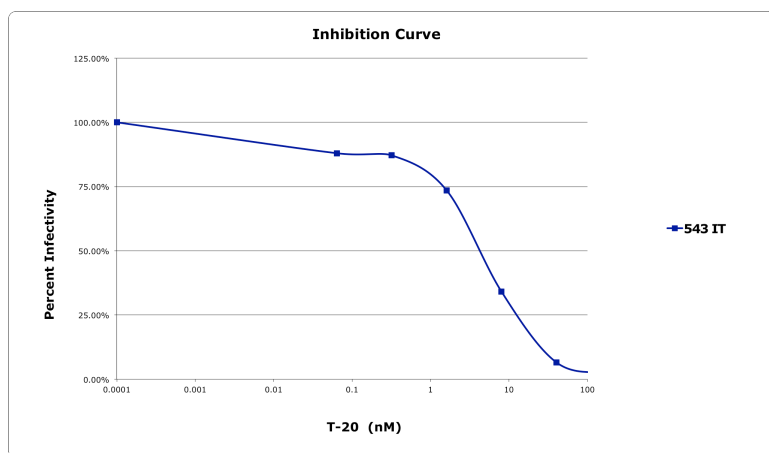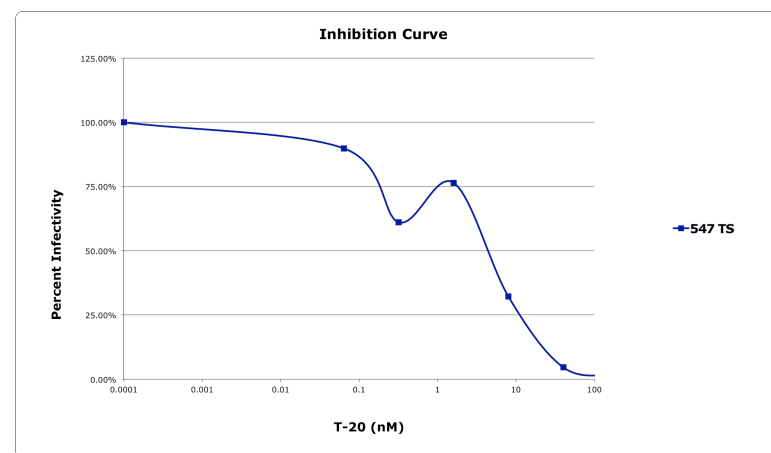

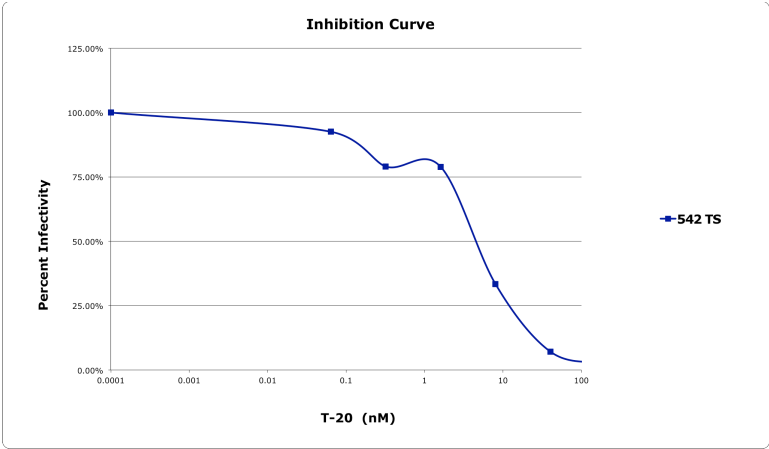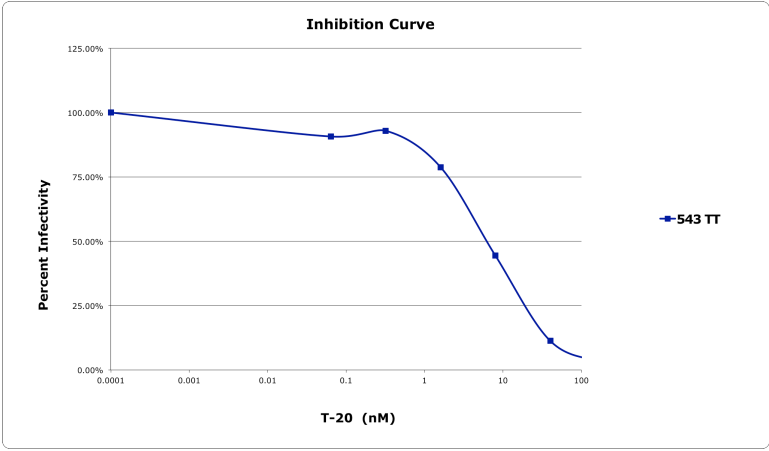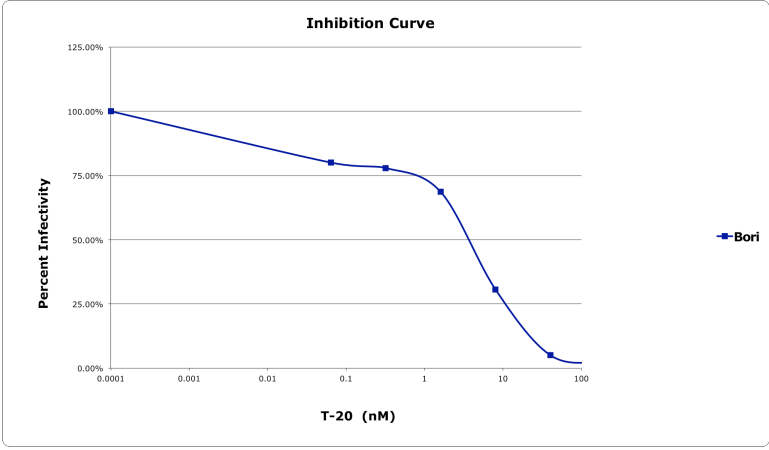

Supplement: S1 Fig — The legend lists the tested clone. Mutant sequences are identified by the parental clone (i.e. 542) and the residues present at positions 123 and 264 (i.e. 543 I+G indicates a mutant derived from parental clone 543 with an I at position 123 and a G at position 264). MRV = maraviroc, TAK-779, AD101 = SCH-350581, T20 = Fuzeon, nM = nanomolar. (PDF) [file pone.0128116.s001.pdf]
