# Supplementary material for: A Conserved Glycan in the C2 Domain of HIV-1 Envelope Acts as a Molecular Switch to Control X4 Utilization by Clonal Variants with Identical V3 Loops
Source: PLoS One. 2015 Jun 17;10(6):e0128116. doi: 10.1371/journal.pone.0128116 (PMC4471078; doi:10.1371/journal.pone.0128116)
Supplement: S2 Fig — The legend lists the tested clone. Mutant sequences are identified by the parental clone (i.e. 542) and the residues present at positions 123 and 264 (i.e. 543 I+G indicates a mutant derived from parental clone 543 with an I at position 123 and a G at position 264). GNA = Galanthus nivalis agglutinin, HHA = Hippeastrum hybrid (Amaryllis) agglutinin, UDA = Urtica dioica agglutinin, ug/ml = micrograms per milliliter. (PDF) [file pone.0128116.s002.pdf]

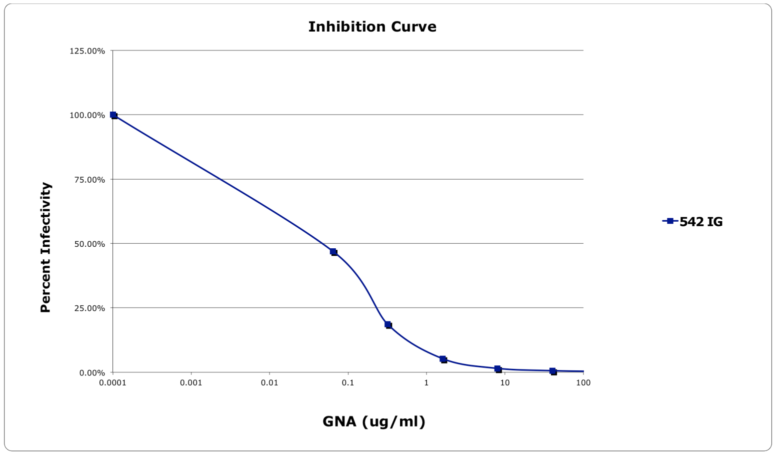
Inhibition Curve
125.00%
100.00%
75.00%
50.00%
25.00%
0.00%
Percent Infectivity
0.0001
0.001
0.01
0.1
1
10
100
GNA (ug/ml)
542 IG

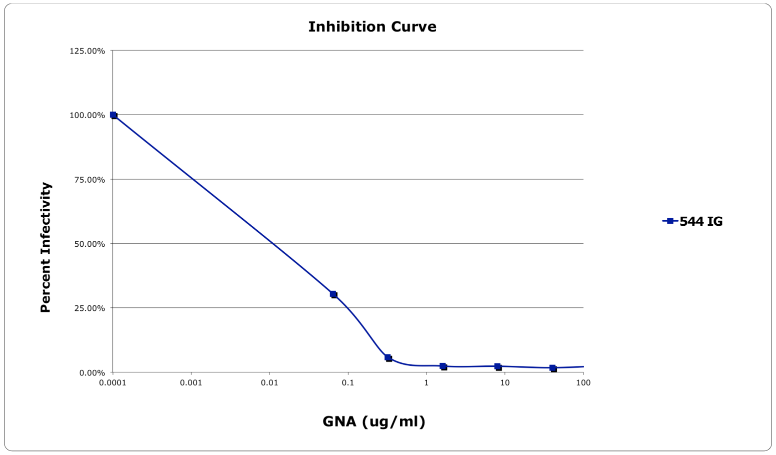
Inhibition Curve
125.00%
100.00%
75.00%
50.00%
25.00%
0.00%
Percent Infectivity
0.0001
0.001
0.01
0.1
1
10
100
GNA (ug/ml)
544 IG

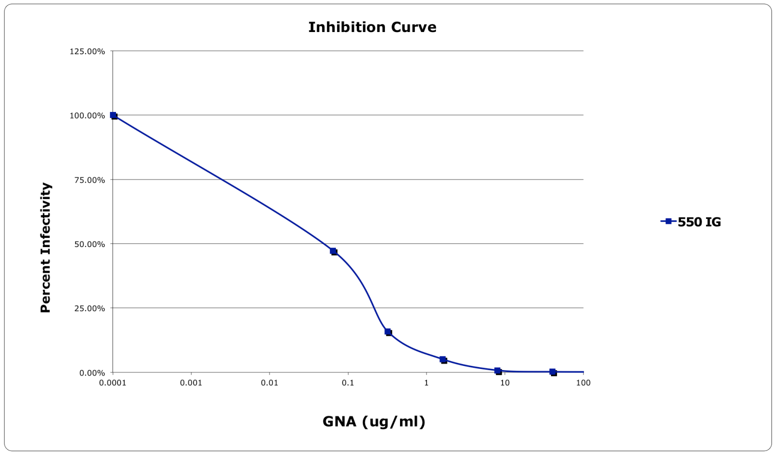
Inhibition Curve
125.00%
100.00%
75.00%
50.00%
25.00%
0.00%
Percent Infectivity
0.0001
0.001
0.01
0.1
1
10
100
GNA (ug/ml)
550 IG

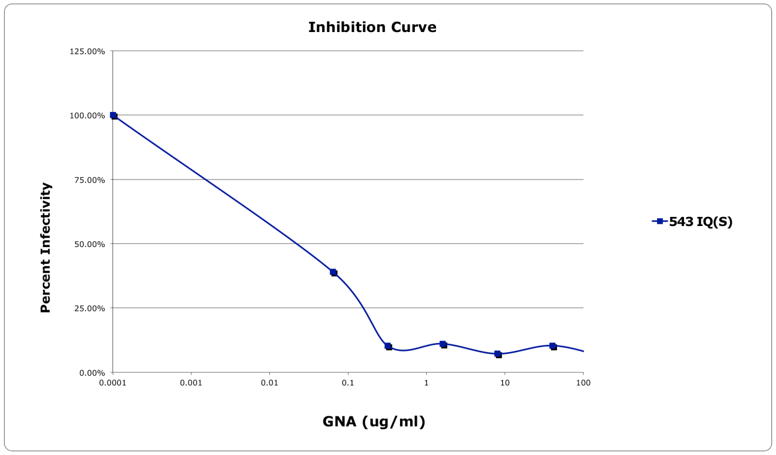
Inhibition Curve
125.00%
100.00%
75.00%
50.00%
25.00%
0.00%
Percent Infectivity
0.0001
0.001
0.01
0.1
1
10
100
GNA (ug/ml)
543 IQ(S)

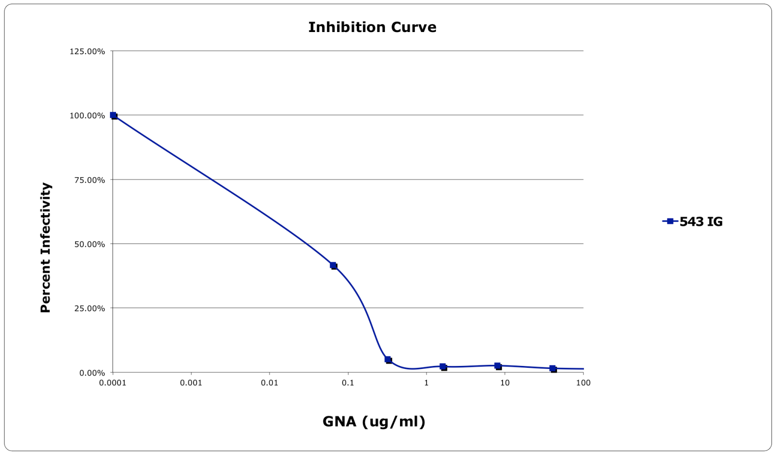
Inhibition Curve
125.00%
100.00%
75.00%
50.00%
25.00%
0.00%
Percent Infectivity
0.0001
0.001
0.01
0.1
1
10
100
GNA (ug/ml)
543 IG

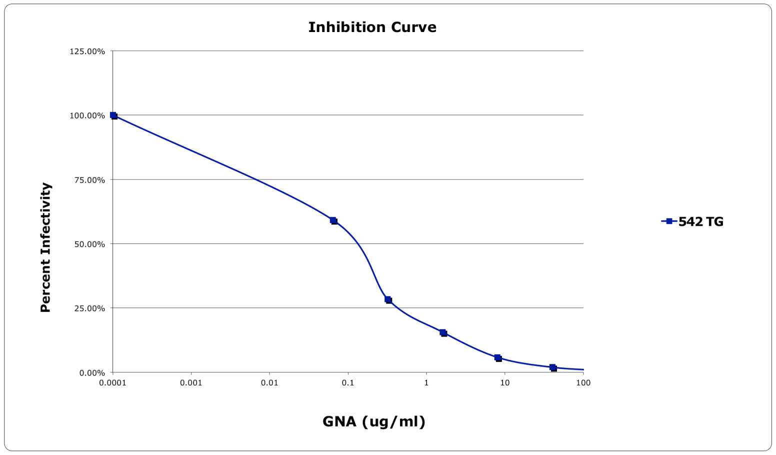
Inhibition Curve
125.00%
100.00%
75.00%
50.00%
25.00%
0.00%
Percent Infectivity
0.0001
0.001
0.01
0.1
1
10
100
GNA (ug/ml)
542 TG

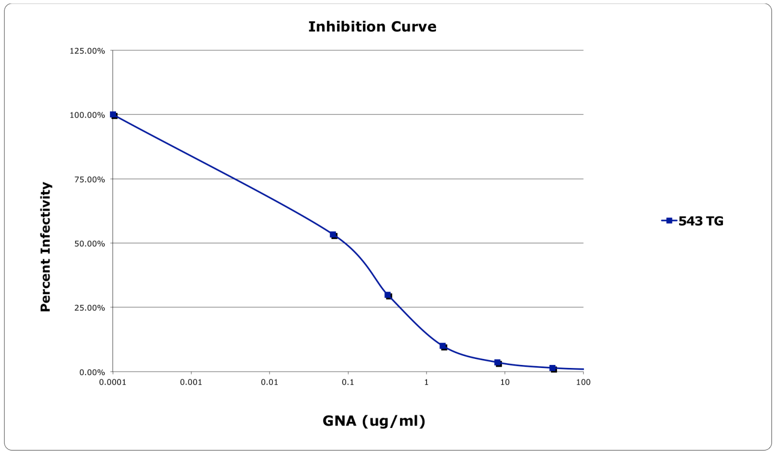
Inhibition Curve
125.00%
100.00%
75.00%
50.00%
25.00%
0.00%
Percent Infectivity
0.0001
0.001
0.01
0.1
1
10
100
GNA (ug/ml)
543 TG

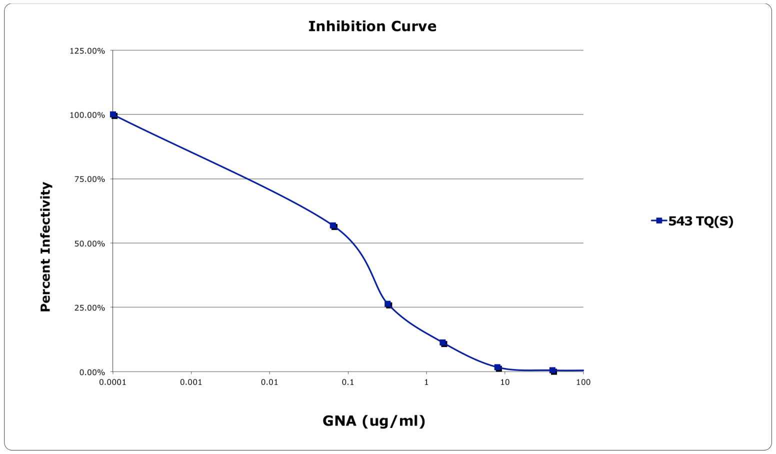
Inhibition Curve
125.00%
100.00%
75.00%
50.00%
25.00%
0.00%
Percent Infectivity
0.0001
0.001
0.01
0.1
1
10
100
GNA (ug/ml)
543 TQ(S)

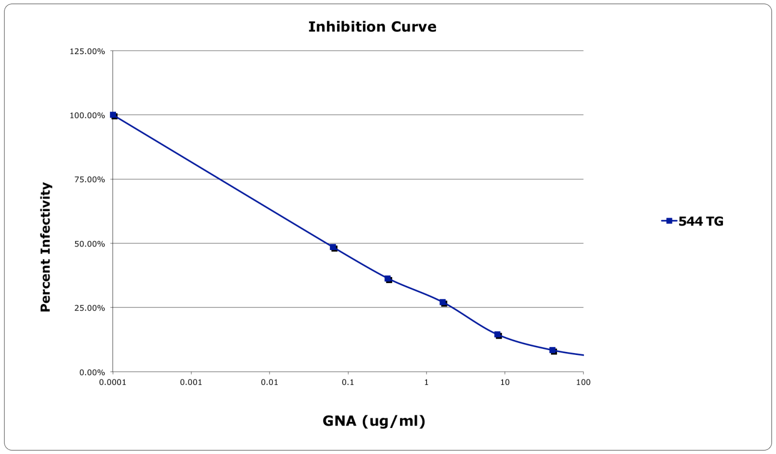
Inhibition Curve
125.00%
100.00%
75.00%
50.00%
25.00%
0.00%
Percent Infectivity
0.0001
0.001
0.01
0.1
1
10
100
GNA (ug/ml)
544 TG

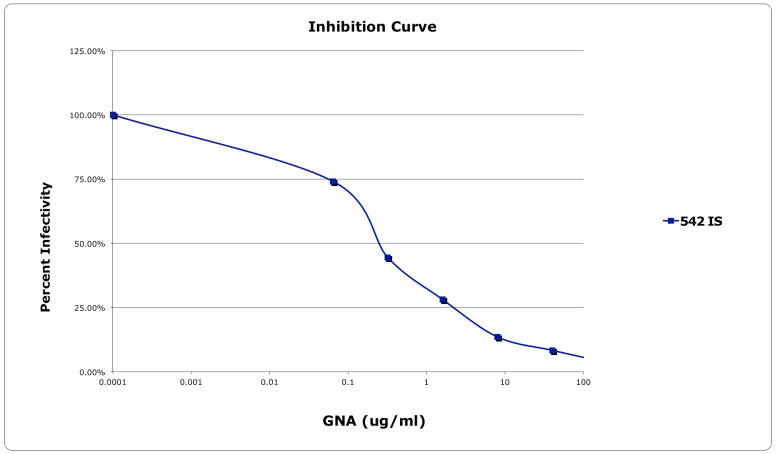
Inhibition Curve
125.00%
100.00%
75.00%
50.00%
25.00%
0.00%
Percent Infectivity
0.0001
0.001
0.01
0.1
1
10
100
GNA (ug/ml)
542 IS

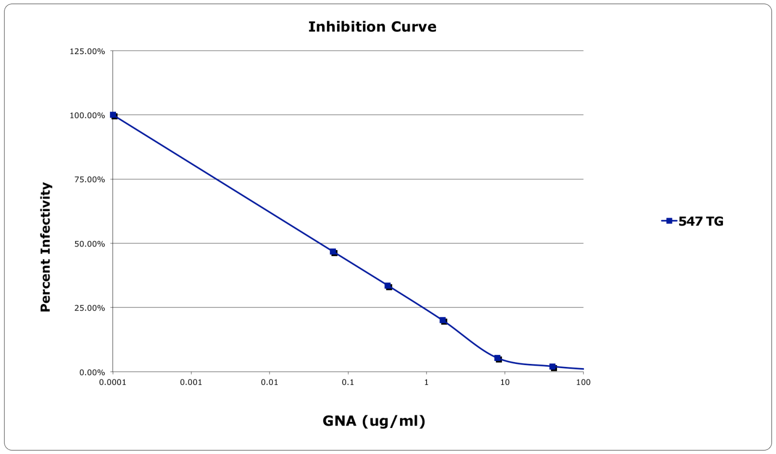
Inhibition Curve
125.00%
100.00%
75.00%
50.00%
25.00%
0.00%
Percent Infectivity
0.0001
0.001
0.01
0.1
1
10
100
GNA (ug/ml)
547 TG

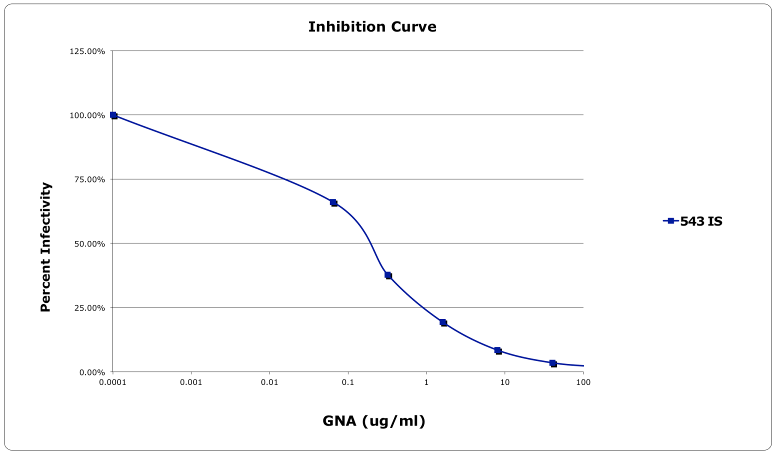
Inhibition Curve
125.00%
100.00%
75.00%
50.00%
25.00%
0.00%
Percent Infectivity
0.0001
0.001
0.01
0.1
1
10
100
GNA (ug/ml)
543 IS

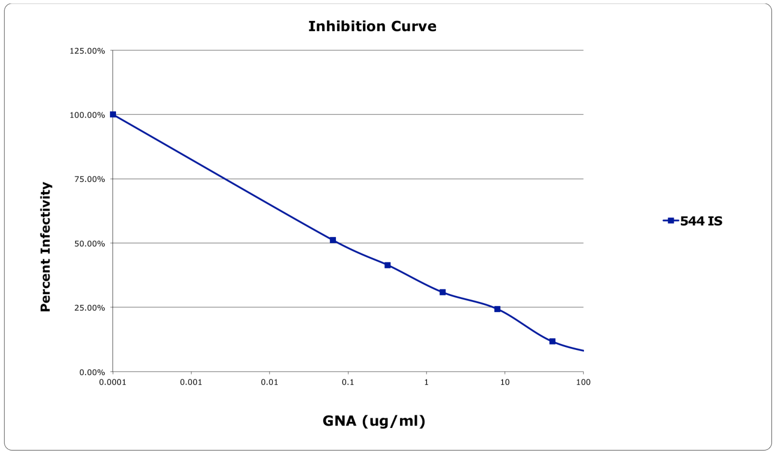
Inhibition Curve
125.00%
100.00%
75.00%
50.00%
25.00%
0.00%
Percent Infectivity
0.0001
0.001
0.01
0.1
1
10
100
GNA (ug/ml)
544 IS

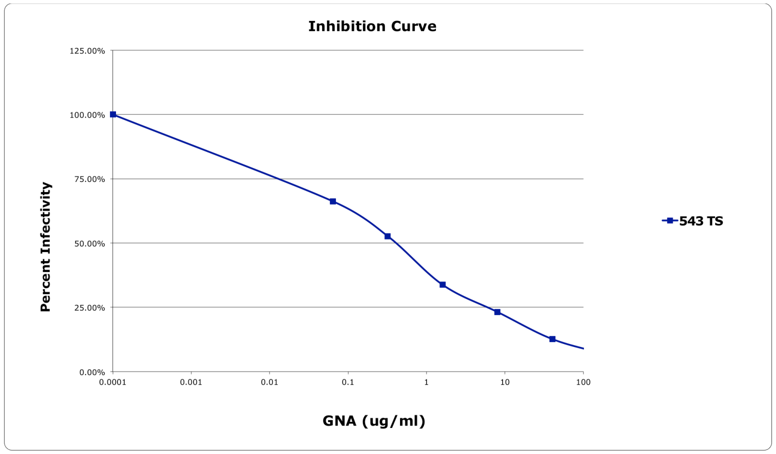
Inhibition Curve
125.00%
100.00%
75.00%
50.00%
25.00%
0.00%
Percent Infectivity
0.0001
0.001
0.01
0.1
1
10
100
GNA (ug/ml)
543 TS

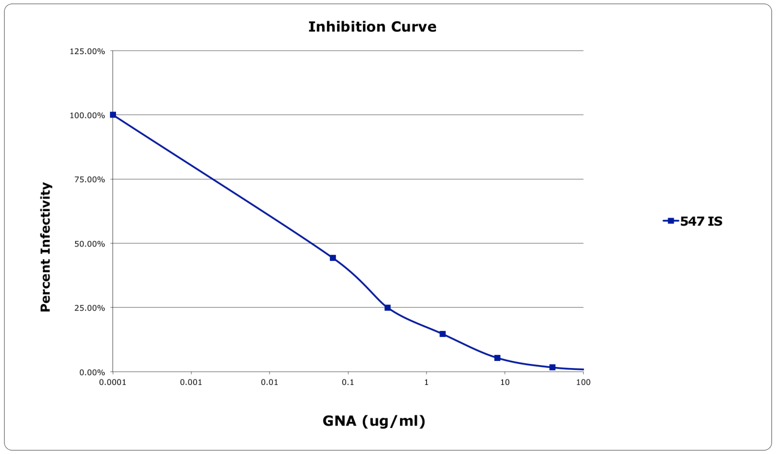
Inhibition Curve
125.00%
100.00%
75.00%
50.00%
25.00%
0.00%
Percent Infectivity
0.0001
0.001
0.01
0.1
1
10
100
GNA (ug/ml)
547 IS

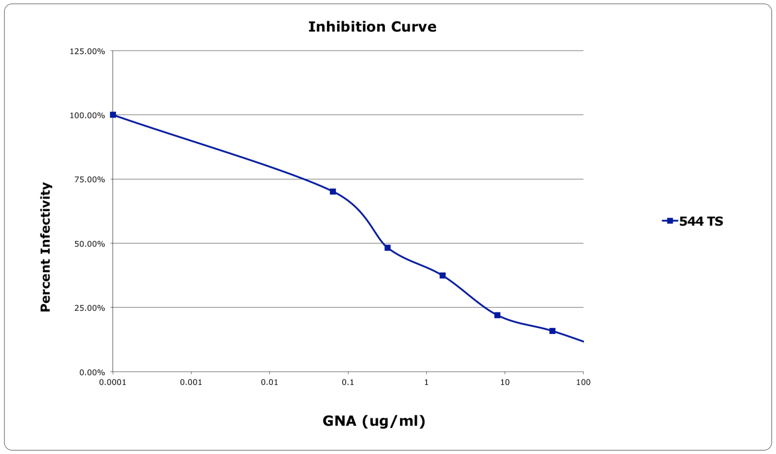
Inhibition Curve
125.00%
100.00%
75.00%
50.00%
25.00%
0.00%
Percent Infectivity
0.0001
0.001
0.01
0.1
1
10
100
GNA (ug/ml)
544 TS

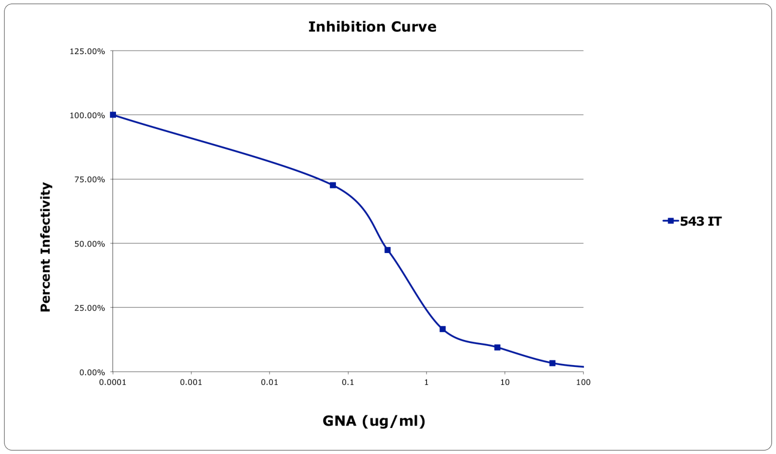
Inhibition Curve
125.00%
100.00%
75.00%
50.00%
25.00%
0.00%
Percent Infectivity
0.0001
0.001
0.01
0.1
1
10
100
GNA (ug/ml)
543 IT

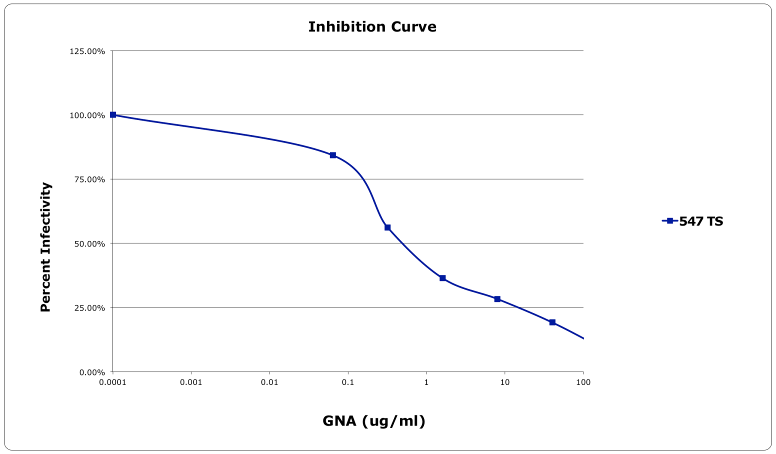
Inhibition Curve
125.00%
100.00%
75.00%
50.00%
25.00%
0.00%
Percent Infectivity
0.0001
0.001
0.01
0.1
1
10
100
GNA (ug/ml)
547 TS

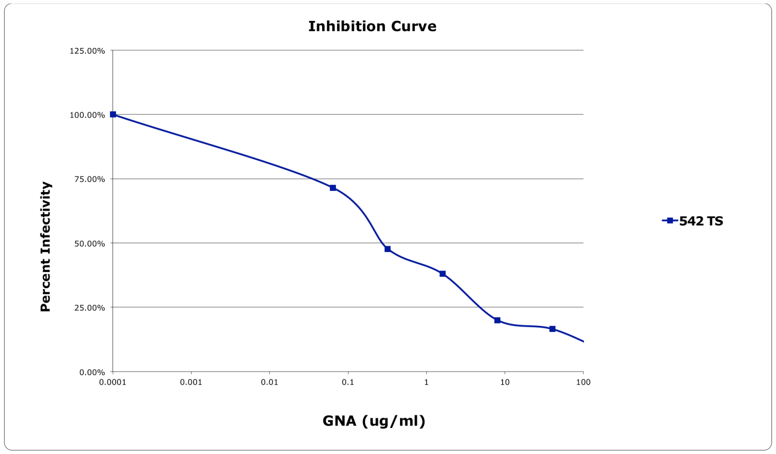
Inhibition Curve
Percent Infectivity
125.00%
100.00%
75.00%
50.00%
25.00%
0.00%
0.0001
0.001
0.01
0.1
1
10
100
GNA (ug/ml)
542 TS

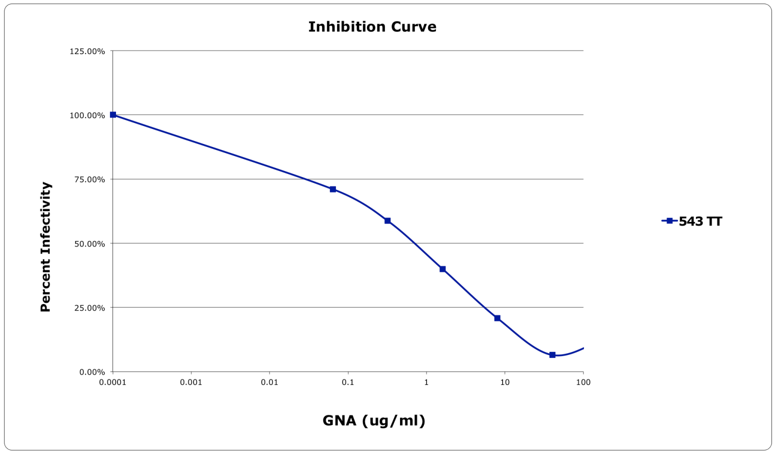
Inhibition Curve
Percent Infectivity
125.00%
100.00%
75.00%
50.00%
25.00%
0.00%
0.0001
0.001
0.01
0.1
1
10
100
GNA (ug/ml)
543 TT

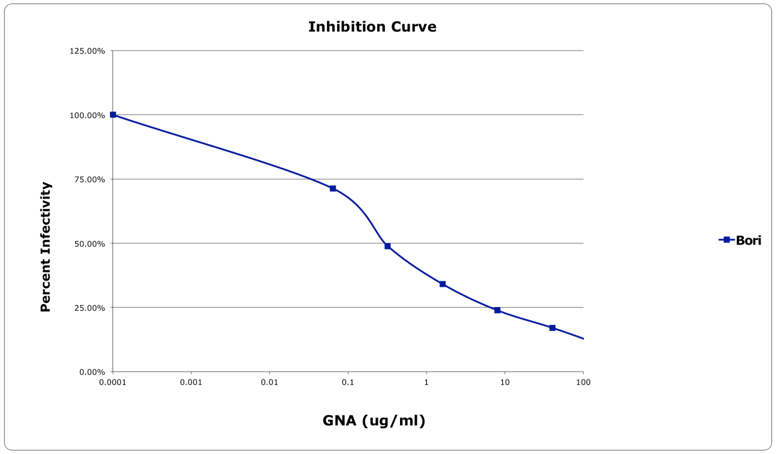
Inhibition Curve
Percent Infectivity
125.00%
100.00%
75.00%
50.00%
25.00%
0.00%
0.0001
0.001
0.01
0.1
1
10
100
GNA (ug/ml)
Bori

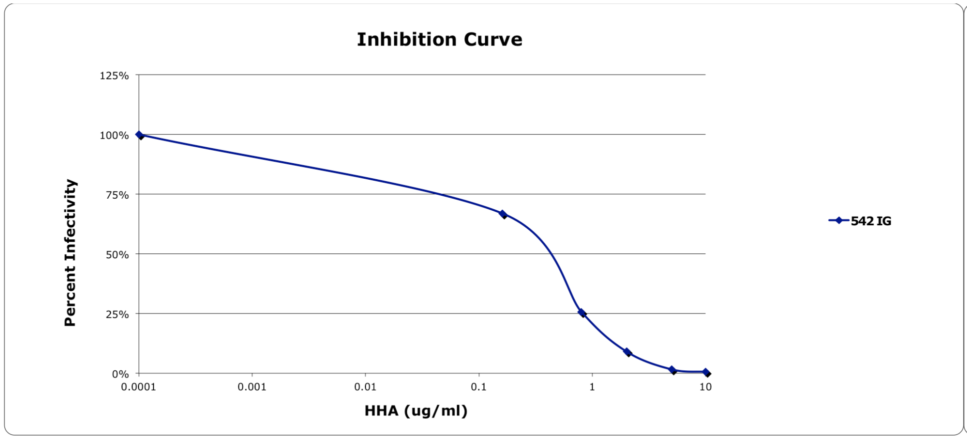
Inhibition Curve
125%
100%
75%
50%
25%
0%
Percent Infectivity
0.0001
0.001
0.01
0.1
1
10
HHA (ug/ml)
542 IG

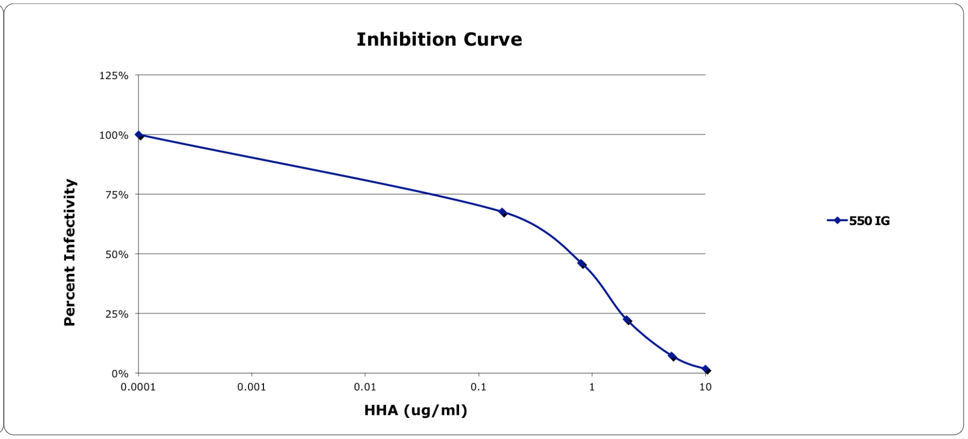
Inhibition Curve
125%
100%
75%
50%
25%
0%
Percent Infectivity
0.0001
0.001
0.01
0.1
1
10
HHA (ug/ml)
550 IG

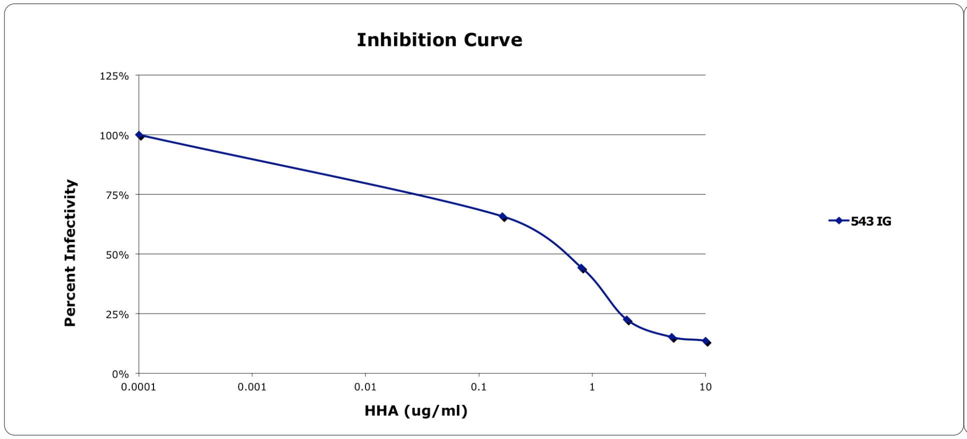
Inhibition Curve
125%
100%
75%
50%
25%
0%
Percent Infectivity
0.0001
0.001
0.01
0.1
1
10
HHA (ug/ml)
543 IG

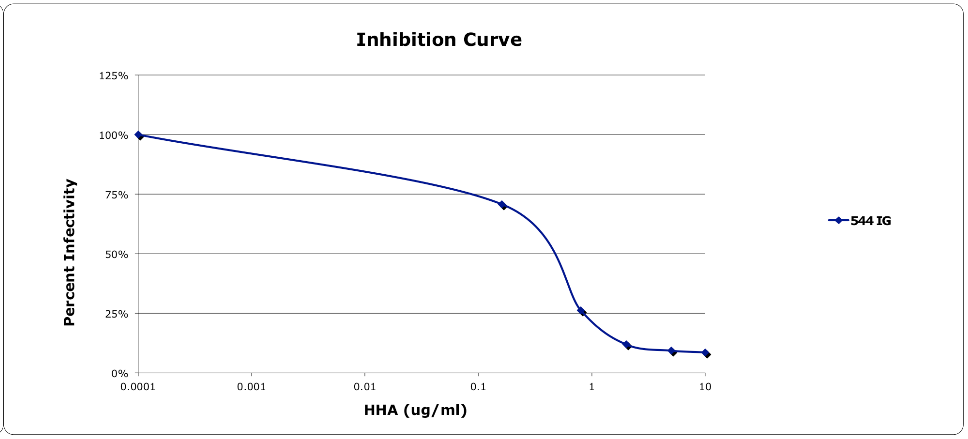
Inhibition Curve
125%
100%
75%
50%
25%
0%
Percent Infectivity
0.0001
0.001
0.01
0.1
1
10
HHA (ug/ml)
544 IG

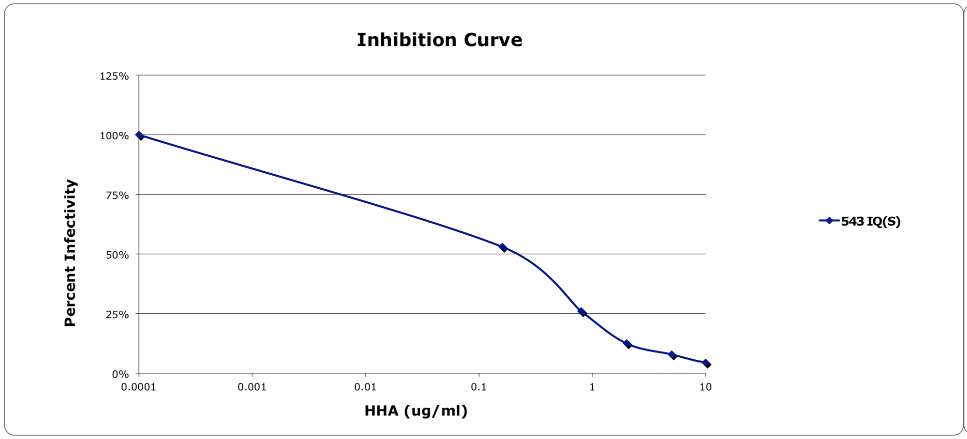
Inhibition Curve
125%
100%
75%
50%
25%
0%
Percent Infectivity
0.0001
0.001
0.01
0.1
1
10
HHA (ug/ml)
543 IQ(S)

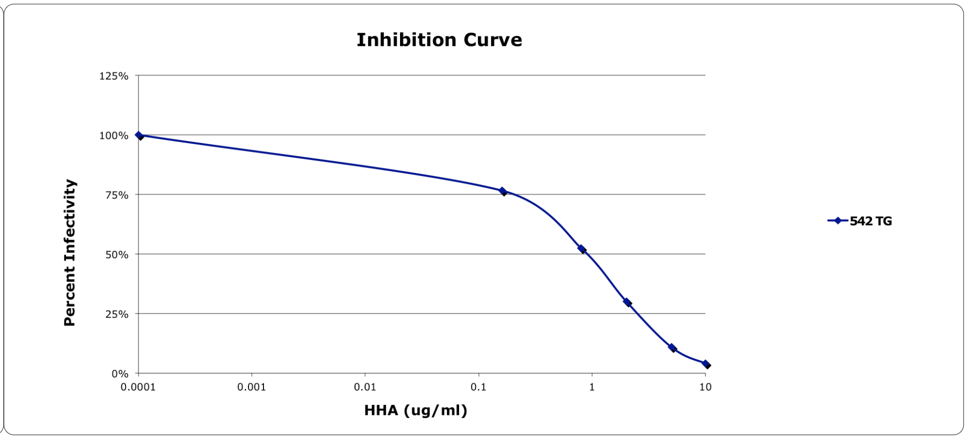
Inhibition Curve
125%
100%
75%
50%
25%
0%
Percent Infectivity
0.0001
0.001
0.01
0.1
1
10
HHA (ug/ml)
542 TG

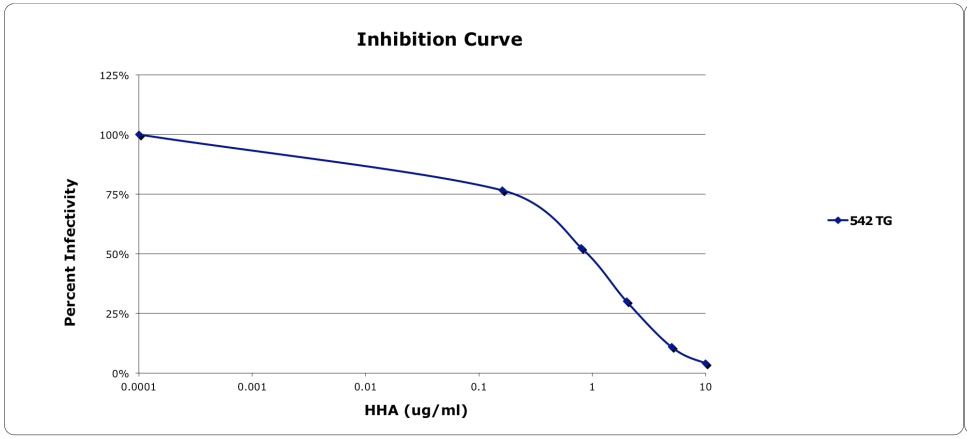
Inhibition Curve
Percent Infectivity
125%
100%
75%
50%
25%
0%
0.0001
0.001
0.01
0.1
1
10
HHA (ug/ml)
542 TG

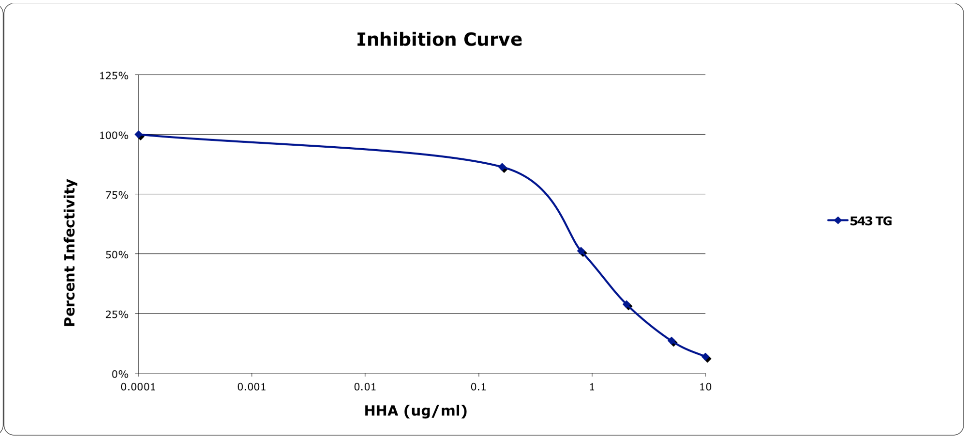
Inhibition Curve
Percent Infectivity
125%
100%
75%
50%
25%
0%
0.0001
0.001
0.01
0.1
1
10
HHA (ug/ml)
543 TG

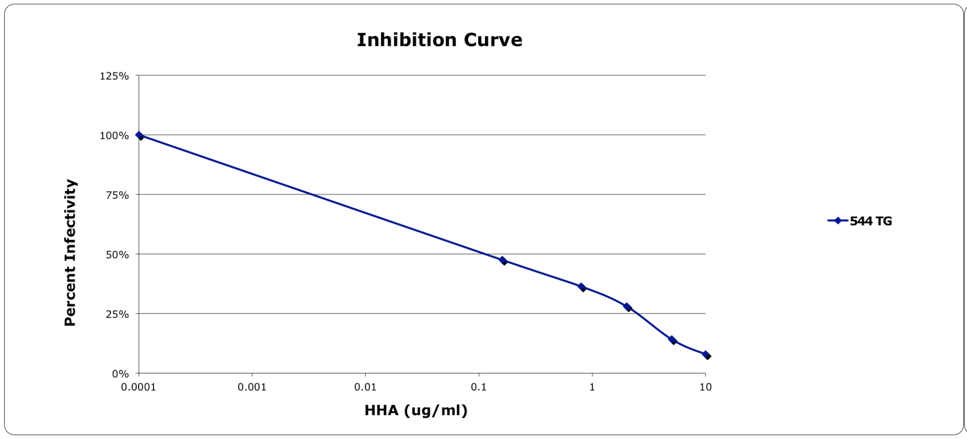
Inhibition Curve
Percent Infectivity
125%
100%
75%
50%
25%
0%
0.0001
0.001
0.01
0.1
1
10
HHA (ug/ml)
544 TG

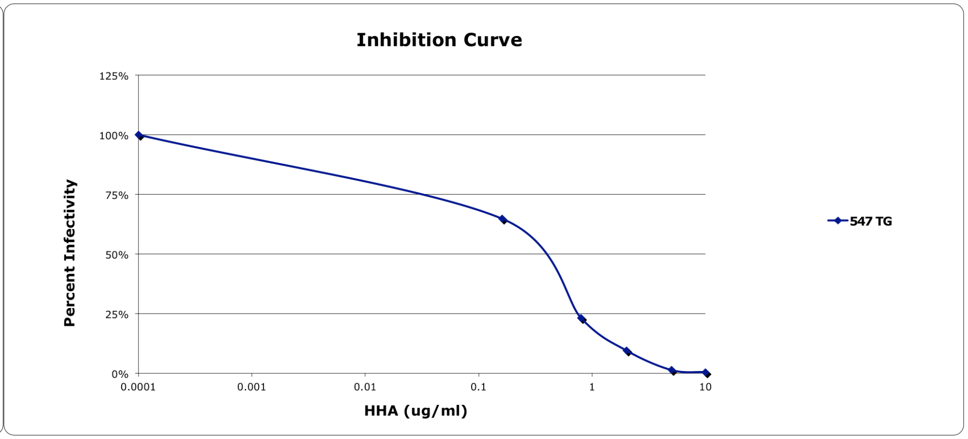
Inhibition Curve
Percent Infectivity
125%
100%
75%
50%
25%
0%
0.0001
0.001
0.01
0.1
1
10
HHA (ug/ml)
547 TG

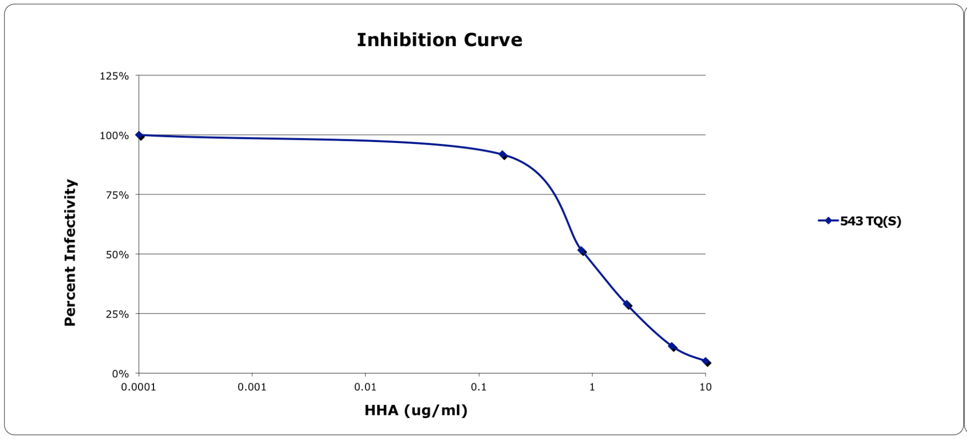
Inhibition Curve
Percent Infectivity
125%
100%
75%
50%
25%
0%
0.0001
0.001
0.01
0.1
1
10
HHA (ug/ml)
543 TQ(S)

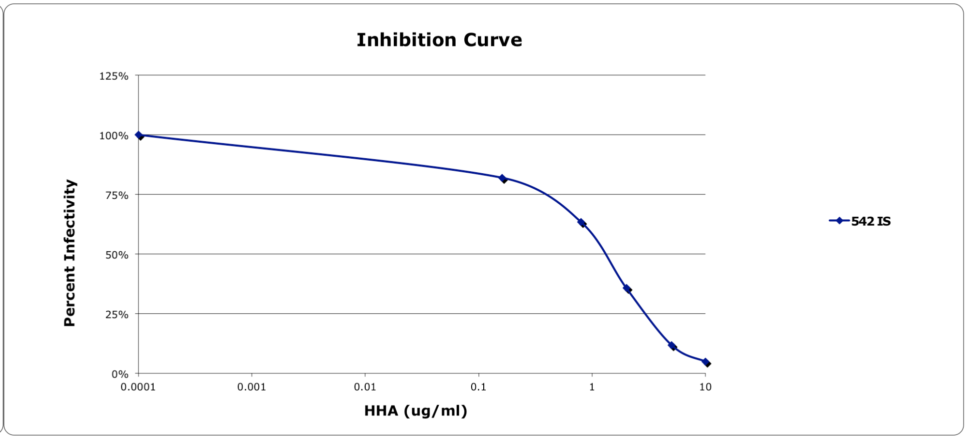
Inhibition Curve
Percent Infectivity
125%
100%
75%
50%
25%
0%
0.0001
0.001
0.01
0.1
1
10
HHA (ug/ml)
542 IS

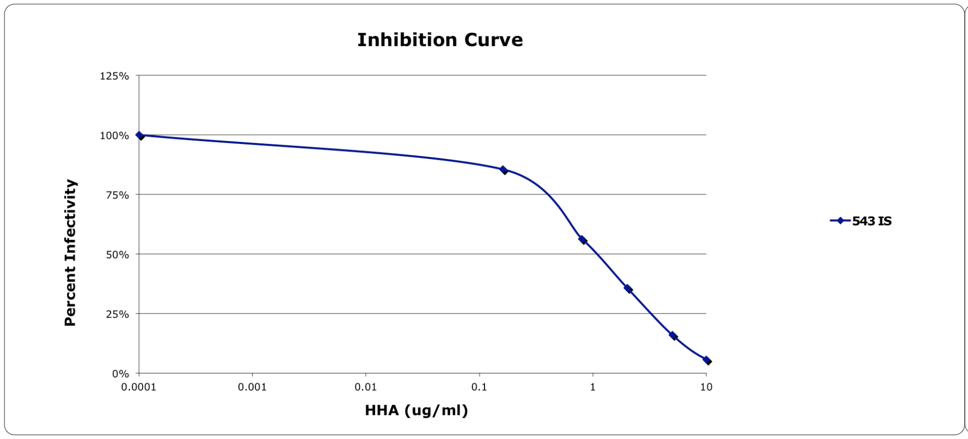
Inhibition Curve
Percent Infectivity
125%
100%
75%
50%
25%
0%
0.0001
0.001
0.01
0.1
1
10
HHA (ug/ml)
543 IS

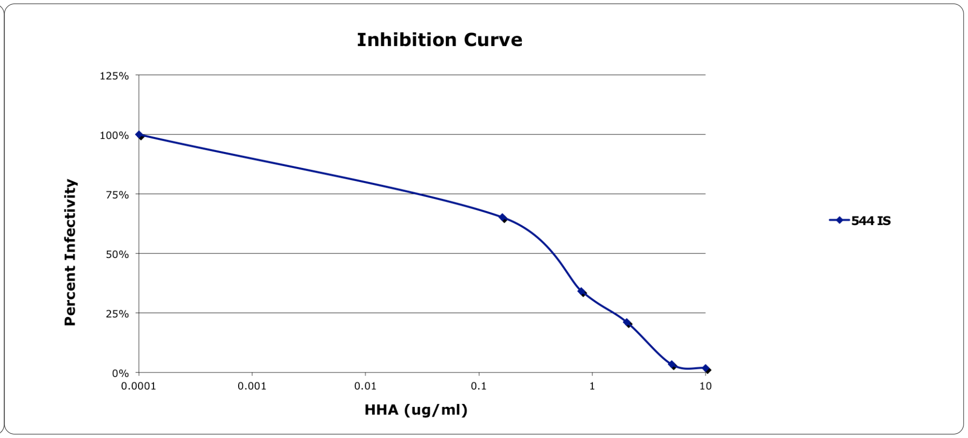
Inhibition Curve
Percent Infectivity
125%
100%
75%
50%
25%
0%
0.0001
0.001
0.01
0.1
1
10
HHA (ug/ml)
544 IS

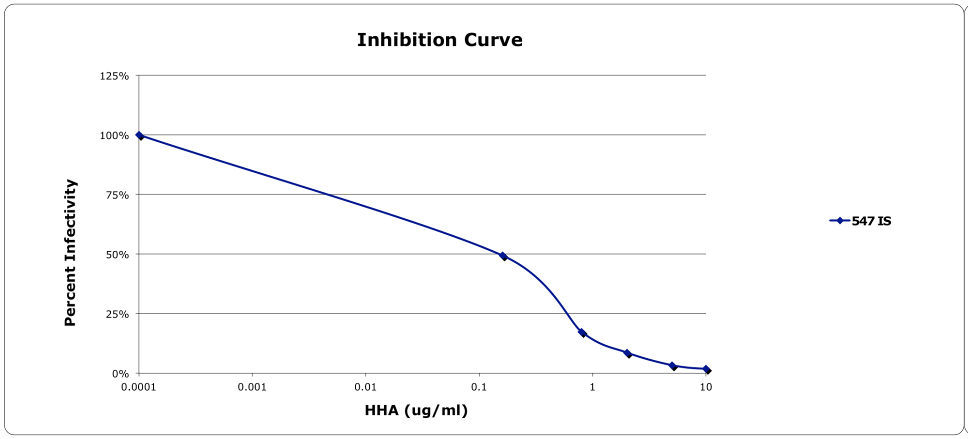
Inhibition Curve
Percent Infectivity
125%
100%
75%
50%
25%
0%
0.0001
0.001
0.01
0.1
1
10
HHA (ug/ml)
547 IS

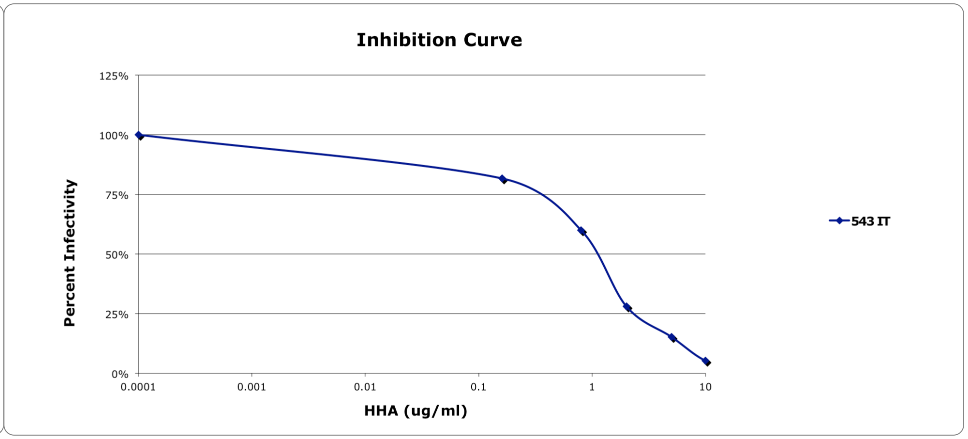
Inhibition Curve
Percent Infectivity
125%
100%
75%
50%
25%
0%
0.0001
0.001
0.01
0.1
1
10
HHA (ug/ml)
543 IT

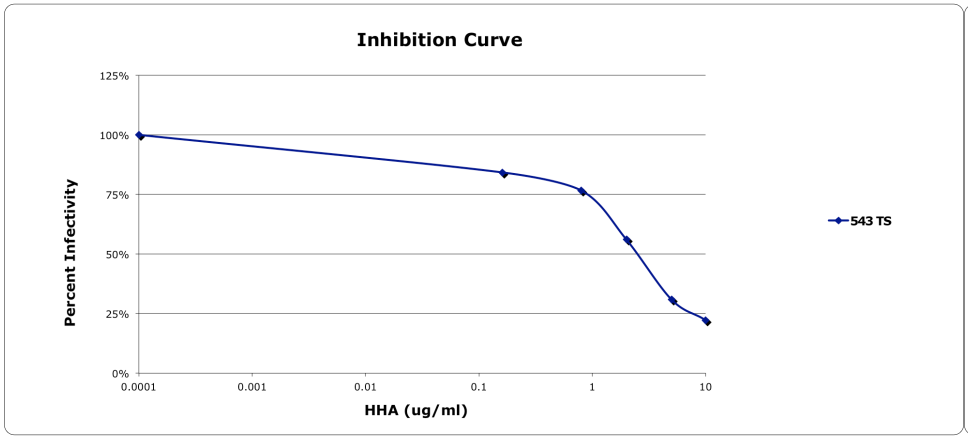
Inhibition Curve
Percent Infectivity
125%
100%
75%
50%
25%
0%
0.0001
0.001
0.01
0.1
1
10
HHA (ug/ml)
543 TS

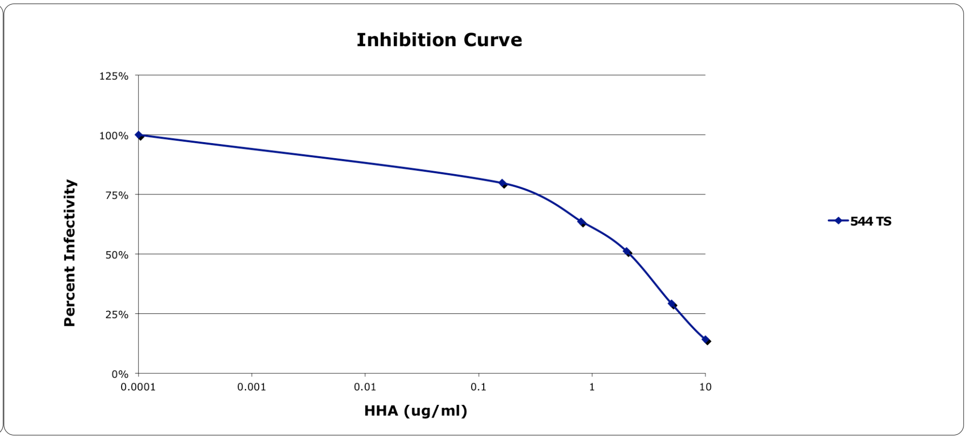
Inhibition Curve
Percent Infectivity
125%
100%
75%
50%
25%
0%
0.0001
0.001
0.01
0.1
1
10
HHA (ug/ml)
544 TS

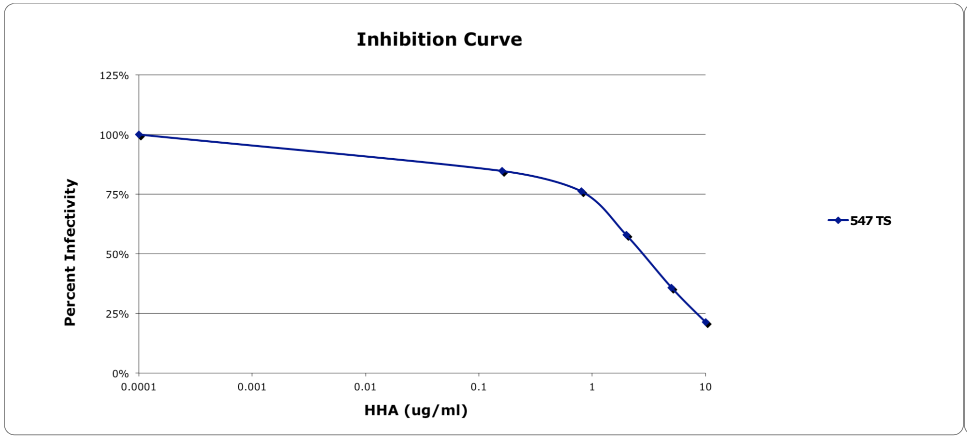
Inhibition Curve
125%
100%
75%
50%
25%
0%
Percent Infectivity
0.0001
0.001
0.01
0.1
1
10
HHA (ug/ml)
547 TS

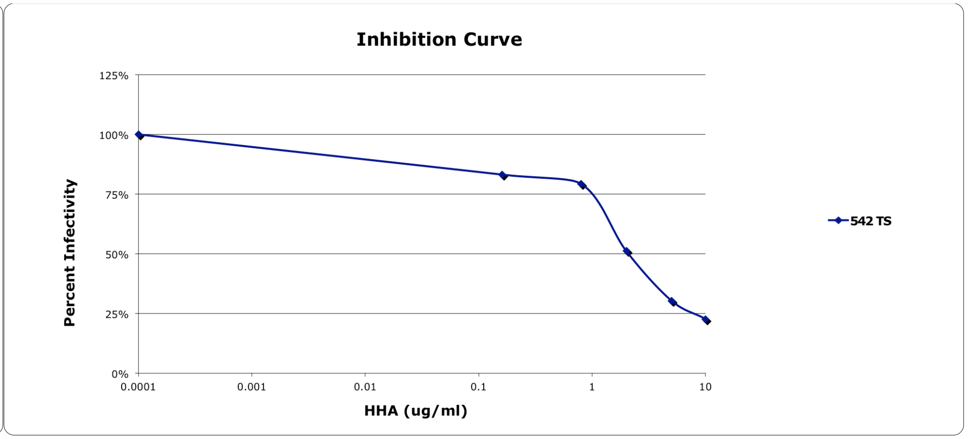
Inhibition Curve
125%
100%
75%
50%
25%
0%
Percent Infectivity
0.0001
0.001
0.01
0.1
1
10
HHA (ug/ml)
542 TS

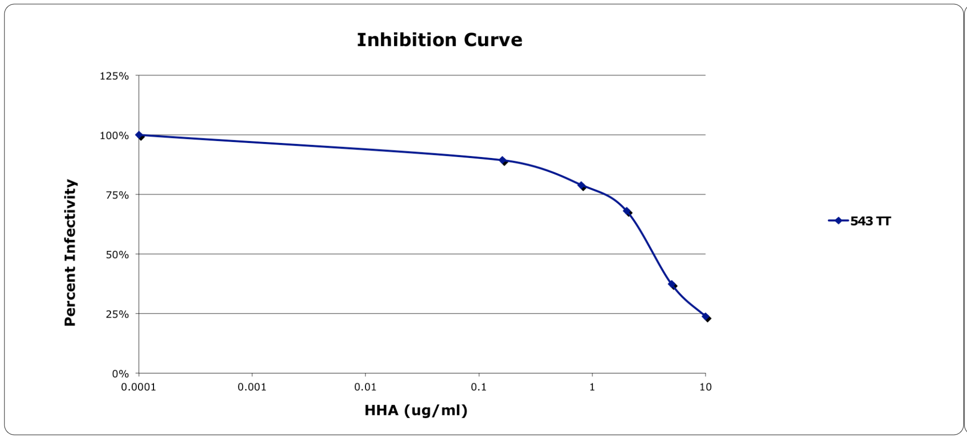
Inhibition Curve
125%
100%
75%
50%
25%
0%
Percent Infectivity
0.0001
0.001
0.01
0.1
1
10
HHA (ug/ml)
543 TT

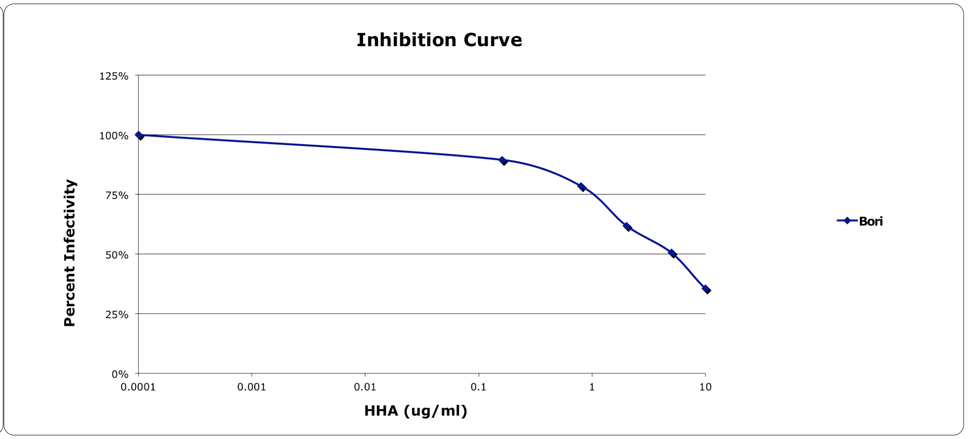
Inhibition Curve
125%
100%
75%
50%
25%
0%
Percent Infectivity
0.0001
0.001
0.01
0.1
1
10
HHA (ug/ml)
Bori

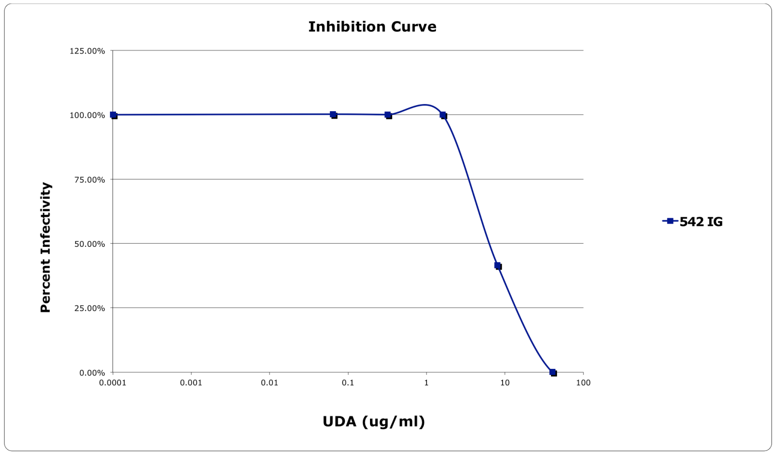
Inhibition Curve
Percent Infectivity
UDA (ug/ml)
542 IG

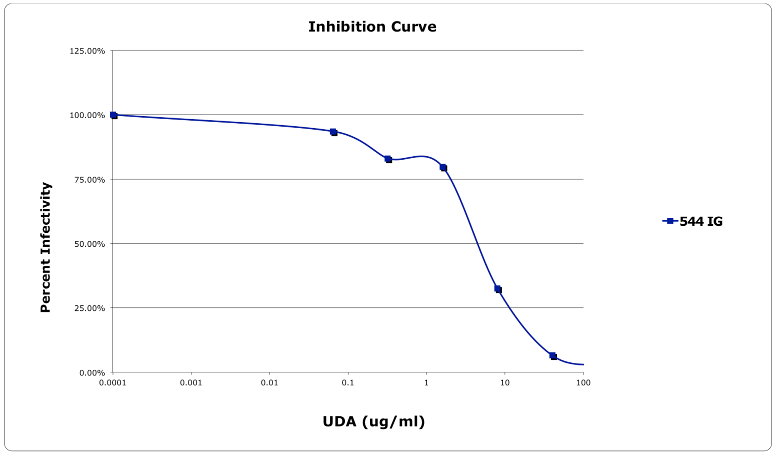
Inhibition Curve
Percent Infectivity
UDA (ug/ml)
544 IG

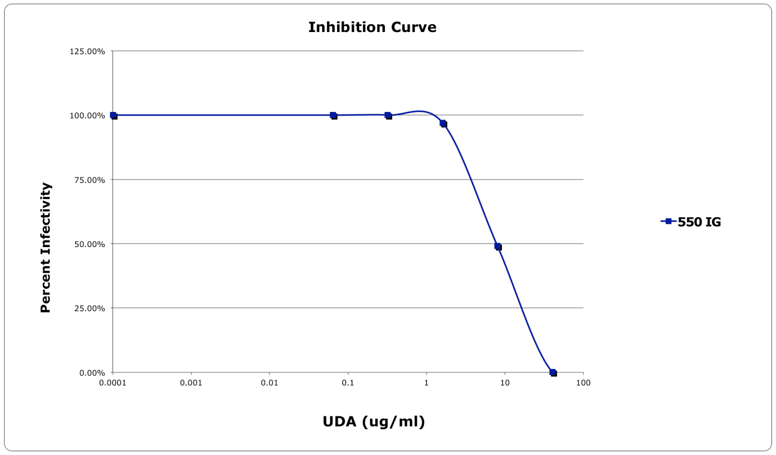
Inhibition Curve
Percent Infectivity
UDA (ug/ml)
550 IG

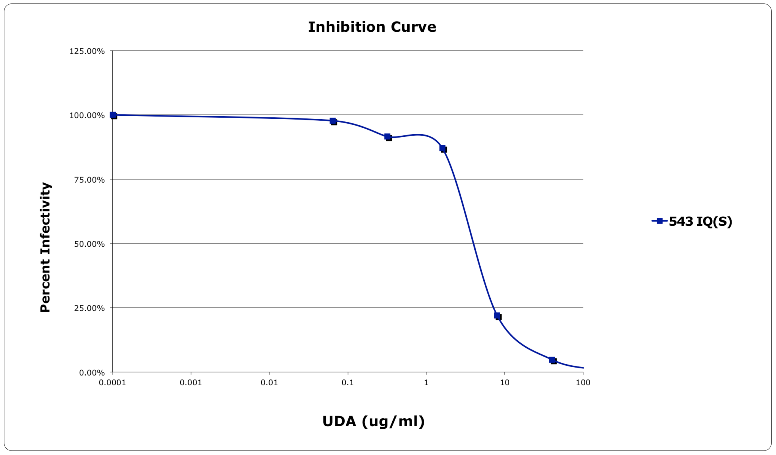
Inhibition Curve
Percent Infectivity
UDA (ug/ml)
543 IQ(S)

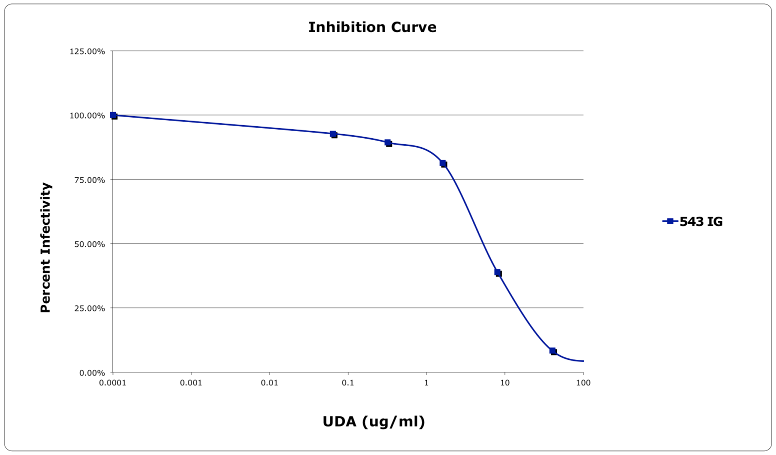
Inhibition Curve
Percent Infectivity
UDA (ug/ml)
543 IG

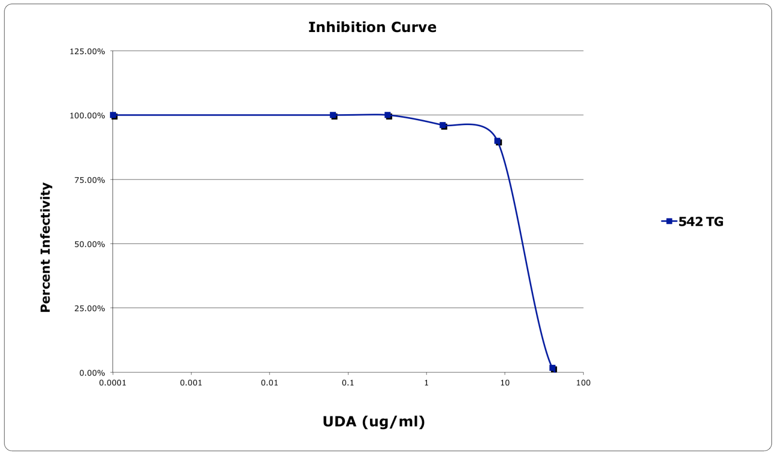
Inhibition Curve
Percent Infectivity
UDA (ug/ml)
542 TG

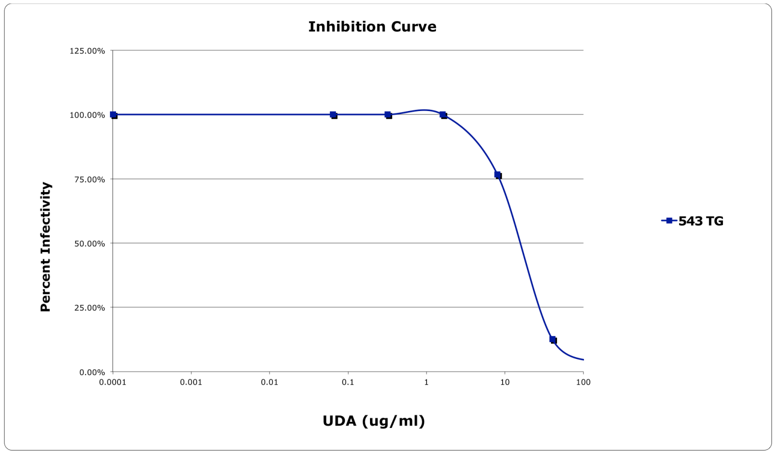
Inhibition Curve
125.00%
100.00%
75.00%
50.00%
25.00%
0.00%
Percent Infectivity
0.0001
0.001
0.01
0.1
1
10
100
UDA (ug/ml)
543 TG

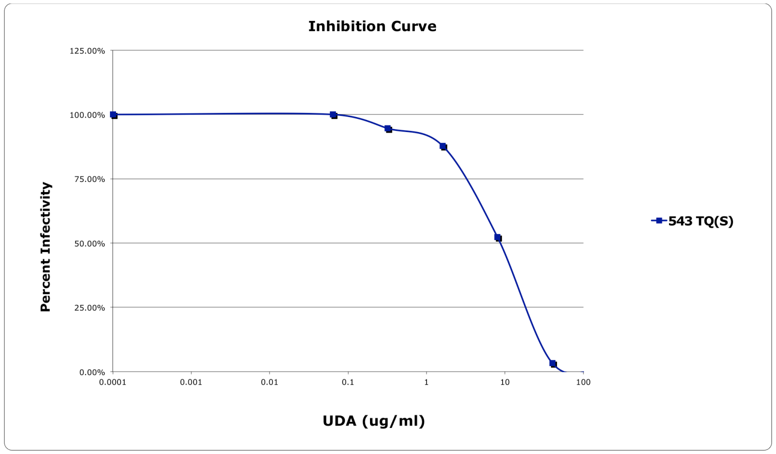
Inhibition Curve
125.00%
100.00%
75.00%
50.00%
25.00%
0.00%
Percent Infectivity
0.0001
0.001
0.01
0.1
1
10
100
UDA (ug/ml)
543 TQ(S)

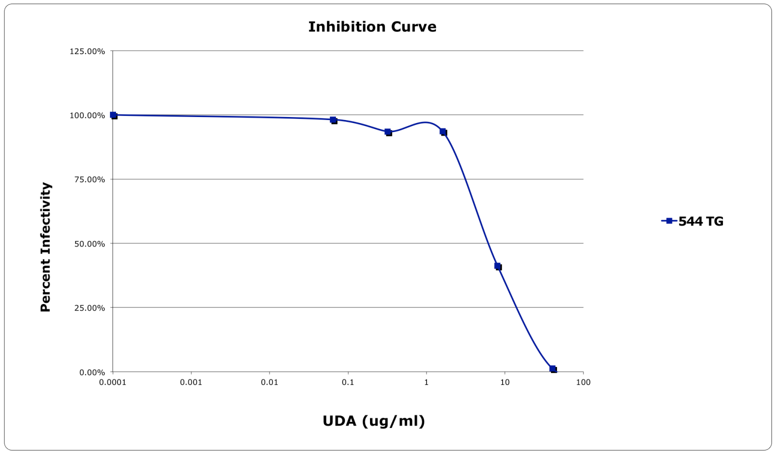
Inhibition Curve
125.00%
100.00%
75.00%
50.00%
25.00%
0.00%
Percent Infectivity
0.0001
0.001
0.01
0.1
1
10
100
UDA (ug/ml)
544 TG

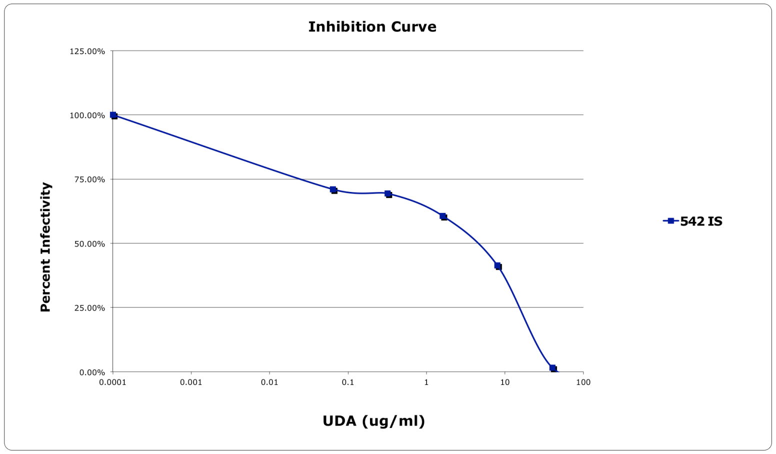
Inhibition Curve
125.00%
100.00%
75.00%
50.00%
25.00%
0.00%
Percent Infectivity
0.0001
0.001
0.01
0.1
1
10
100
UDA (ug/ml)
542 IS

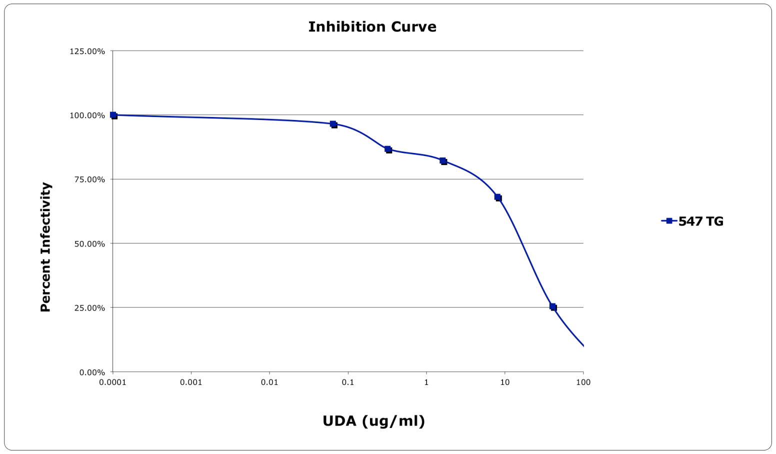
Inhibition Curve
125.00%
100.00%
75.00%
50.00%
25.00%
0.00%
Percent Infectivity
0.0001
0.001
0.01
0.1
1
10
100
UDA (ug/ml)
547 TG

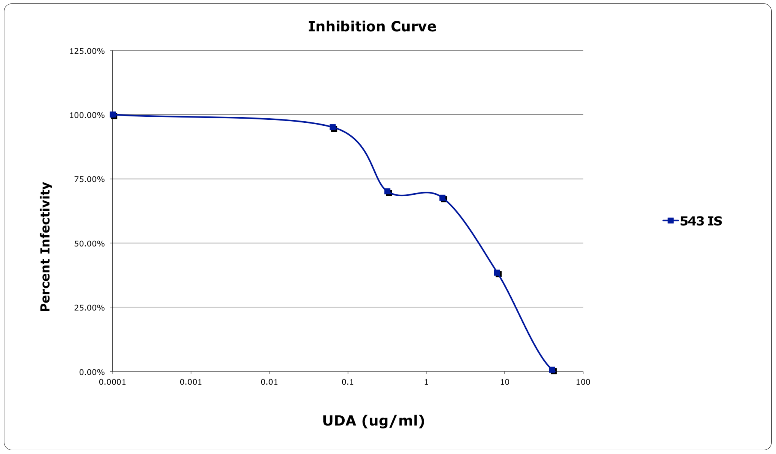
Inhibition Curve
125.00%
100.00%
75.00%
50.00%
25.00%
0.00%
Percent Infectivity
0.0001
0.001
0.01
0.1
1
10
100
UDA (ug/ml)
543 IS

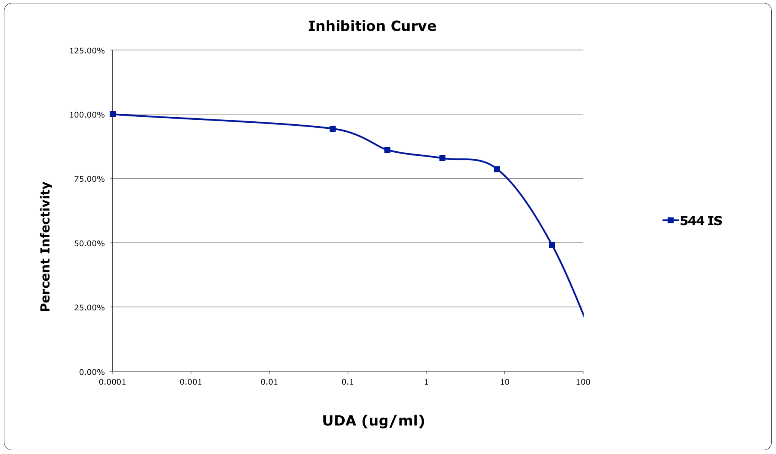
Inhibition Curve
Percent Infectivity
125.00%
100.00%
75.00%
50.00%
25.00%
0.00%
0.0001
0.001
0.01
0.1
1
10
100
UDA (ug/ml)
544 IS

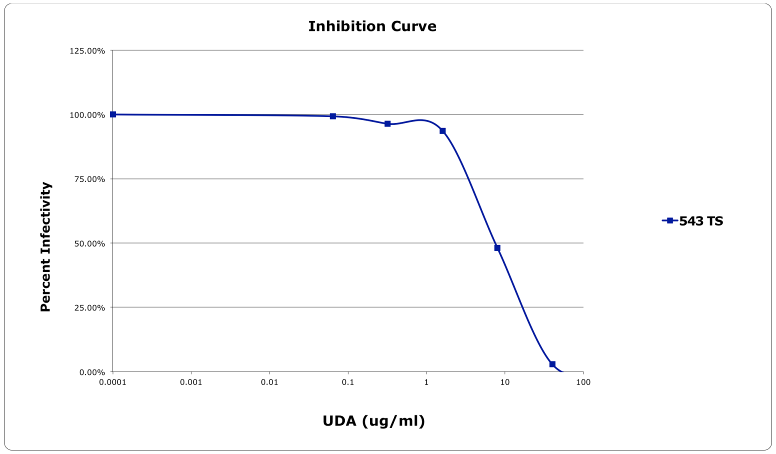
Inhibition Curve
Percent Infectivity
125.00%
100.00%
75.00%
50.00%
25.00%
0.00%
0.0001
0.001
0.01
0.1
1
10
100
UDA (ug/ml)
543 TS

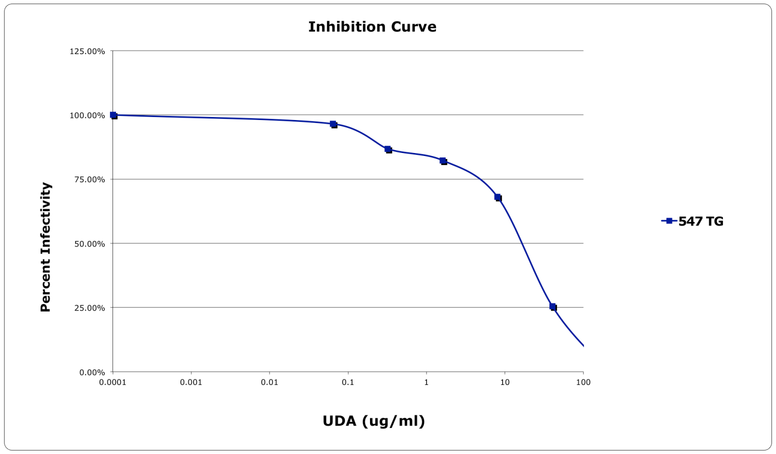
Inhibition Curve
Percent Infectivity
125.00%
100.00%
75.00%
50.00%
25.00%
0.00%
0.0001
0.001
0.01
0.1
1
10
100
UDA (ug/ml)
547 TG

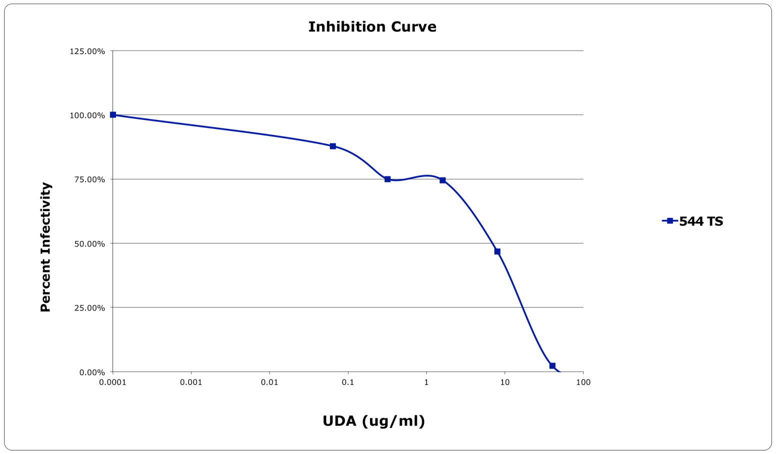
Inhibition Curve
Percent Infectivity
125.00%
100.00%
75.00%
50.00%
25.00%
0.00%
0.0001
0.001
0.01
0.1
1
10
100
UDA (ug/ml)
544 TS

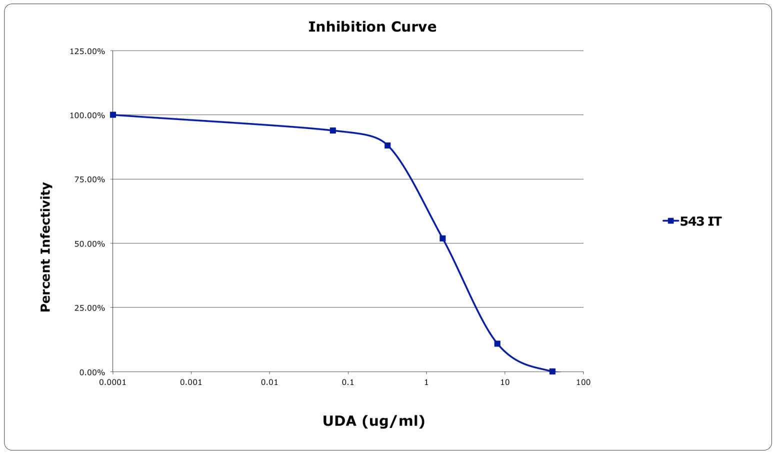
Inhibition Curve
Percent Infectivity
125.00%
100.00%
75.00%
50.00%
25.00%
0.00%
0.0001
0.001
0.01
0.1
1
10
100
UDA (ug/ml)
543 IT

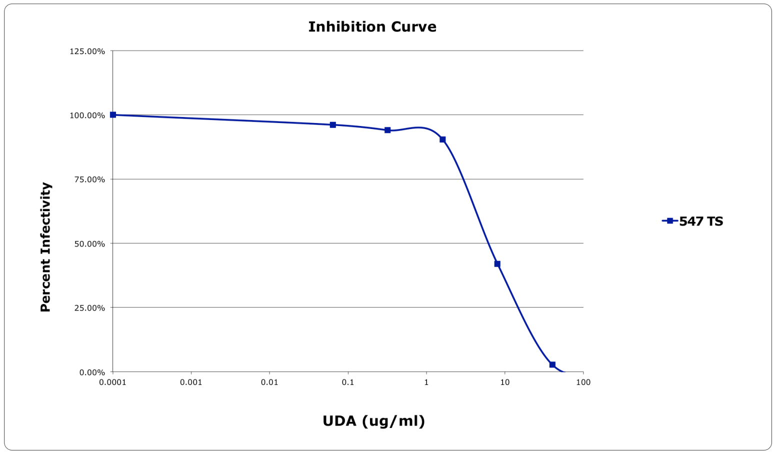
Inhibition Curve
Percent Infectivity
125.00%
100.00%
75.00%
50.00%
25.00%
0.00%
0.0001
0.001
0.01
0.1
1
10
100
UDA (ug/ml)
547 TS

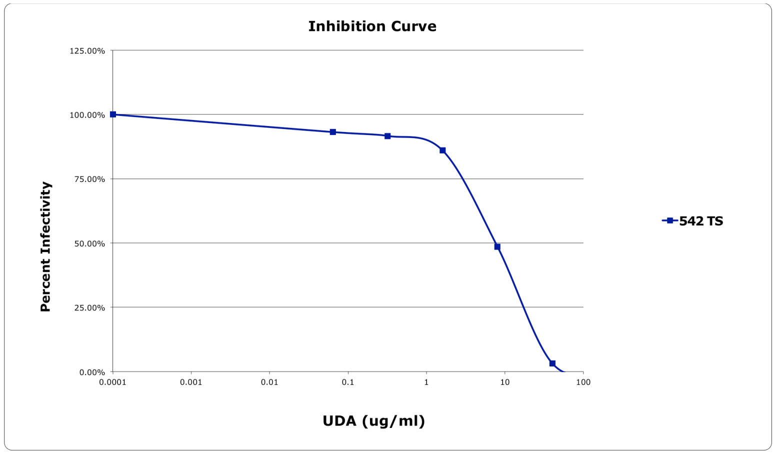
Inhibition Curve
125.00%
100.00%
75.00%
50.00%
25.00%
0.00%
0.0001
0.001
0.01
0.1
1
10
100
Percent Infectivity
UDA (ug/ml)
542 TS

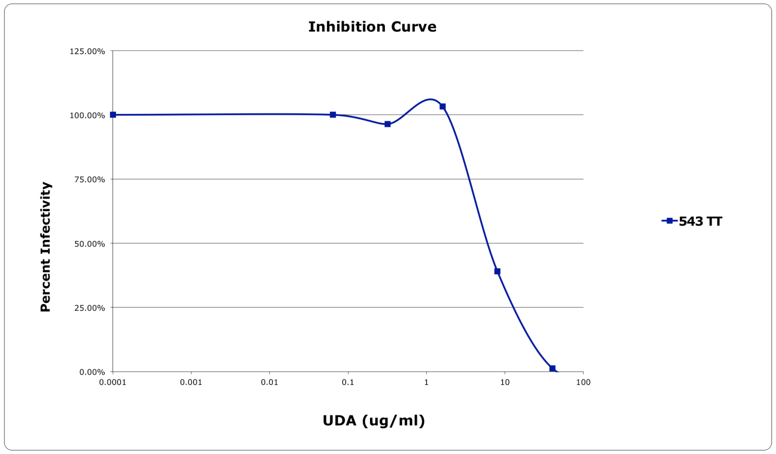
Inhibition Curve
125.00%
100.00%
75.00%
50.00%
25.00%
0.00%
0.0001
0.001
0.01
0.1
1
10
100
Percent Infectivity
UDA (ug/ml)
543 TT

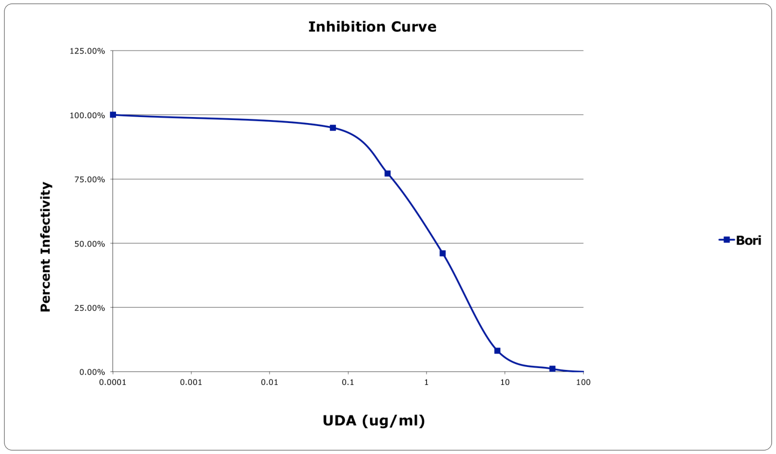
Inhibition Curve
125.00%
100.00%
75.00%
50.00%
25.00%
0.00%
0.0001
0.001
0.01
0.1
1
10
100
Percent Infectivity
UDA (ug/ml)
Bori
